# Alternative spliceosomal protein Eftud2 mediated *Kif3a* exon skipping promotes SHH-subgroup medulloblastoma progression

**DOI:** 10.1038/s41418-025-01512-9

**Published:** 2025-04-24

**Authors:** Ying Li, Liping Chen, Saisai Xue, Zhihong Song, Heli Liu, Hao Li, Wei Shen, Chen Zhang, Haitao Wu

**Affiliations:** 1 Department of Neurobiology, Beijing Institute of Basic Medical Sciences, Beijing, China; 2 School of Basic Medical Sciences, Beijing Key Laboratory of Neural Regeneration and Repair, Advanced Innovation Center for Human Brain Protection, Capital Medical University, Beijing, China; 3 Institute of Neuroscience, Hengyang Medical College, University of South China, Hengyang, Hunan China; 4 Key Laboratory of Neuroregeneration, Co-innovation Center of Neuroregeneration, Nantong University, Nantong, China; 5 Chinese Institute for Brain Research, Beijing, China

**Keywords:** CNS cancer, Oncogenes, Development

## Abstract

Alternative splicing plays a pivotal role in various facets of organogenesis, immune response, and tumorigenesis. Medulloblastoma represents a prevalent childhood brain tumor, with approximately one-third classified as the Sonic Hedgehog (SHH) subgroup. Nevertheless, the contribution of alternative splicing to medulloblastoma oncogenesis remains elusive. This investigation delineated an upregulation of the spliceosomal protein Eftud2 in the SHH-subgroup medulloblastoma mouse model and human medulloblastoma patients. Targeted ablation of *Eftud2* in granule precursor cells (GNPs) within the cerebellum prolonged the survival of SHH-subgroup medulloblastoma mice, indicating a putative association between Eftud2 expression and medulloblastoma prognosis. Functional assays unveiled that *EFTUD2* depletion in human medulloblastoma cells significantly curtailed cellular proliferation by impeding the activation of the SHH signaling pathway. Through multi-omics sequencing analysis, it was discerned that Eftud2 influences exons 10–11 skipping of *Kif3a*, a kinesin motor critical for primary cilia formation. Notably, exons 10–11 skipping in *Kif3a* augmented human medulloblastoma cell proliferation by potentiating the transcriptional activity of Gli2. These findings underscore a robust correlation between Eftud2 and SHH-subgroup medulloblastoma, emphasizing its regulatory role in modulating downstream transcription factors through the alternative splicing of pivotal genes within the SHH signaling pathway, thereby propelling the aggressive proliferation of SHH-subgroup medulloblastoma.

## Introduction

Alternative splicing constitutes a dynamic mechanism that enriches transcriptome complexity and proteome diversity in eukaryotic cells [1]. The precise orchestration of alternative splicing is mediated by the spliceosome, comprising five small nuclear ribonucleic acids (snRNAs), namely U1, U2, U4, U5, and U6, which assemble into small nuclear ribonucleoprotein particles (snRNPs), alongside over a hundred associated non-snRNP proteins [2]. Recent studies have underscored the significant involvement of alternative splicing in various cellular processes underlying malignant tumors, including proliferation [3], invasion [4], metastasis [5], and drug resistance [6]. Initial explorations of common human cancer mutations predominantly focused on U1 and U2 snRNPs [7–9]. However, the revelation of PRPF8 mutations in myeloid malignancies [10] has illuminated the contribution of U5 snRNPs to human cancers, encompassing genes such as EFTUD2, PRPF6, SNRNP40, DDX23, etc [9].

Eftud2, also recognized as elongation factor Tu GTP binding domain containing 2 or Snu114, stands as a highly conserved spliceosomal GTPase [2], pivotal in diverse biological processes. Its functional spectrum encompasses spliceosomal activation [11], developmental intricacies [12], oncogenic pathways [9], and immunological responses [13]. Notably, mutations in EFTUD2 have been pinpointed as causative factors of mandibulofacial dysostosis with microcephaly (MFDM), a syndrome typified by microcephaly, craniofacial anomalies, and neurological developmental aberrations [12, 14, 15]. Evidence from zebrafish and murine models has further substantiated the indispensable role of Eftud2 in neurodevelopment [16, 17]. Moreover, Eftud2 has emerged as a regulator in diverse tumor contexts. It exerts pro-apoptotic effects in breast cancer cells while fostering tumorigenesis in colitis-associated tumors and hepatocellular carcinoma through multifaceted mechanisms [18–20]. Nonetheless, the precise involvement of Eftud2 in central nervous system tumorigenesis remains enigmatic.

Medulloblastoma, a malignant embryonal brain tumor with a peak incidence in childhood, exhibits considerable molecular heterogeneity with the existence of at least four distinct subgroups—Wingless-type (WNT), Sonic Hedgehog (SHH), Group 3 (G3) and Group 4 (G4) [21]. These classifications are derived from distinct genetic alterations, variations in age at onset, and differences in prognosis [22–24]. Among these, the SHH-subgroup represents ~30% of clinical cases, predominantly driven by the over-activation of the SHH signaling pathway, which results in the malignant proliferation of granule neuron precursors (GNPs) [25]. Mutational patterns associated with SHH-subgroup medulloblastoma include loss-of-function mutations or deletions in *PTCH1* or *SUFU*, activating mutations in *SMO*, and amplifications of *GLI1/2* and *MYCN* [21, 26]. Recent studies have also highlighted the role of alternative splicing of key genes within the SHH signaling pathway, such as *Ptch1*, *Gli1/2* and *Sufu* [27], in modulating the behavior of SHH-subgroup medulloblastoma. Nonetheless, the role of critical alternative splicing regulatory proteins in influencing the development of SHH-subgroup medulloblastoma remains insufficiently explored.

This study investigates the regulatory role of alternative splicing in the development of SHH-subgroup medulloblastoma. Our bulk RNA sequencing analysis and immunohistochemical analysis revealed a significant upregulation of Eftud2 in both SHH-subgroup medulloblastoma mouse model and medulloblastoma tumors from patients. The specific ablation of *Eftud2* expression in GNPs within the SHH-subgroup medulloblastoma mouse model leads to a significant decrease in both the expression and transcriptional activity of Gli2, a critical component of the SHH signaling pathway [28–30]. Mechanistically, we identified that Eftud2 influences the expression and transcriptional activity of Gli2 by modulating the alternative splicing of the kinesin family member 3 A (*Kif3a*). Notably, Kif3a has been previously demonstrated to be essential for SHH signaling activation and the SHH-dependent expansion of cerebellar progenitors [31, 32]. These findings contribute to a deeper understanding of the underlying signaling mechanisms of SHH-subgroup medulloblastoma pathogenesis and propose potential novel targets for clinical drug intervention.

## Results

### Alternative splicing events are changed in 4 subgroups of medulloblastoma patients

**Fig. 1:**
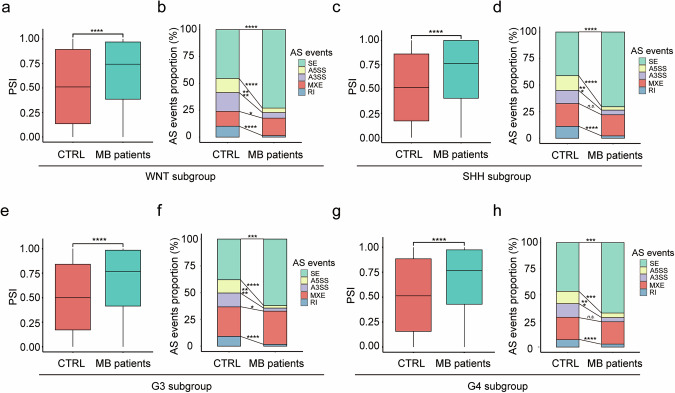
Alternative splicing events exhibit distinct alterations across four subgroups of medulloblastoma. Boxplot of percent sliced in (PSI) values for exons (with ≥10 junction reads) in cerebellum tissue (CTRL) (n = 3) from normal humans and the WNT (**a**, n = 3), SHH (**c**, n = 6), G3 (**e**, n = 9), G4 (**g**, n = 12) subgroup tumor tissue (MB patients) from medulloblastoma patients (Wilcoxon rank-sum test). Bar chart of percentage of alternative splicing events altered cerebellum tissue (CTRL) from normal humans and the WNT **b**, SHH **d**, G3 **f**, G4 **h** subgroup tumor tissue (MB patients) from medulloblastoma patients (unpaired *t*-test). ES Exon-skipping, A5SS Alternative 5′ splice site, A3SS Alternative 3′ splice site, MXE mutually exclusive exon, RI Retained intron. The data are presented as the mean ± SD (bar plots). *p < 0.05, **p < 0.01, ***p < 0.001, ****p < 0.0001, n.s. no significant.

Alternative RNA splicing has been demonstrated in oncogenesis of various cancers [33, 34], the precise contribution of spliceosomal proteins to the formation of medulloblastoma remains poorly understood. To elucidate the regulatory role of RNA alternative splicing in the pathogenesis of medulloblastoma, we compared mRNA alternative splicing events between normal human cerebellar tissue and tumor samples from medulloblastoma patients. Utilizing raw RNA sequencing data from childhood medulloblastoma tumors and normal cerebellum tissue obtained from the Sequence Read Archive (SRA) data (accession numbers: SRR26129072-SRR26129234), we assessed exon inclusion levels through the percent spliced in (PSI) index [35]. Our findings reveal a significantly increased transcriptome-wide splicing efficiency across all four subgroups of medulloblastoma tumors (Fig. 1a, c, e, g). Detailed examination of specific splicing events indicated an increase in exon skipping (ES) events, while alternative 5’ splice site (A5SS), alternative 3’ splice site (A3SS), and retained intron (RI) events were decreased across all four medulloblastoma subgroups (Fig. 1b, d, f, h). These results suggest that alternative splicing events are altered in medulloblastoma, potentially contributing to its pathogenesis.

### Upregulated expression of Eftud2 in SHH-subgroup medulloblastoma mice and patients

Among the four subgroups medulloblastoma, SHH-subgroup represents the largest patient cohort and is characterized by distinct molecular features and well-established in vivo mouse models [36, 37]. Consequently, we selected SHH-subgroup medulloblastoma model mice to investigate the role of RNA alternative splicing in medulloblastoma oncogenesis. SHH-subgroup medulloblastoma arises from abnormal activation of the SHH signaling pathway, often due to mutations in genes such as *PTCH1*, *SMOOTHENED* (*SMO*), and *SUFU*, which regulate tumor development [21, 38]. To study this, we employed the R26SmoM2 (*SmoM2*^fl/+^) mouse model, which carries a constitutively active W539L mutation in the mouse *Smo* gene (SmoM2) inserted into the Gt (ROSA) 26Sor locus [39]. *SmoM2*^*fl/+*^ mice were crossed with *Atoh1-Cre* mice to generate *Atoh1-Cre; SmoM2*^*fl/+*^ (*SmoM2*^*Atoh1*^) mice, which serve as a model for SHH-subgroup medulloblastoma characterized by SMO and the downstream SHH signaling pathway activation and triggering the formation of SHH-subgroup medulloblastoma in cerebellar GNPs [40]. The tumors in the *SmoM2*^*Atoh1*^ group exhibit rapid expansion at postnatal day 14 (P14), with tumor burden reaching its peak around postnatal day 30 (P30). Following this period of intense proliferation, *SmoM2*^*Atoh1*^ mice typically undergo significant disease progression, leading to mortality around postnatal day 35 (P35) [37].

**Fig. 2:**
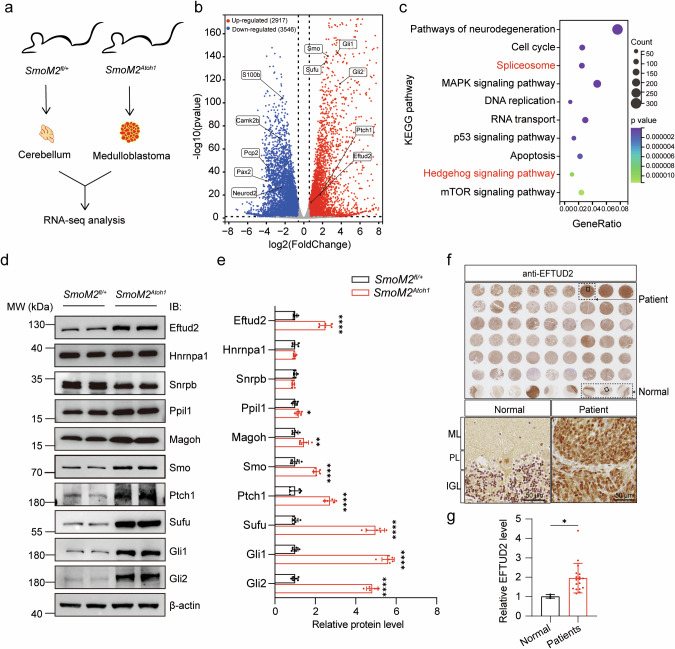
Upregulated expression of Eftud2 in SHH-subgroup of mouse and human medulloblastoma. **a** Schematic of the RNA-seq experiment. **b** Volcano plots of differential gene expression in *SmoM2*^*Atoh1*^ mouse tumors. **c** KEGG enrichment analysis of differential genes in tumor of *SmoM2*^*Atoh1*^ mice. Western blot validation **d** and quantification **e** of crucial Spliceosome proteins and Hedgehog signaling pathway factors in cerebellum of *SmoM2*^*fl/+*^ mice and tumor of *SmoM2*^*Atoh1*^ mice (n = 6 mice, 3 males, 3 females, unpaired *t*-test). Immunohistochemical staining **f** and quantification **g** showed a significant upregulation of EFTUD2 expression levels in human medulloblastoma tissue microarrays in medulloblastoma patients (normal, n = 3, medulloblastoma patients, n = 18, unpaired *t*-test). Molecular Layer (ML), the Purkinje Layer (PL), and the Internal Granule Layer (IGL). Scale bars = 50 μm. The data are presented as the mean ± SD (bar plots). *p < 0.05, **p < 0.01, ****p < 0.0001.

To capture the most active phase of tumor progression and ensure that transcriptomic data would reflect comprehensive tumor biology and cellular activity, RNA was extracted from the cerebellum of P30 *SmoM2*^*fl/+*^ mice and the tumors of P30 *SmoM2*^*Atoh1*^ mice for deep sequencing analysis (Fig. 2a). Comparative analysis revealed significant differential expression in 6463 genes between the tumor tissues of *SmoM2*^*Atoh1*^ mice and the cerebellum of *SmoM2*^*fl/+*^ mice, with 2917 genes upregulated and 3546 genes down-regulated (Fig. 2b). Pathway analysis indicated robust activation of both the Spliceosome and Hedgehog signaling pathways in the *SmoM2*^*Atoh1*^ mice (Fig. 2c). Further validation of key alterations in the Spliceosome and Hedgehog signaling pathways was conducted using quantitative PCR (qPCR) (Fig. S1a, b) and Western blot (WB) analyses (Fig. 2d, e). These analyses showed significant upregulation of Gli1 and Gli2, critical transcription factors in the SHH pathway, suggesting activation of SHH signaling in medulloblastoma. Additionally, Eftud2, a spliceosomal protein, exhibited marked upregulation, suggesting a potentially pivotal role in SHH-subgroup medulloblastoma oncogenesis. Immunohistochemical analysis of human medulloblastoma pathological tissue arrays demonstrated significantly higher levels of EFTUD2 in the cerebellar tumors of medulloblastoma patients compared to normal controls (Fig. 2f, g).

In human RNA-seq data from Gene Expression Omnibus (GEO, GSE37418) and SRA (SRR26129072-SRR26129234) data, *EFTUD2* expression was significantly upregulated in patients with SHH, G3, and G4 subgroups (Fig. S2a). Notably, SHH-subgroup medulloblastoma is associated with the lowest 5-year survival rate among these subgroups, indicating its high malignancy (Fig. S2b). Given this, we focused on analyzing EFTUD2 expression specifically in SHH-subgroup medulloblastoma patients. Further comparative studies revealed a correlation between elevated EFTUD2 levels and the SHH-subgroup-specific marker GAB1 [37], with high expression of EFTUD2 observed in both SHH-subgroup medulloblastoma mice (*SmoM2*^*Atoh1*^) and patient samples (Fig. S2c, d). These findings indicate that EFTUD2 likely plays a critical role in promoting proliferation and malignancy in SHH-subgroup medulloblastoma. Given the larger patient cohort and the high malignancy associated with the SHH-subgroup, we next focused on SHH-subgroup medulloblastoma model mice to further investigate the molecular regulatory role of EFTUD2 in tumor pathogenesis.

### Ablation of Eftud2 suppresses the tumor formation in SHH-subgroup medulloblastoma mice

**Fig. 3:**
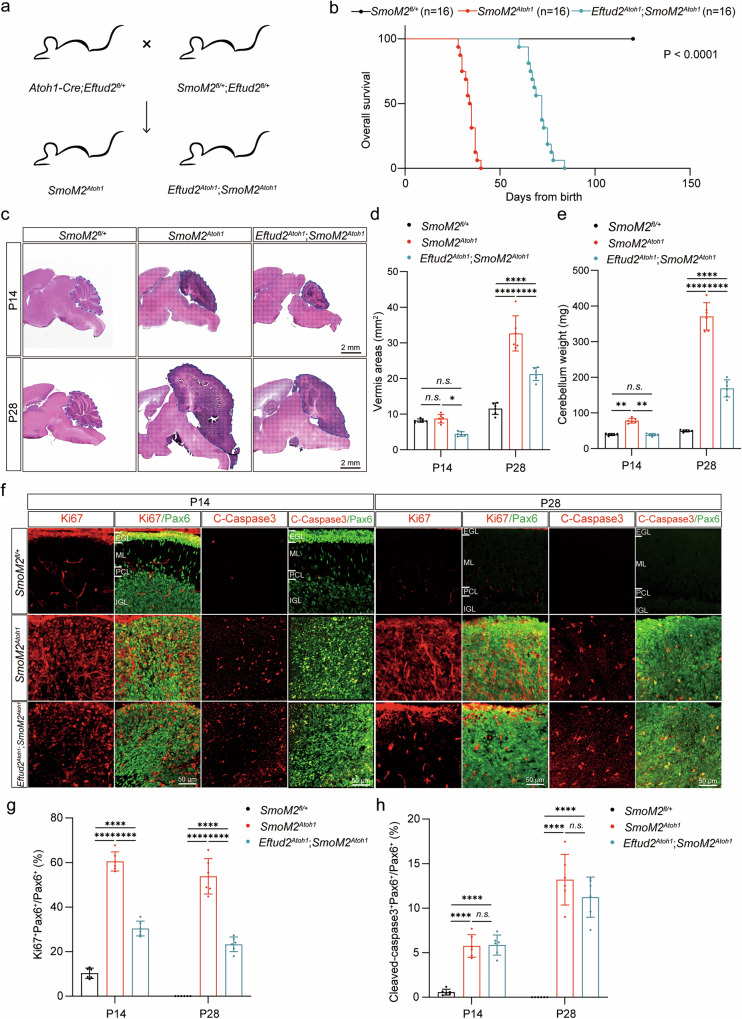
Eftud2 promotes the malignant proliferation of GNPs in SHH-subgroup medulloblastoma. **a** Schematic diagram for the construction of *Eftud2*^*Atoh1*^*; SmoM2*^*Atoh1*^ mice. **b** Overall survival of *SmoM2*^*fl/+*^ mice, *SmoM2*^*Atoh1*^ mice and *Eftud2*^*Atoh1*^*; SmoM2*^*Atoh1*^ mice (n = 16 mice, log-rank and Gehan-Breslow-Wilcoxon test). **c** HE staining of the sagittal section of the cerebellar vermis in *SmoM2*^*fl/+*^ mice, *SmoM2*^*Atoh1*^ mice and *Eftud2*^*Atoh1*^*; SmoM2*^*Atoh1*^ mice at P14 and P28. Scale bar = 2 mm. Quantification of sagittal vermis areas **d** and cerebellum weight **e** in *SmoM2*^*fl/+*^ mice, *SmoM2*^*Atoh1*^ mice and *Eftud2*^*Atoh1*^*; SmoM2*^*Atoh1*^ mice at P14 and P28 (n = 6 mice, 3 males, 3 females, one-way ANOVA and Bonferroni’s multiple comparisons test). **f** Immunofluorescent co-staining with anti-Pax6 and anti-Ki67 or anti-Cleaved-caspase3 in cerebellar vermis sagittal section from P14 and P28 *SmoM2*^*fl/+*^ mice, *SmoM2*^*Atoh1*^ mice and *Eftud2*^*Atoh1*^*; SmoM2*^*Atoh1*^ mice. Scale bars = 50 μm. Quantification of the Ki67^+^ and Pax6^+^ double-positive cells among the Pax6^+^ cells **g** and Cleaved-caspase3^+^ and Pax6^+^ double-positive cells among the Pax6^+^ cells **h** in *SmoM2*^*fl/+*^ mice, *SmoM2*^*Atoh1*^ mice and *Eftud2*^*Atoh1*^*; SmoM2*^*Atoh1*^ mice at P14 and P28 (C-Caspase3: Cleaved-caspase3, n = 6 mice, 3 males, 3 females, one-way ANOVA and Bonferroni’s multiple comparisons test). Molecular Layer (ML), the Purkinje Layer (PL), and the Internal Granule Layer (IGL). The data are presented as the mean ± SD (bar plots). *p < 0.05, **p < 0.01, ****p < 0.0001, n.s. no significant.

To delineate the functional role of Eftud2 in the SHH-subgroup medulloblastoma, a targeted knockout experiment was conducted in GNPs of *SmoM2*^*Atoh1*^ mice. *SmoM2*^*fl/fl*^ mice were crossed with *Eftud2*^*fl/fl*^ mice to generate *Eftud2*^*fl/+*^; *SmoM2*^*fl/+*^ heterozygotes. Concurrently, *Atoh1-Cre* mice were crossed with *Eftud2*^*fl/fl*^ mice to produce *Atoh1-Cre; Eftud2*^*fl/+*^ heterozygotes. These heterozygotes were interbred to generate *SmoM2*^*Atoh1*^ mice (*Atoh1-Cre*; *SmoM2*^*fl/+*^) and *Eftud2*^*Atoh1*^*; SmoM2*^*Atoh1*^ (*Atoh1-Cre*; *Eftud2*^*fl/fl*^; *SmoM2*^*fl/+*^) mice, facilitating the specific knockout of *Eftud2* in GNPs (Fig. 3a). Survival analysis revealed that *Eftud2*^*Atoh1*^*; SmoM2*^*Atoh1*^mice exhibited a two-fold increase in lifespan compared to *SmoM2*^*Atoh1*^ mice (Fig. 3b). Although *Eftud2*^*Atoh1*^*; SmoM2*^*Atoh1*^ mice developed medulloblastoma, hematoxylin and eosin (H&E) staining of sagittal cerebellar sections from these three genotypes demonstrated a significant reduction in tumor volume and cerebellar area in *Eftud2*^*Atoh1*^*; SmoM2*^*Atoh1*^ mice at postnatal days 14 (P14) and 28 (P28) compared to *SmoM2*^*Atoh1*^ mice (Fig. 3c, d). Tumor weight measurements further confirmed a substantial decrease in tumor burden in *Eftud2*^*Atoh1*^*; SmoM2*^*Atoh1*^ mice at both P14 and P28 relative to *SmoM2*^*Atoh1*^ mice (Fig. 3e). These findings suggest that Eftud2 plays a crucial role in tumor development, and its disruption leads to a marked reduction in tumor burden and extended survival in this medulloblastoma model.

### Eftud2 loss-of-function inhibits the malignant proliferation of GNPs in SHH-subgroup medulloblastoma mice and human medulloblastoma cells

To further investigate the role of Eftud2 on SHH-subgroup medulloblastoma, we performed immunofluorescence staining to assess both proliferation and apoptosis in the cerebellar tissue of *SmoM2*^*fl/+*^, and the tumors of *SmoM2*^*Atoh1*^ mice and *Eftud2*^*Atoh1*^*; SmoM2*^*Atoh1*^ mice. Pax6 is a marker of GNPs, which are highly proliferative during cerebellar development [41]. Since SHH-subgroup medulloblastoma originates from cerebellar GNPs, and Pax6 is often associated with poorly differentiated cells in medulloblastoma [42], we used Pax6 to label GNPs in cerebellum of *SmoM2*^*fl/+*^ mice and tumor tissue of *SmoM2*^*Atoh1*^ and *Eftud2*^*Atoh1*^; *SmoM2*^*Atoh1*^ mice. The analysis revealed a significant reduction in the proportion of Ki67^+^Pax6^+^ GNPs in *Eftud2*^*Atoh1*^*; SmoM2*^*Atoh1*^ mice relative to *SmoM2*^*Atoh1*^ mice at P14 and P28, indicating a decreased proliferative capacity in GNPs following *Eftud2* ablation (Fig. 3f, g). However, apoptosis analysis showed no significant differences in the proportion of apoptotic GNPs between the two groups (Fig. 3f, h), suggesting that Eftud2 primarily influences GNPs proliferation rather than apoptosis in the context of SHH-subgroup medulloblastoma.

To explore the impact of EFTUD2 on proliferation in SHH-subgroup human medulloblastoma, we performed *EFTUD2* knockdown using small interfering RNA (siRNA) in Daoy [43–45], UW228 [46–48], and ONS76 cells [49–51], which were widely used as representative models of the SHH-subgroup medulloblastoma and xenograft studies. *EFTUD2* knockdown significantly reduced cell proliferation, demonstrated by Cell Counting Kit-8 (CCK-8) and colony formation assays (Fig. S3a–e, Fig. S4a–e and j–n). Immunofluorescence staining further corroborated these findings, showing a marked decrease in the fraction of KI67^+^EFTUD2^+^ cells following *EFTUD2* silencing (Fig. S3f, g, Fig. S4f, g, o, p). Additionally, chromatin incorporation assays using 5-ethynyl-2’-deoxyuridine (EdU) demonstrated a notable reduction in the EdU^+^EFTUD2^+^ cell population after *EFTUD2*-knockdown (Fig. S3h, i). In contrast, apoptosis assays did not show significant changes in Cleaved-CASPASE3^+^EFTUD2^+^ and TUNEL^+^EFTUD2^+^ populations (Fig. S3j–m, Fig. S4h, i, q, r). These results suggest that while *EFTUD2* knockdown impairs cell proliferation, it does not substantially affect apoptosis in these medulloblastoma cell lines.

To determine whether these cell lines exhibit GNP-like characteristics, we labeled Daoy, UW228, and ONS76 cells with Pax6, a marker for GNPs. Following *EFTUD2* knockdown, we observed a significant reduction in the proportion of KI67^+^PAX6^+^ cells (Fig. S5a, b, e, f, i, j), but no change in the proportion of Cleaved-CASPASE3^+^PAX6^+^ cells (Fig. S5c, d, g, h, k, l). These findings indicate that *EFTUD2* knockdown significantly reduces the proliferation of PAX6^+^ cells without affecting their apoptosis. In vitro results from these human cell lines align with observations in GNPs from *Eftud2*^*Atoh1*^*; SmoM2*^*Atoh1*^ mice in vivo, further supporting the role of EFTUD2 in promoting the oncogenic proliferation of GNPs in SHH-subgroup medulloblastoma.

### Eftud2 loss-of-function suppresses the expression and transcriptional activity of Gli2 in SHH-subgroup medulloblastoma

**Fig. 4:**
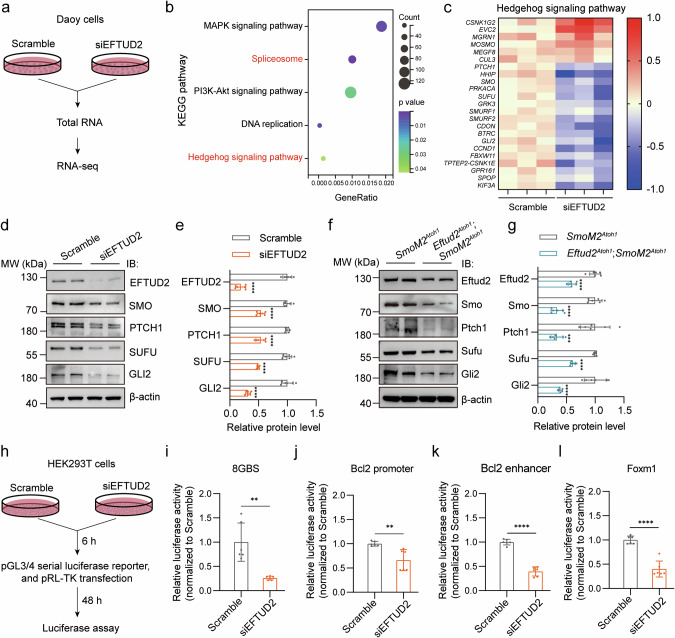
Knockdown of *EFTUD2* inhibits both the expression and transcriptional activity of GLI2 in SHH-subgroup medulloblastoma. **a** Schematic of the RNA-seq experiment. **b** KEGG enrichment analysis of differential genes in siEFTUD2 treated Daoy cells. **c** Heatmap showed decreased activity of the SHH signaling pathway in siEFTUD2 treated Daoy cells. Western blot validation **d** and quantification **e** of SHH signaling pathway essential factors in Scramble and siEFTUD2 treated Daoy cells (n = 6, unpaired *t*-test). Western blot validation **f** and quantification **g** of SHH signaling pathway essential genes in *SmoM2*^*Atoh1*^ mice and *Eftud2*^*Atoh1*^*; SmoM2*^*Atoh1*^ mice (n = 6 mice, 3 males, 3 females, unpaired *t*-test). Schematic of luciferase assay **h** and quantification of transcriptional activity of four different GLI2 downstream effectors **i**–**l** (8GBS, Bcl2 promoter, Bcl2 enhancer, and Foxm1, n = 6, unpaired *t*-test). The data are presented as the mean ± SD (bar plots). **p < 0.01, ***p < 0.001, ****p < 0.0001.

Building on the observed role of Eftud2 in promoting malignant proliferation of GNPs in SHH-subgroup medulloblastoma, as demonstrated in both in vivo and in vitro models, we next focused on elucidating the molecular mechanisms underlying Eftud2’s involvement in tumorigenesis. To further explore the mechanism by which EFTUD2 regulates the formation of human medulloblastoma, we chose the Daoy cells with a simpler genetic background for subsequent mechanistic research. Daoy cells were treated with either a scramble siRNA (Scramble) or siRNA targeting *EFTUD2* (siEFTUD2), followed by comprehensive molecular analyses. These analyses revealed significant disruptions in both the Spliceosome and Hedgehog signaling pathways, with key genes within the Hedgehog pathway showing substantial downregulation (Fig. 4a–c).

To validate the differential expression of Hedgehog pathway-related genes observed in RNA-seq analyses, we performed mRNA expression profiling in both Daoy cells and medulloblastoma mouse tissue samples. The results showed that *EFTUD2* knockdown led to consistent changes in the expression of several critical genes in the Hedgehog pathway, including *MOSMO*, *MEGF8*, *PTCH1*, *SMO*, *SUFU*, *BTRC*, *GLI2*, and *FBXW11*, across both the cell lines and tumor tissue samples. However, some genes exhibited varying expression patterns between the cell lines and mouse tissues (Fig. S6), which could reflect species-specific differences or the inherent distinctions between in vitro and in vivo environments. Moreover, the downregulation of *EFTUD2* resulted in a pronounced reduction in the expression of key Hedgehog pathway effectors across all models, including Daoy, UW228, and ONS76 cells, as well as in *Eftud2*^*Atoh1*^*; SmoM2*^*Atoh1*^ mice (Fig. 4d–g, Fig. S7a–d). Notably, GLI2, a critical transcription factor responsible for activating the SHH pathway [28–30], showed the most relative decrease, indicating that GLI2 might be a crucial target molecule in regulating the SHH signaling pathway by EFTUD2.

We conducted luciferase reporter assays to investigate further the function of EFTUD2 in the activation of SHH signaling pathway and the regulation of GLI2. These assays demonstrated that depletion of *EFTUD2* inhibits GLI2 transcriptional activity, as evidenced by reduced expression of several GLI2 downstream targets, including those associated with the 8GBS and key transcriptional regulatory regions, such as the Bcl2 promoter, Bcl2 enhancer, and Foxm1 (Fig. 4h–l) [37, 52–54]. Due to the low transfection efficiency in Daoy cells, HEK293T cells were firstly used for these experiments. Additionally, treatment of UW228 and ONS76 cells with either scramble RNA or siRNA targeting *EFTUD2* similarly resulted in decreased activity of GLI2 downstream targets in both cell lines (Fig. S7e–m).

Collectively, these findings support the hypothesis that *EFTUD2* downregulation inhibits GLI2 expression and its transcriptional activity in SHH-subgroup medulloblastoma, highlighting the crucial role of EFTUD2 in the activation of the SHH pathway and in promoting tumorigenesis.

### Identification of Eftud2-binding and mis-spliced target gene *Kif3a* in SHH-subgroup medulloblastoma mice

**Fig. 5:**
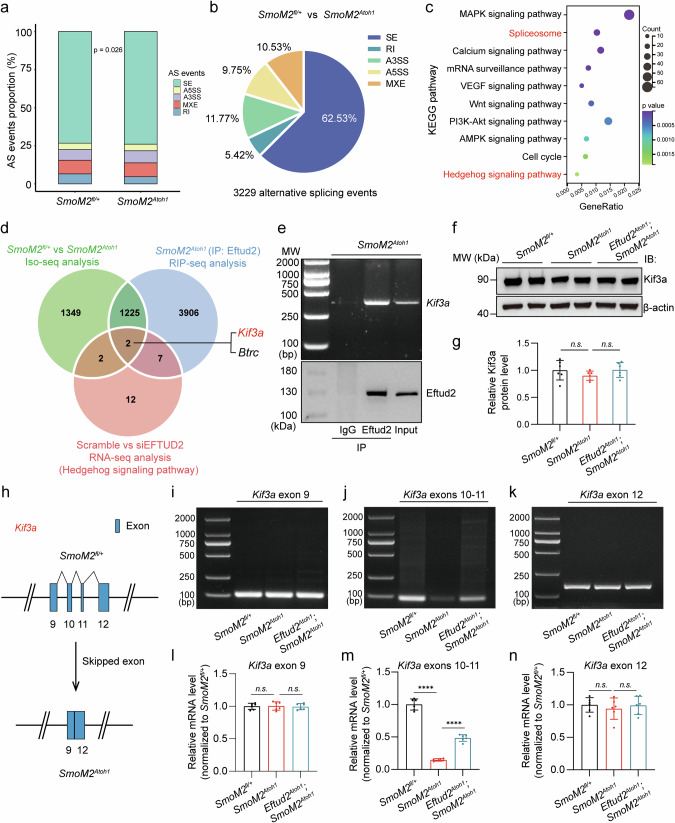
Eftud2 promotes the SHH signaling pathway activation *via* regulating *Kif3a* alternative splicing in vivo. **a** Bar chart depicting the percentage of alternative splicing events altered in cerebellar tissue from *SmoM2*^fl/+^ mice and tumor tissue from *SmoM2*^*Atoh1*^ mice, as identified in RNA-seq data (Wilcoxon rank-sum test). **b** Pie chart illustrating the distribution of significantly changed alternative splicing event types in *SmoM2*^*Atoh1*^ mouse tumors, as determined by Iso-seq. **c** KEGG enrichment analysis of alternative splicing genes in the Iso-seq data occurring in *SmoM2*^*Atoh1*^ mouse tumors. **d** Wayne diagram showing *Kif3a* and *Btrc* co-existing in the Iso-seq gene set of *SmoM2*^*Atoh1*^ mouse tumors, the RIP-seq (Eftud2-IP) gene set of S*moM2*^*Atoh1*^ mouse tumors, and the RNA-seq gene set (SHH signaling pathway) of siEFTUD2 treated Daoy cells. **e** Immunoprecipitation and RNA-binding protein precipitation verified that Eftud2 binds to both mRNA and protein of Kif3a in *SmoM2*^*Atoh1*^ mouse tumors. Western blot validation **f** and quantification **g** of Kif3a in *SmoM2*^*fl/+*^ mice cerebellum, *SmoM2*^*Atoh1*^ mice and *Eftud2*^*Atoh1*^*; SmoM2*^*Atoh1*^ mice tumors (n = 6 mice, 3 males, 3 females, one-way ANOVA and Bonferroni’s multiple comparisons test). **h** *Kif3a* exons 10–11 skipped in *SmoM2*^*Atoh1*^ mouse tumors. RT-PCR validation **i**–**k** and quantification **l**–**n** of Kif3a exon 9 **i**, **l** exons 10–11 **j**, **m** and exon 12 **k**, **n** in cerebellum of *SmoM2*^fl/+^ and *SmoM2*^*Atoh1*^ mouse tumors (n = 6 mice, 3 males, 3 females, one-way ANOVA and Bonferroni’s multiple comparisons test). The data are presented as the mean ± SD (bar plots). ****p < 0.0001, n.s. no significant. SE Skipped exon, A5SS Alternative 5′ splice site, A3SS Alternative 3′ splice site, MXE mutually exclusive exon, RI Retained intron.

Eftud2, a highly conserved spliceosomal GTPase, plays a critical role in spliceosome activation and alternative splicing events, which are essential for cellular function and diversity [55, 56]. These splicing events, including skipped exon (SE), retained intron (RI), alternative 5′ splice site (A5SS), alternative 3′ splice site (A3SS), and mutually exclusive exon (MXE), regulate gene expression and protein diversity [57]. Initial analysis of splicing events in *SmoM2*^*fl/+*^ and *SmoM2*^*Atoh1*^ mice, as indicated by RNA-seq, revealed significant alterations in splicing patterns (Fig. 5a). To investigate RNA alternative splicing in specific genes further, we performed isoform sequencing (Iso-seq) on the same cerebellar tissue from *SmoM2*^*fl/+*^ mice and tumor tissues from *SmoM2*^*Atoh1*^ mice used in the RNA-seq above. Iso-seq provided full-length transcripts, including the 5’UTR, 3’UTR, and poly-A tail, allowing for precise analysis of alternative splicing events. This analysis identified 3229 genes undergoing alternative splicing changes in *SmoM2*^*Atoh1*^ tumors compared to *SmoM2*^*fl/+*^ cerebellum, with SE accounting for the majority (62.52%) of events (Fig. 5b). KEGG pathway enrichment analysis of these 3229 genes revealed significant alterations in both Spliceosomal and Hedgehog signaling pathways (Fig. 5c).

To understand the role of Eftud2 in splicing regulation, we performed RNA immunoprecipitation (RIP) to isolate Eftud2-bound RNA from *SmoM2*^*Atoh1*^ tumor tissues, followed by RNA-seq analysis (RIP-seq). Comparative analysis of RIP-seq data, splicing events in *SmoM2*^*Atoh1*^ tumors, and Hedgehog pathway gene expression after *EFTUD2* knockdown in Daoy cells identified two significant genes: *Kif3a* and *Btrc* (Fig. 5d). *Kif3a* is crucial for the initiation and maintenance of SHH-subgroup medulloblastoma [58], whereas *Btrc* is not significantly associated with human medulloblastoma [59]. Subsequent RIP assays confirmed the interaction between Eftud2 and *Kif3a* pre-mRNA in tumors of *SmoM2*^*Atoh1*^ mice (Fig. 5e). Protein expression analysis of Kif3a revealed no significant differences in the cerebellar tissue of *SmoM2*^*fl/+*^ mice and the tumors of *SmoM2*^*Atoh1*^ mice and *Eftud2*^*Atoh1*^*; SmoM2*^*Atoh1*^ mice (Fig. 5f, g, Fig. S8). This result contrasts with results in Daoy, UW228, and ONS76 cells, where *EFTUD2* knockdown led to a reduction in KIF3A protein levels (Fig. S9), suggesting that the more complex in vivo tumor microenvironment may influence Kif3a stability. Additionally, Iso-seq analysis showed exons 10–11 skipping in the *Kif3a* gene in *SmoM2*^*Atoh1*^ tumors (Fig. 5h). Reverse transcription polymerase chain reaction (RT-PCR) assays confirmed significant downregulation of exons 10–11, but not exon 9 and exon 12, in *Kif3a* expression in *SmoM2*^*Atoh1*^ tumors, with partial rescue of exons 10–11 skipping observed in *Eftud2*^*Atoh1*^; *SmoM2*^*Atoh1*^ tumors (Fig. 5i–n, Fig. S10a–d).

Beyond *Kif3a*, we identified other alternative splicing changes in Hedgehog pathway genes using RT-PCR, such as the retention of intron 10 in *Kif7* and intron 15 in *Ptch2*, respectively. Intron retention in these genes was elevated in *SmoM2*^*Atoh1*^ tumors and reduced following *Eftud2* knockdown in *Eftud2*^*Atoh1*^; *SmoM2*^*Atoh1*^ mice (Fig. S10e–r), confirming the accuracy of Iso-seq results. Given that KIF3A is highly conserved between humans and mice (Fig. S11a), next we examined exon skipping in *KIF3A* in Daoy cells. Consistent with the in vivo findings, exons 10–11 skipping in *KIF3A* was partially rescued in Daoy cells treated with siRNA targeting *EFTUD2* compared to scramble siRNA, while exon 9 and exon 12 remained unaffected (Fig. S11b–g). These findings suggest that Eftud2 interacts with the RNA of *Kif3a* and modulates its exons 10–11 skipping, impacting the pathobiology of SHH-subgroup medulloblastoma in both murine models and humans.

### Exons 10–11 skipping in *Kif3a* promotes the proliferation of human medulloblastoma cells *via* enhancing the transcriptional activity of Gli2

To further investigate the role of *Kif3a* exon skipping in the oncogenesis of SHH-subgroup medulloblastoma, we performed a series of experiments using Daoy, UW228, and ONS76 cell lines. First, *KIF3A* was targeted for knockdown with specific small interfering RNA (siKIF3A), followed by modulation of *Kif3a* expression using lentiviral transduction. Lentiviral vectors (pGC-FU-3FLAG-CBh-gcGFP-IRES-puromycin) carrying either a murine full-length Kif3a resistant to siKIF3A (Kif3a^R^) or a truncated *Kif3a* with exons 10–11 deletion (Kif3a^ΔE10-11^) were employed. To explore the functional impact of exons 10–11 skipping on *Kif3a*, we measured the phosphorylation level of its active form based on prior studies [60]. Phosphorylation analysis revealed that the Kif3a^ΔE10-11^ variant exhibited elevated phosphorylation levels compared to Kif3a^R^ (Fig. S12), suggesting that exons 10–11 skipping enhances Kif3a activation.

**Fig. 6:**
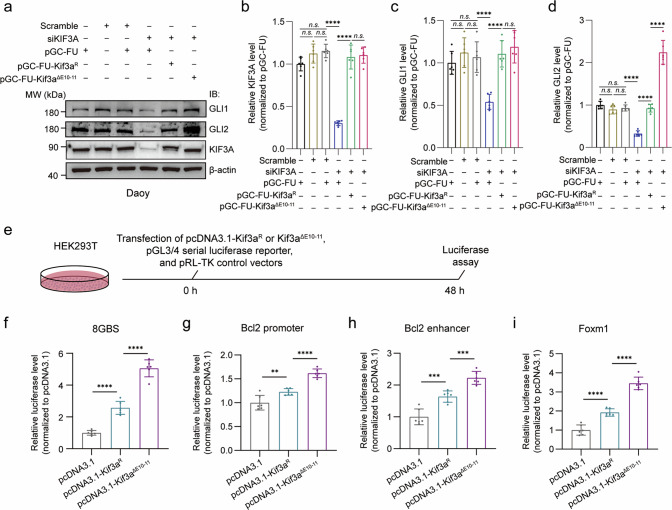
Exons 10–11 skipping in *Kif3a* enhances the transcriptional activity of Gli2. Western blot validation **a** and quantification **b**–**d** of GLI1, GLI2 and KIF3A in pGC-FU control, Scramble siRNA control, pGC-FU + Scramble siRNA control, pGC-FU + siKIF3A, siKIF3A + Kif3a^R^ and siKIF3A + Kif3a^ΔE10-11^ treated Daoy cells (n = 6, one-way ANOVA and Bonferroni’s multiple comparisons test). Schematic of luciferase assay **e** and quantification **f**–**i** of transcriptional activity of four different Gli2 downstream effectors (8GBS, Bcl2 promoter, Bcl2 enhancer, and Foxm1, n = 6, one-way ANOVA and Bonferroni’s multiple comparisons test). The data are presented as the mean ± SD (bar plots). **p < 0.01, ***p < 0.001, ****p < 0.0001, n.s. no significant.

To examine the functional consequences of *Kif3a* exons 10–11 skipping, we assessed the expression and transcriptional activity of GLI1 and GLI2 proteins in six experimental groups of Daoy, UW228, and ONS76 cells. Analysis of GLI1 and GLI2 expression showed a significant reduction in the siKIF3A-treated group compared to control groups, which included cells transfected with an empty lentiviral vector (pGC-FU) or/and treated with scramble siRNA (Fig. 6a, Fig. S13a, e). Notably, the introduction of Kif3a^ΔE10-11^ in the siKIF3A-treated group led to elevated expression of GLI2, mirroring results observed in *Eftud2*-overexpressing *SmoM2*^*Atoh1*^ mice (Fig. 6b–d, Fig. S13b–d, f–h). These findings suggest that exons 10–11 skipping in *Kif3a* enhances Gli2 expression, potentially driving SHH signaling.

Luciferase reporter assays were further employed to evaluate the impact of Kif3a^ΔE10-11^ on GLI2 transcriptional activity (Fig. 6e, Fig. S13i). The results demonstrated that the luciferase activity of four GLI2 downstream effectors was significantly higher in the Kif3a^ΔE10-11^ overexpressing group compared to the Kif3a^R^ overexpressing group, indicating enhanced transcriptional activity of GLI2 due to *Kif3a* exons 10–11 skipping (Fig. 6f–i, Fig. S13j–q). These findings suggest that *Kif3a* exons 10–11 skipping potentiates GLI2 activity, which could contribute to the malignant proliferation observed in SHH-subgroup medulloblastoma.

**Fig. 7:**
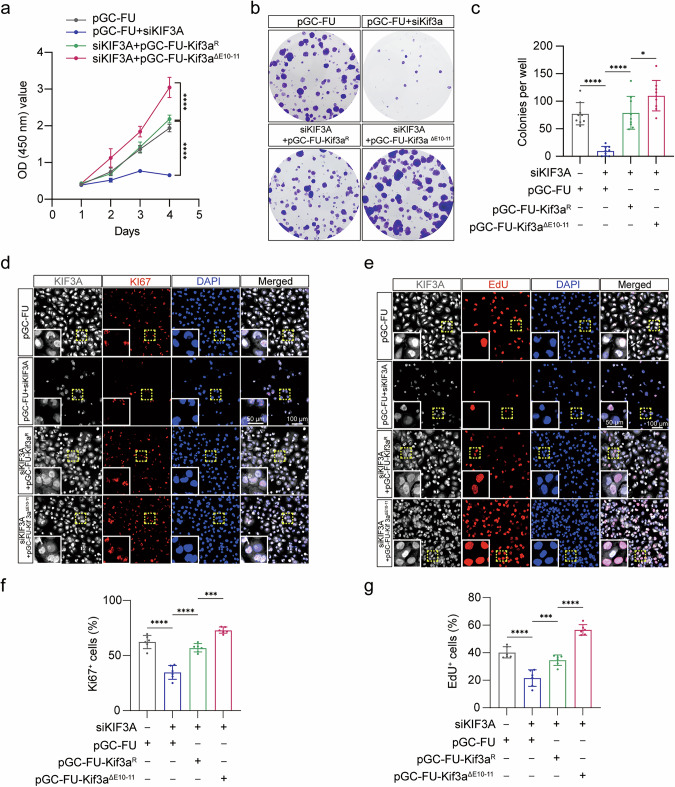
Exons 10–11 skipping in *Kif3a* promotes the proliferation of Daoy cells. The effect of Kif3a^R^ and Kif3a^ΔE10-11^ on cellular viability was detected by CCK-8 assays **a** and colony formation assays **b**, **c** respectively (n = 9, two-way ANOVA and Šídák’s multiple comparisons test for CCK-8 assays, one-way ANOVA and Bonferroni’s multiple comparisons test for colony formation assays). **d** Immunofluorescent co-staining with anti-KIF3A and anti-KI67 in pGC-FU control, pGC-FU+siKIF3A, siKIF3A+Kif3a^R^ and siKIF3A+Kif3a^ΔE10-11^ treated Daoy cells. Scale bars = 50 μm and 100 μm, respectively. **e** EdU incorporation assays were used to identify the cells in S phase in pGC-FU control, pGC-FU+siKIF3A, siKIF3A+Kif3a^R^ and siKIF3A+Kif3a^ΔE10-11^ treated Daoy cells. Scale bars = 50 μm and 100 μm, respectively. Quantification of KI67^+^ cells **f** and EdU^+^ cells **g** in pGC-FU control, pGC-FU+siKIF3A, siKIF3A+Kif3a^R^ and siKIF3A+Kif3a^ΔE10-11^ treated Daoy cells (one-way ANOVA and Bonferroni’s multiple comparisons test). The data are presented as the mean ± SD (bar plots). *p < 0.05, ***p < 0.001, ****p < 0.0001.

To further explore the role of exons 10–11 skipping in *Kif3a*-mediated tumorigenesis, we performed proliferation assays using Daoy, UW228, and ONS76 cell lines. Four experimental groups were assessed via CCK-8 and colony formation assays. Compared to the control group (pGC-FU), cells subjected to *Kif3a* knockdown (pGC-FU + siKIF3A) showed a marked reduction in both proliferation and colony formation (Fig. 7a–c, Fig. S14a–c, h–j). Interestingly, overexpression of Kif3a^ΔE10-11^ in the siKIF3A group (siKIF3A + Kif3a^ΔE10-11^) resulted in significant increases in both proliferation and colony formation compared to cells overexpressing full-length Kif3a^R^ (siKIF3A + Kif3a^R^) (Fig. 7a–c, Fig. S14a–c, Fig. S15a–c). Further investigation of proliferative dynamics using immunofluorescence and EdU staining confirmed these results. The proportion of KI67^+^ or EdU^+^ cells was reduced in the pGC-FU + siKIF3A group compared to the pGC-FU control group (Fig. 7d–g, Fig. S14d–g, Fig. S15d–g). Conversely, the siKIF3A + Kif3a^ΔE10-11^ group exhibited a substantial elevation in KI67^+^ and EdU^+^ cells compared to the siKIF3A + Kif3a^R^ group (Fig. 7d–g, Fig. S14d–g, Fig. S15d–g). These data provide compelling evidence that exons 10–11 skipping in *Kif3a* significantly enhances malignant tumor proliferation in SHH-subgroup medulloblastoma.

To further clarify whether *Kif3a* exons 10–11 skipping promotes cell proliferation through a Gli2-dependent mechanism, we treated Daoy, UW228, and ONS76 cells with either scramble siRNA (Scramble) or siRNA targeting *GLI2* (siGLI2). This treatment resulted in a significant reduction in GLI2 expression in siGLI2-treated cells (Fig. S16a–f). EdU staining was used to assess proliferation dynamics across these cell conditions. After *KIF3A* knockdown, Kif3a^ΔE10-11^ overexpression resulted in a marked increase in EdU^+^ cell populations compared to cells overexpressing Kif3a^R^ (siKIF3A + Scramble + Kif3a^ΔE10-11^ *vs* siKIF3A + Scramble + Kif3a^R^) (Fig. S16g–l). However, when both *KIF3A* and *GLI2* were knocked down (siKIF3A + siGLI2 + Kif3a^ΔE10-11^), the rate of EdU^+^ cells was comparable to that in the Kif3a^R^ group (siKIF3A + siGLI2 + Kif3a^R^), and both groups exhibited significantly fewer EdU^+^ cells compared to cells without *GLI2*-knockdown (Fig. S16g–l). These results indicate that *Kif3a* exons 10–11 skipping promotes medulloblastoma cell proliferation through a direct dependence on Gli2 transcriptional activity.

**Fig. 8:**
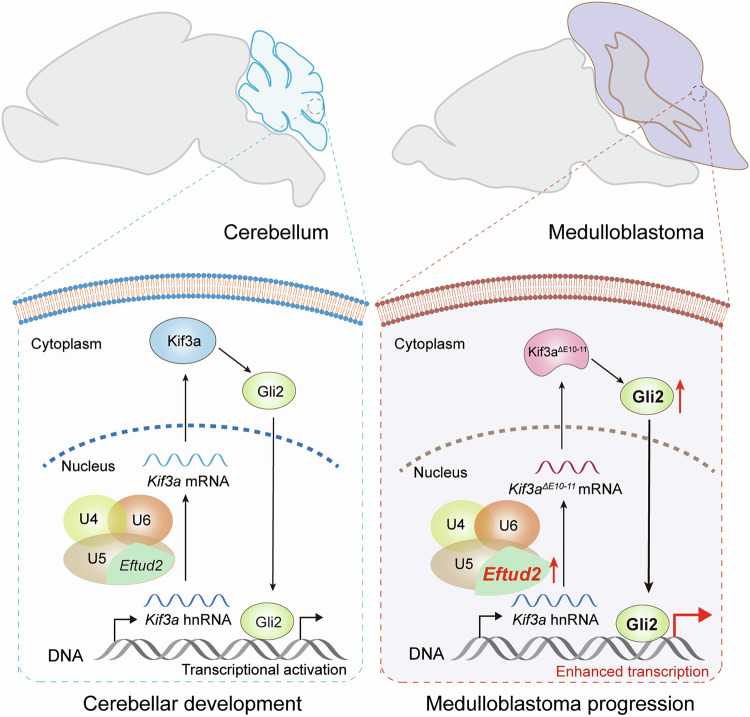
Graphic abstract of the role and signaling mechanism of Eftud2 in medulloblastoma progression. Conditional knockout of *Eftud2* in cerebellar granule precursor cells significantly extends survival in SHH medulloblastoma mice, highlighting its critical role in tumor progression. Mechanistically, *Eftud2* upregulation promotes medulloblastoma cell proliferation by activating the SHH pathway. This occurs in part through Eftud2-mediated *Kif3a* exons 10–11 skipping, which enhances Gli2 transcriptional activity and drives oncogenic signaling.

In summary, our findings demonstrate that exons 10–11 skipping in *Kif3a* enhances Gli2 transcriptional activity, which in turn, promotes the proliferation of SHH-subgroup medulloblastoma cells. These results highlight the potential therapeutic relevance of modulating Kif3a splicing in treating SHH-subgroup medulloblastoma (Fig. 8).

## Discussion

Disruptions in splicing factor activity or expression significantly reshape the transcriptome of brain tumors [27, 61–63]. In glioblastoma (GBM) and glioma stem cells, 58 spliceosome-associated RNA binding proteins (RBPs) were upregulated, with 21 linked to gliomagenesis, including SNRPB and SNRPG [63]. Overexpression of PTBP1 in GBM promotes progenitor-specific splicing, impeding tumor suppressor functions and enhancing tumor progression [64]. However, the role of RBPs in medulloblastoma is less well understood. Our study highlights the activation of the spliceosome pathway in SHH-subgroup medulloblastoma mouse models, underscoring the importance of alternative splicing in tumorigenesis. Notably, RBP expression patterns differ between medulloblastoma and GBM; for instance, SNRPB is downregulated in medulloblastoma (Fig. 2d). Meanwhile, the expression levels of some alternative splicing factors have changed, such as Ppil1 and Magoh (Fig. 2d). These alternative splicing factors might also have regulatory effects on the generation of medulloblastoma. Furthermore, we identified significant upregulation of EFTUD2, a core GTPase of the U5 snRNP complex, in both SHH-subgroup medulloblastoma mouse models and patient samples, and the role of EFTUD2 is not clear in other brain tumors. This suggests a pivotal role for alternative splicing and its core components in the pathogenesis of SHH-subgroup medulloblastoma, warranting further investigation into the dynamics of RNA splicing and RBP expression in brain tumor development.

Haploinsufficiency of EFTUD2 is the primary cause of MFDM [12], and loss-of-function mutations in Eftud2 lead to severe neurodevelopmental defects in zebrafish and mice, mediated by p53-dependent apoptosis in neural progenitor cells [16, 17, 65]. However, the role of EFTUD2 in the development and functional maintenance of other neuronal cell types, particularly in brain tumors, remains unclear. In non-neural contexts, Eftud2 upregulation has been observed in colonic tissues and infiltrating macrophages in colitis-associated cancer mouse models [20]. Inhibition of Eftud2 expression reduces NF-κB signaling, attenuating inflammatory and tumorigenic cytokine production [20]. In breast cancer, downregulation of EFTUD2, facilitated by SNW1 inhibition, induces apoptosis in cancer cells [19]. In SHH-subgroup medulloblastoma, we found EFTUD2 promotes tumor cell proliferation by activating the SHH signaling pathway and upregulating GLI2 expression and transcriptional activity. These results suggest the role of EFTUD2 in tumorigenesis varies among different cancer types, reflecting the potentially distinct signaling pathways involved. Unlike its effects in hepatocellular and breast cancers, EFTUD2 ablation does not significantly increase apoptosis in SHH-subgroup medulloblastoma (Fig. 3f, h, Fig. S3j–m, Fig. S4h, i, q, r), indicating that EFTUD2 regulates tumorigenesis through diverse mechanisms depending on the cancer context.

Several target molecules of Eftud2 have been identified in various contexts. In mice, cell-specific loss of Eftud2 function leads to increased exon 3 skipping in Mdm2, potentially over-activating the p53 pathway and inducing apoptosis in mouse neural crest cells [17]. In murine models of innate immunity and colitis-associated tumorigenesis, inhibition of Eftud2 results in alternative splicing of *MyD88*, generating the isoform *MyD88S*, which lacks exon 2 and acts as a negative regulator of TLR signaling [20, 66]. While previous studies on Eftud2-mediated splicing primarily relied on RNA sequencing, this method often identifies a broad range of splicing events without confirming binding or functional alterations of target molecules. To address this, we combined Iso-seq and RIP-seq to identify molecules potentially interacting with and spliced by Eftud2 in SHH-subgroup medulloblastoma mouse model in vivo (Fig. 5d). This combined approach enhances the precision of identifying Eftud2-regulated splicing events.

Our results show that Eftud2 facilitates exons 10–11 skipping of *Kif3a* and produces the Kif3a^ΔE10-11^ isoform in SHH-subgroup medulloblastoma mouse models. This isoform enhances the proliferation of Daoy, UW228, and ONS76 cells and increases GLI2 transcriptional activity. It should be noted that the interaction between Eftud2 and *Kif3a* pre-mRNA during the splicing process remains elusive. Previous studies have shown that Eftud2 interacts with Prp8, a component of U5 splicesome complex, which can directly bind to pre-mRNA [67, 68]. This suggests that Eftud2-mediated *Kif3a* exon skipping may occur through either a direct or indirect binding mechanism. Additionally, the mechanism by which the upregulation of Kif3a phosphorylation, resulting from exons 10–11 skipping, regulates Gli2 transcriptional activity also warrants further investigations. Furthermore, the aberrant splicing of Kif3a may exert regulatory effects through other intracellular signaling pathways beyond the SHH pathway. Notably, while Kif3a expression did not exhibit significant changes in *SmoM2*^*Atoh1*^ or *Eftud2*^*Atoh1*^; *SmoM2*^*Atoh1*^ mice, a reduction in *KIF3A* mRNA was observed in EFTUD2-knockdown human SHH-subgroup medulloblastoma cells in vitro. This discrepancy between in vivo and in vitro findings may reflect non-cell autonomous effects or the involvement of other regulatory mechanisms that influence Kif3a expression. Further studies are needed to clarify these possibilities.

Although our results here identified *Kif3a* as an Eftud2-mediated splicing target in mouse cerebellum, it cannot be ruled out that Eftud2 may regulate the alternative splicing of other genes in the SHH signaling pathway. Notably, the *Ptch1* gene, a key repressor of the SHH pathway, undergoes alternative splicing at its first exon, generating multiple isoforms—*Ptch1*, *Ptch1a*, *Ptch1b*, and *Ptch1c*—differentiated by variable first exons (1a, 1b, 1c, 1 d, and 1e) [69, 70]. The truncated isoform *Ptch1a* interacts with SHH family proteins without inhibiting the SHH pathway, whereas *Ptch1b* effectively represses Smo activity, and *Ptch1c* exhibits a weaker inhibitory effect [69, 70]. Moreover, the tGLI1 isoform, resulting from exon 3 skipping in *GLI1*, is found in Daoy cells [27] and promotes the upregulation of VEGF-A and CD24, driving the proliferation of breast cancer and glioblastoma cells [71–73]. In this study, we demonstrate that Eftud2 promotes malignant proliferation in SHH-subgroup medulloblastoma by interacting with Kif3a and modulating its alternative splicing to generate the Kif3a^ΔE10-11^ isoform (Figs. 5 and 7). Iso-seq revealed alternative splicing events in other genes involved in SHH pathway, such as *Ptch2* and *Kif7*, both of which show substantial intron retentions (Fig. S10). Although these genes do not interact with Eftud2 based on RIP-seq assay (Fig. 5), their spliced isoforms may also play significant roles in the pathogenesis of SHH-subgroup medulloblastoma. Further investigation is needed to explore whether a mutual regulatory relationship exists among the RNA splicing events of key SHH pathway regulators and their potential influence on medulloblastoma oncogenesis.

The link between splicing dysregulation and malignancy has driven the development of various preclinical strategies targeting splicing, some of which have advanced to clinical practice [74]. In SHH-subgroup medulloblastoma, small molecule inhibitors of SMO, such as itraconazole and arsenic trioxide, have demonstrated promising antitumor effects by targeting SMO and GLI2 [74]. Despite this, no studies have yet explored the use of drugs specifically targeting alternative splicing in medulloblastoma treatment, nor is it clear whether existing splicing inhibitors could impact medulloblastoma development. Our study highlights Eftud2 as a key regulator in SHH-subgroup medulloblastoma. However, some G3 and G4 subgroup medulloblastoma patients also exhibit upregulated expression of *EFTUD2* transcripts (Fig. S2a), though these changes in medulloblastoma biopsy at the protein level remain to be validated. Given the potential importance of EFTUD2 in medulloblastoma oncogenesis, RNA oligonucleotides and pharmacological inhibitors targeting U5 snRNP and GTPases like EFTUD2 could offer promising new therapeutic avenues for treating this malignancy.

In sum, our results illustrate a novel connection between Eftud2 and exon skipping of *Kif3a* and suggest RNA alternative splicing is a crucial mechanism to control neurogenesis and may function as a target for therapies against SHH-subgroup medulloblastoma.

## Materials and methods

### Animals

*Atoh1-Cre* (JAX: 011104) and *R26SmoM2* (*SmoM2*^*fl/+*^) (JAX:005130) were purchased from the Jackson laboratory. And *Eftud2*^*fl/fl*^ was a gift from Dr. Guojiang Chen at the Beijing Institute of Pharmacology and Toxicology. All mice were previously generated and maintained on a C57BL/6J background [16, 37, 65, 75, 76]. *Eftud2*^*fl/fl*^ mice were crossed with *Atoh1-Cre* mice and *SmoM2* ^*fl/fl*^ mice to produce *Atho1-Cre; Eftud2*^*fl/+*^ and *Eftud2* ^*fl/+*^*; SmoM2*^*fl/+*^ heterozygotes. A subsequent breeding resulted in the production of *Atho1-Cre; SmoM2*^*fl/+*^ (*SmoM2*^*Atoh1*^) and *Atho1-Cre; SmoM2*^*fl/+*^*; Eftud2*^*fl/fl*^ (*SmoM2*^*Atoh1*^*; Eftud2*^*Atoh1*^) mice. These mice were maintained in an environment with a 12-hour light/dark cycle and were provided ad libitum access to water and rodent chow. Breeding was conducted between 8-12 weeks of age. For all experiments, except the survival analysis, three male and three female mice from each group were randomly selected for further analysis. And the collection and morphological analysis of experimental animals were performed by different experimenters blinded to the genotypes. All animal experiments were conducted with the approval of the Institutional Animal Care and Use Committee (IACUC) of the Beijing Institute of Basic Medical Sciences. Genotyping was performed via PCR analysis of murine tail DNA with the oligonucleotide primer sequences specified in Table S1.

### Hematoxylin-Eosin (HE) staining

Cerebellar and tumor tissues were fixed in 4% paraformaldehyde (PFA) for 24 h, followed by sequential dehydration in 15% and 30% sucrose-PBS solutions, each for 48 h. These tissues were then sectioned parasagittally at a thickness of 30 μm using a cryostat microtome. Subsequently, the sections were stained using the Hematoxylin and Eosin Staining Kit (Beyotime, Catalogue No. C0105S, Shanghai, China) according to the manufacturer’s instructions. Histological sections were imaged using the TissueFAXS imaging system from TissueGnostics, and quantitative analysis was performed using ImageJ software version 9.0.

### Immunofluorescence and immunohistochemistry staining

Immunofluorescence and immunohistochemistry staining procedures were performed in accordance with methods previously described in the literature [37, 77]. For immunofluorescence, frozen tissue sections and fixed cells were subjected to a series of washes with PBS and 1% PBST. Nonspecific binding sites were blocked using a solution of 3% bovine serum albumin (BSA) in 0.5% PBST for one hour at room temperature, followed by an overnight incubation at 4 °C with primary antibodies diluted in 3% BSA. The primary antibodies used were: Eftud2 (Abcam, ab188327, 1:1000, Boston, USA), Ki67 (BD Biosciences, 550609, 1:500, New Jersey, USA), Pax6 (MBL, PD022, 1:1000, Tokyo, Japan), Cleaved-caspase3 (Cell Signaling Technology, 9661, 1:500, Boston, USA), Kif3a (Santa Cruz, sc-376680, 1:50, Texas, USA), Kif3a (Abcam, ab24626, 1:200, Boston, USA), Gli2 (Santa Cruz Biotechnology, sc-271786, 1:100, Texas, USA), and GAB1 (Abcam, ab59362, 1:200, Boston, USA). Following this, sections were washed thrice with 0.3% PBST for 10 min each and incubated with secondary antibodies: Alexa Fluor 568-conjugated goat anti-mouse IgG (Biotium, 20100, 1:500, California, USA), Alexa Fluor 488-conjugated goat anti-rabbit IgG (Biotium, 20012, 1:500, California, USA), Alexa Fluor 633-conjugated donkey anti-rabbit IgG (Biotium, 20125, 1:500, California, USA), and Alexa Fluor 568-conjugated goat anti-rabbit IgG (Biotium, 20102, 1:500, California, USA). Nuclei were counterstained with DAPI (ZSGB-bio, ZLI-9557, Beijing, China). Imaging was conducted using an Olympus FV-1200 confocal microscope and analyzed using Imaris software version 9.0.1.

The tissue microarray slides of human medulloblastoma were purchased from Avilabio (DC-Bra01022) and Bioaitech (N035Cb01), and the patient information of the medulloblastoma was specified in Table S2. For immunohistochemistry, after primary antibody incubation, the tissue microarray slides were processed using a 2-step universal immunoperoxidase (HRP) kit (ZSGB-bio, PV-8000, Beijing, China) and an enhanced DAB chromogenic kit (ZSGB-bio, ZLI-9018, Beijing, China) for signal detection. Images were acquired using the TissueFAXS imaging system (TissueGnostics, Vienna, Austria).

Immunofluorescence and immunohistochemistry staining intensity was quantified using the ImageJ software version 9.0. The relative intensity expression of the test group was normalized to baseline levels set by the control group.

### Western blot

Mouse cerebellum, tumor tissues, and cells (Daoy, UW228, and ONS76) were harvested and lysed using RIPA lysis buffer (Thermo Fisher Scientific, 89900, Waltham, USA) supplemented with 1% protease inhibitor cocktail (MCE, HY-K0010, New Jersey, USA). The protein lysates were centrifuged at 12,000 rpm at 4 °C for 15 min, and the supernatants were collected. Protein concentration was quantified using a BCA protein assay kit (Thermo Fisher Scientific, 23227, Waltham, USA). The proteins were then subjected to SDS-PAGE and transferred onto polyvinylidene difluoride (PVDF) membranes (Millipore, IPFL00010, Massachusetts, USA). After blocking, the membranes were incubated overnight at 4 °C with primary antibodies and then for 2 h at room temperature with horseradish peroxidase-conjugated secondary antibodies. Visualization was achieved using enhanced chemiluminescence (Applygen Technologies, P1000, Beijing, China), and the bands were detected with the MiniChemi610 Chemiluminescence Imaging System (Beijing SageCreation, China). The grayscale value of protein bands was measured using ImageJ software version 9.0. Target protein expression levels were normalized to the housekeeping gene β-actin for each sample. The protein expression of the control group was set as the baseline (value = 1), with relative expression levels calculated for test samples.

The primary antibodies used for the western blot analysis included: Eftud2 (Sigma, HPA022021, 1:3000, Saint Louis, USA), β-actin (Sungene Biotech, KM9001T, 1:3000, Tianjin, China), SMO (Santa Cruz Biotechnology, sc-166685, 1:100, Texas, USA), Ptch1 (Novus, NBP1-71662, 1:1000, Saint Louis, USA), Gli1 (Santa Cruz Biotechnology, sc-515751, 1:100, Texas, USA), Gli2 (Santa Cruz Biotechnology, sc-271786, 1:100, Texas, USA), Sufu (Cell Signaling Technology, 2522, 1:2000, Boston, USA), Hnrnpa1 (Abclonal, A11564, 1:1000, Wuhan, China), Snrpb (Abclonal, A2009, 1:1000, Wuhan, China), Ppil1 (Abclonal, A14892, 1:1000, Wuhan, China), Magoh (Abclonal, A6035, 1:1000, Wuhan, China), and Kif3a (Abcam, ab11259, 1:1000, Boston, USA).

### Immunoprecipitation and Immunoblotting

Mouse tumor and HEK293T cells were collected and lysed in a buffer containing 50 mM Tris (pH 7.5), 150 mM NaCl, 10 mM MgCl2, 1% Triton X-100, 10% glycerol, 1 mM EDTA, 1 mM PMSF (Beyotime, ST507, Shanghai, China), and 1% protease inhibitors (Thermo Fisher Scientific, A32965, Waltham, USA). Lysates were centrifuged at 12,000 rpm for 15 min at 4 °C. Immunoprecipitation was performed using primary antibodies (1ug/ml lysate) and protein A/G magnetic agarose beads (Thermo Fisher Scientific, 20423, Waltham, USA) overnight. The immunocomplexes were washed 3 times with a washing buffer (50 mM Tris-HCl, pH 7.5, 150 mM NaCl, 1 mM EDTA, 1% Triton X-100) before analysis by immunoblotting, and the subsequent experiments were the same as the Western blot. Primary antibodies used for immunoprecipitation are as follows: Eftud2 (Sigma, HPA022021, Saint Louis, USA), Kif3a (Abclonal, A6639, Wuhan, China), pan Phospho-Serine/Threonine (Abclonal, AP1067, Wuhan, China).

### Protein phosphorylation level assay

The protein phosphorylation level assay was conducted using a phosphorylation detection kit (Sangon Biotech, C500061, Beijing, China) following the manufacturer’s protocol. Briefly, the assay for protein phosphorylation levels involved preparing 18 wells, divided into standard and sample groups (wells 1–7 for standards and 8–9 for samples, each in duplicate). Samples were diluted or concentrated as needed, and 50 μL of either phosphorylated standard protein solution or sample was added to each well. Next, 50 μL of 2.0 mol/L NaOH was added, mixed, and incubated at 65 °C for 30 min. After cooling to room temperature, 50 μL of 4.7 mol/L HCl was added and mixed, followed by 50 μL of detection solution. The mixture was incubated at room temperature for 30 min with intermittent mixing. OD values at 620 nm were measured with the well-0 as the blank. A standard curve was plotted with OD values (Y-axis) and standard protein concentrations (X-axis) to calculate the phosphorylated protein concentration in the samples. Protein phosphorylation level (%) = (concentration of phosphorylated protein in the sample × phosphorylation level of the standard protein (2.24%) × molecular weight of the phosphorylated protein)/[protein concentration of the sample × atomic weight of phosphorus(31)]. The relative expression levels of the experimental group were assessed based on the baseline set by the control group, followed by statistical analysis.

### RNA extraction, qRT-PCR, and RT-PCR analysis

Total RNA was extracted from mouse cerebellar tissues, mouse tumors, and Daoy cells utilizing TRIzon Reagent (CWBio, CW0580, Beijing, China). Following extraction, 1 μg of RNA from each sample was reverse transcribed into cDNA using HiScript III All-in-one RT SuperMix Perfect for qPCR (Vazyme, R333-01, Nanjing, China). Quantitative real-time PCR (qRT-PCR) was performed using the UltraSYBR One-Step RT-qPCR Kit (Cwbio, CW0659, Beijing, China), while reverse transcription PCR (RT-PCR) utilized the 2× Phanta Flash Master Mix (Vazyme, P520-01, Nanjing, China). Gene expression data was analyzed quantitatively by averaging triplicate measurement results in three independent experiments. PCR primers, synthesized by Sangon (Shanghai, China), were detailed in Table S1.

### Cell culture, transfection, and lentivirus infection

Daoy cell (ATCC® HTB186™) was purchased from the American Type Culture Collection (ATCC, Manassas, USA) and cultured in Eagle’s Minimum Essential Medium (EMEM, ATCC, 302003, Manassas, USA) enriched with 10% (v/v) fetal bovine serum (FBS, Gibco, 12484028, Waltham, USA), and a combination of 100 U/mL penicillin and 100 μg/mL streptomycin (Gibco, 15140-122, Waltham, USA). UW228 cell (BFN60808589) and ONS76 cell (BFN60808588) were purchased from the BFB biology (Shanghai, China) and cultured in Dulbecco Modified Eagle Medium (DMEM, Gibco, C11995500BT, Waltham, USA) enriched with 10% (v/v) fetal bovine serum (FBS, Gibco, 12484028, Waltham, USA), and a combination of 100 U/mL penicillin, 100 μg/mL streptomycin, and 5 ug/ml amphotericin B (BFB biology, BFNK012, Shanghai, China).

Short interfering RNAs (siRNAs) targeting *Eftud2*, *Kif3a*, *Gli2*, and scramble control siRNA were synthesized by Ribobio (Guangzhou, China). The siRNA sequences were GAGCAGACATTACCTGTTA for siEFTUD2, CTGCGTCAGTCTTTGATGA for siKIF3A, and GTCTGAGTGACACCAACCA for siGLI2. These siRNAs were transformed into cells using Lipofectamine™ RNAiMAX (Invitrogen, 13778150, Waltham, USA) according to the manufacturer’s instructions. For lentiviral transduction, Daoy cells were infected with lentiviruses carrying lentiviral vectors (pGC-FU-3FLAG-CBh-gcGFP-IRES-puromycin), pGC-FU-Kif3a^R^, and pGC-FU-Kif3a^ΔE10-11^ (Titer: 1 × 10^8^ TU/mL, 10 μL) in the culture medium, six hours post-siRNA transfection. Lentiviruses were purchased from GENEchem (Shanghai, China), and the accession number for the *Kif3a* cDNA used for the construction of lentivirus was XM_006532325.5.

### Cell proliferation detection

Cellular proliferation was assessed using CCK-8 assay, colony formation, and EdU incorporation assays. The CCK-8 assay was performed with the CCK-8 Cell Counting Kit (Vazyme, A311-01, Nanjing, China) following the manufacturer’s instructions. Specifically, 1000 cells in 100 μL of medium were seeded into each well of a 96-well plate, followed by the addition of 10 μL of CCK-8 solution. The plates were incubated at 37 °C for 2 h in a cell incubator under 5% CO2 atmosphere, and cellular proliferation was quantified by measuring absorbance at 450 nm using a spectrophotometer.

For the colony formation assay, 1000 cells were plated in each well of a 6-well plate and cultured under a humidified atmosphere with 5% CO2 at 37 °C for two weeks. The colonies were then fixed with 4% paraformaldehyde (Solarbio, P1110, Beijing, China) and stained with 0.1% crystal violet (Beyotime, C0121, Shanghai, China) for 15 min. Colonies were enumerated following rinsing with distilled water.

In the EdU incorporation assay, Daoy cells were incubated for 2 h in a medium supplemented with 10 μg/mL EdU (Ribobio, C00052, Guangzhou, China). Staining was performed using the Cell-Light EdU Apollo567 In vitro Kit (Ribobio, C10310-1, Guangzhou, China) according to the manufacturer’s protocol. Imaging was conducted using an Olympus FV-1200 confocal microscope, and the results were analyzed with Imaris software version 9.0.

### Luciferase reporter assay

The luciferase assay was performed according to a protocol previously established in the literature [37]. HEK293T cells, UW228 cells, and ONS76 cells were transfected with siRNAs targeting Eftud2, Kif3a, and scramble control for six hours. Following this, four distinct luciferase reporter plasmids—pGL4.2-8GBS, pGL3.0-Bcl2 promoter, pGL3.0-Bcl2 enhancer, and pGL4.2-Foxm1—were individually introduced into the siRNA-pre-treated cells. These plasmids were provided by Dr. Shiwen Luo at Nanchang University. Concurrently, the pRL-TK luciferase reporter was co-transfected as an internal control. Forty-eight hours after the second transfection, the cells were harvested, and luciferase activity was quantified using the Dual-Luciferase Reporter Assay System (Promega, E1910, Madison, USA), following the manufacturer’s guidelines.

### RNA-binding protein immunoprecipitation and sequencing

The RNA-binding protein immunoprecipitation (RIP) assay was performed utilizing the RIP Assay Kit (MBL, RN1001, Tokyo, Japan), adhering strictly to the manufacturer’s protocols. The RNA-protein complexes were immunoprecipitated using an anti-Eftud2 antibody (Novus, NBP2-92930, Saint Louis, USA). Following immunoprecipitation, total RNA was extracted from the complexes and sequenced by Annoroad Gene Technology (Beijing, China) using the Illumina Novaseq platform. The resulting data were analyzed by RT-PCR, employing primers detailed in Table S1.

### RNA-sequencing (RNA-seq), isoform sequencing (Iso-seq), and bioinformatics analysis

Total RNA was extracted from mouse cerebellum, tumor tissue, and Daoy cells using TRIzon Reagent (CWbio, CW0580, Beijing, China). The quality and quantity of RNA were assessed using a NanoDrop spectrophotometer (Thermo Fisher Scientific, Waltham, USA). Subsequent high-throughput sequencing was conducted by Majorbio (Shanghai, China) on the Illumina Novaseq 6000 platform. In the P30 cerebellar tissue RNA-seq analysis, the sequencing depth for the *SmoM2*^*fl/+*^ group is 10.24×, 10.86×, and 11.33×, while the sequencing depth for the *SmoM2*^*Atoh1*^ group is 10.48×, 10.06×, and 10.13×, meeting the requirements for differential gene expression and alternative splicing analysis. Specifically, the read counts for cerebellar samples of the *SmoM2*^*fl/+*^ group were 49,778,738; 54,478,356; and 69,136,908. The read counts for tumor samples of the *SmoM2*^*Atoh1*^ group were 55,533,738; 53,354,666; and 54,547,890 (Table S3). In the siEFTUD2-knockdown Daoy cell RNA-seq analysis, the sequencing depth for the Scramble group is 4.72×, 5.21×, and 5.12×, while the sequencing depth for the siEFTUD2 group is 5.23×, 5.32×, and 4.94×, meeting the requirements for differential gene expression and alternative splicing analysis. Specifically, the read counts for cell samples of the Scramble group were 44,056,696; 54,048,076; and 53,208,302. The read counts for cell samples of the siEFTUD2 group were 50,294,052; 55,813,366; and 44,278,120 (Table S3).

Additionally, Iso-seq libraries from the mouse cerebellum and tumor tissue were prepared using the PacBio Sequel platform (Majorbio, Shanghai, China). Data analysis was executed using Cuffdiff and the statistical software R [78]. Read counts for cerebellar samples from the *SmoM2*^*fl/+*^ group were 17,097,585; 17,851,191; and 15,367,504, while read counts for tumor samples from the *SmoM2*^*Atoh1*^ group were 11,545,795; 17,800,266; and 15,325,381.

For the splicing analysis conducted with Iso-Seq, alternative splice events were identified using the rMATS program (http://rnaseq-mats.sourceforge.net/index.html). We considered isoforms that matched the reference or contained novel splice junctions, assessing splicing variations such as exon inclusion/exclusion, alternative 5’/3’ splice sites, and intron retention. Pathway analysis was performed utilizing the Kyoto Encyclopedia of Genes and Genomes (KEGG) database, accessible at https://www.genome.jp/kegg/pathway.html, and DAVID bioinformatics resources, available at https://david.ncifcrf.gov/.

### Human medulloblastoma splicing event quantification

To evaluate alternative splicing events, we firstly downloaded raw RNA sequencing data of childhood medulloblastoma tumors and normal cerebellum tissue from the Sequence Read Archive (SRA, accession number: SRR26129072-SRR26129234). We removed reads containing ploy-N and low-quality reads using Cutadapt (version 1.15). Clean reads were mapped to human reference genome from Ensembl release 106 (GRCh38) using HISAT2 (version 2.2.1). Next, bam files were indexed with SAM tools (version 1.3.1) as the input files for rMATS software (replicate Multivariate Analysis of Transcript Splicing; version 4.1.2). rMATS assesses individual splicing events from five different categories: skipped exons, retained intron, alternative 5′ splicing site, alternative 3′ splicing site, and mutually exclusive exons. Both reads mapped to the splice junctions and those mapped to the exon body were used to identify each differentially spliced event. FDR cutoff <0.01 was used to define significantly differential alternative splicing events. HTSeq (version 0.12.4) was used to count the number of reads mapped in each annotated gene based on the mapping results. R package DESeq2 (version 1.26.0) was used to calculate DEGs with the cutoff values of FDR < 0.05 and |Log2 (fold change) | > 1.

### Statistical analysis

Each experimental trial was replicated at least six times. Data are presented as the mean ± standard deviation (SD). Statistical data from different experiments were analyzed according to their corresponding and standard biological statistical methods (Table S6). Survival rates were estimated using the Kaplan–Meier method, and differences were evaluated using the log-rank test. A p-value of less than 0.05 was considered statistically significant. For other statistical analysis, unpaired *t*-test, one-way or two-way ANOVA, and multiple comparison tests were used and analyzed using GraphPad Prism 9.5 (GraphPad, San Diego, CA, USA).

## Supplementary information

Atoh1-SmoM2-Eftud2-SI-CDDiff-R2-0125

Original Western blot images-20250125

Cell lines identification and detection-Merged

aj-checklist-Finished

## Data Availability

All original sequencing data in this study have been deposited in the National Center for Biotechnology Information Sequence Read Achieve (accession no. PRJNA1113185 and PRJNA1113204).
