# Supplementary material for: Alternative spliceosomal protein Eftud2 mediated Kif3a exon skipping promotes SHH-subgroup medulloblastoma progression
Source: Cell Death Differ. 2025 Apr 24;32(10):1930–45. doi: 10.1038/s41418-025-01512-9 (PMC12501224; doi:10.1038/s41418-025-01512-9)
Supplement: Supplementary file 1 — Atoh1-SmoM2-Eftud2-SI-CDDiff-R2-0125 [file 41418_2025_1512_MOESM1_ESM.pdf]

Supplementary information

Alternative spliceosomal protein Eftud2 mediated *Kif3a* exon kipping promotes SHH subgroup medulloblastoma progression

Running title: Eftud2 promotes medulloblastoma progression

Ying Li[1,2,#], Liping Chen[1,#], Saisai Xue[1,#], Zhihong Song[1], Heli Liu[1,3], Hao Li[1], Wei Shen[1], Chen Zhang[2,*], Haitao Wu[1,4,5,*]

[1]Department of Neurobiology, Beijing Institute of Basic Medical Sciences, 100850 Beijing, China

[2]School of Basic Medical Sciences, Beijing Key Laboratory of Neural Regeneration and Repair, Advanced Innovation Center for Human Brain Protection, Capital Medical University, 100069 Beijing, China.

[3]Institute of Neuroscience, Hengyang Medical College, University of South China, Hengyang, 421001 Hunan, China.

[4]Key Laboratory of Neuroregeneration, Co-innovation Center of Neuroregeneration, Nantong University, 226019 Nantong, China

[5]Chinese Institute for Brain Research, 102206 Beijing, China

[#]Y. L., L. C., and S. X. contributed equally to this work

*To whom correspondence may be addressed. E-mail: wuht@bmi.ac.cn or czhang@ccmu.edu.cn

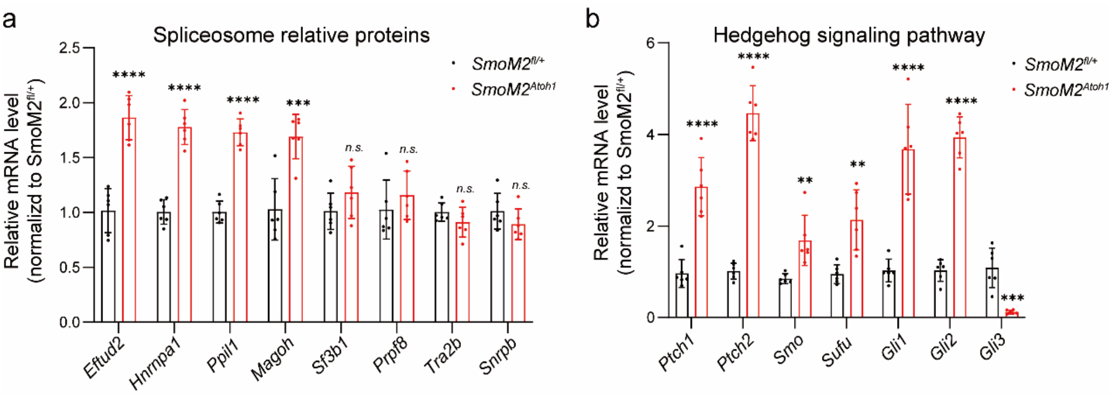

Fig. S1 Screening of essential tumor regulatory genes in *SmoM2^{Atoh1}* mice. a, b Quantitative PCR (qPCR) validation of key factors within the Spliceosome **a** and SHH signaling pathway **b** (n = 6 mice, 3 males, 3 females; unpaired *t*-test). Data are presented as mean ± SD (bar plots). $^{**}p < 0.01$, $^{***}p < 0.001$, $^{****}p < 0.0001$, *n.s.*, no significant.

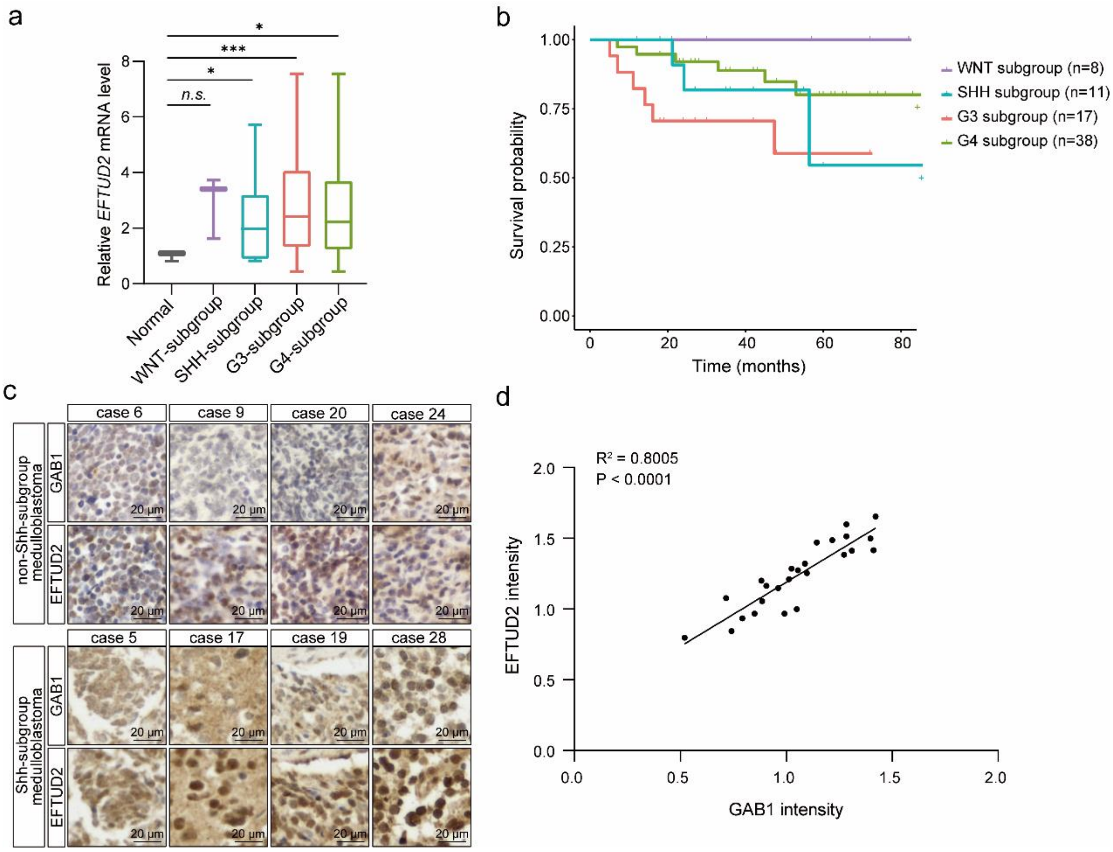

Fig. S2 Positive correlation between EFTUD2 expression and SHH-subgroup medulloblastoma. a Relative *EFTUD2* mRNA levels in cerebellar tissue from normal individuals and tumor tissues from WNT, SHH, G3 and G4 subgroup medulloblastoma. (one-way ANOVA and Bonferroni's multiple comparisons test). **b** Kaplan-Meier survival curves for patients with WNT, SHH, G3 and G4 subgroup medulloblastoma (Gehan-Breslow-Wilcoxon test). **c** Representative immunohistochemistry images of non-SHH (upper panel) and SHH-subgroup (lower panel) clinical medulloblastoma samples, stained with indicated antibodies. Scale bar: 20 µm. **d** Linear regression analysis demonstrating a positive correlation between EFTUD2 and GAB1 expression in SHH-subgroup medulloblastoma. Immunohistochemistry staining intensity for each antibody was quantitatively analyzed and compared across 25 medulloblastoma sample (n = 25, Pearson product-moment correlation coefficient analysis). Data are presented as the mean ± SD (bar plots). $^{*}p < 0.05$, $^{***}p < 0.001$, *n.s.*, no significant.

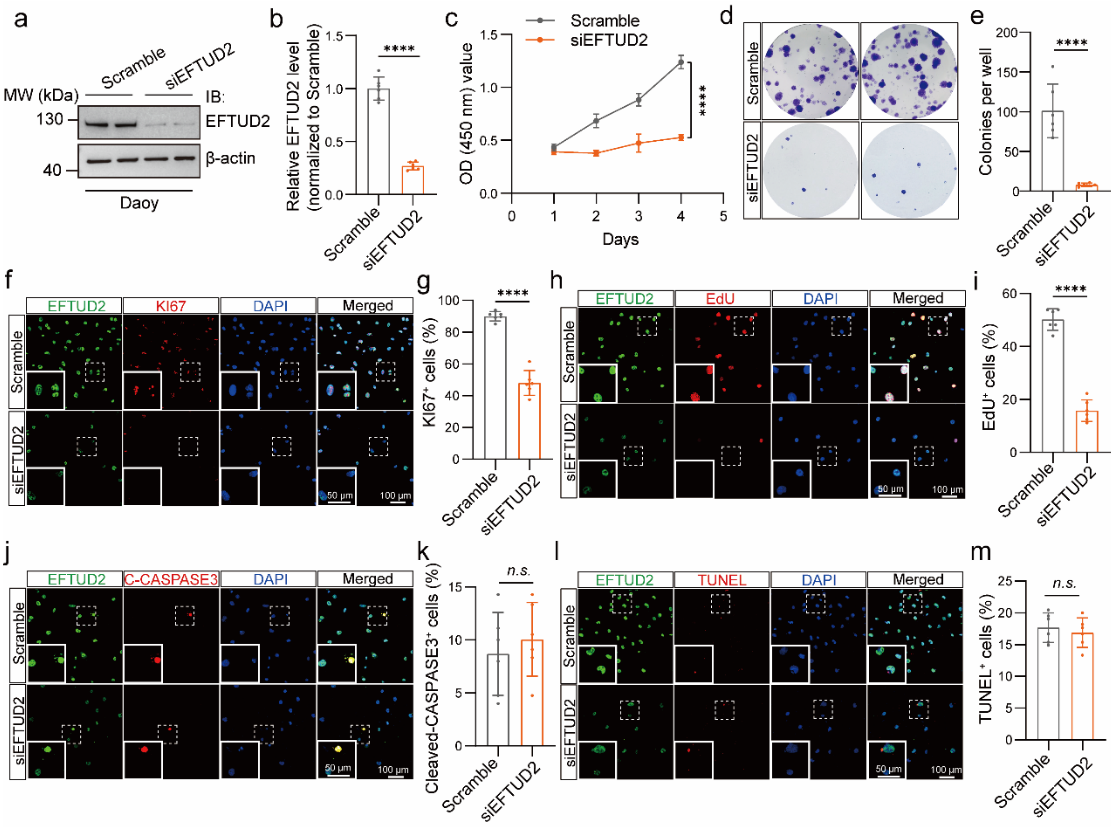

Fig. S3 EFTUD2 promotes malignant proliferation of Daoy cells. a, b Western blot validation **a** and quantification **b** of EFTUD2 expression following *EFTUD2* silencing in Daoy cells (n = 6, unpaired *t*-test). **c-e** Effect of *EFTUD2* silencing on cellular viability in Daoy cells, assessed by CCK-8 assays **c** and colony formation assays **d**, **e** (n = 6, two-way ANOVA and Šídák's multiple comparisons test for CCK-8 analysis, unpaired *t*-test for colony formation analysis). **f-i** Immunofluorescence staining **f**, **h** and quantification **g**, **i** showing a significant reduction in the percentage of $KI67^{+}$ and EdU^{+} cells in the siEFTUD2 group compared to that in the Scramble group (n = 6, unpaired *t*-test). Scale bars = 20 μm and 100 μm, respectively. **j-m** Immunofluorescence staining **j**, **l** and quantification **k**, **m** showing no significant change in the percentage of Cleaved-$CASPASE3^{+}$ and $TUNEL^{+}$ cells in Daoy cells in the siEFTUD2 group (C-CASPASE3, Cleaved-CASPASE3, n=6, unpaired *t*-test). Scale bars = 20 μm and 100 μm, respectively. Data are presented as the mean ± SD (bar plots). $^{****}p < 0.0001$, *n.s.*, no significant.

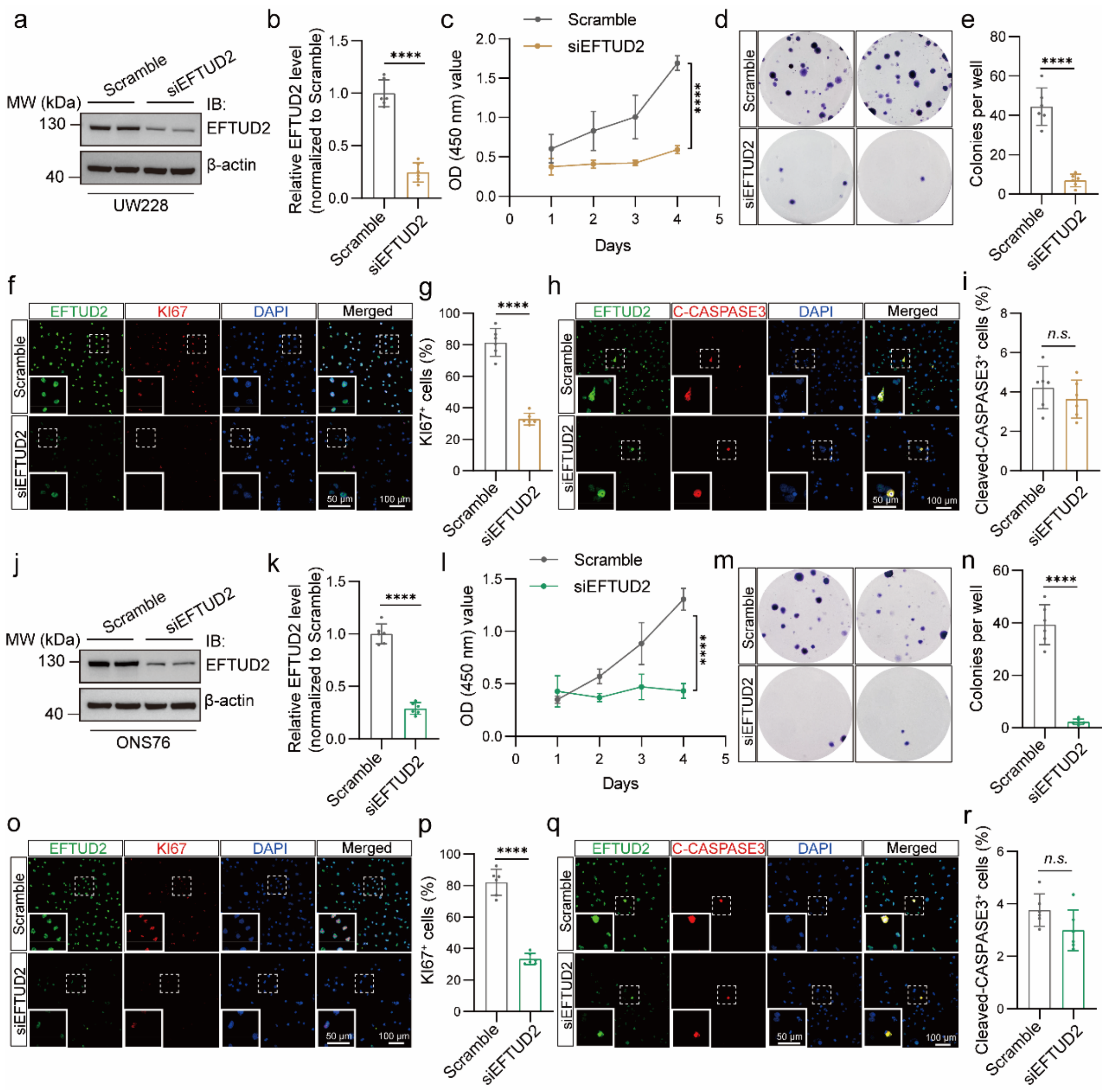

Fig. S4 EFTUD2 promotes malignant proliferation of human medulloblastoma cell lines UW228 and ONS76. a, b Western blot validation **a** and quantification **b** of EFTUD2 following *EFTUD2* silencing in UW228 cells (n = 6, unpaired *t*-test). **c-e** Effect of *EFTUD2* silencing on cellular viability in UW228 cells, assessed by CCK-8 assays **c** and colony formation assays **d**, **e** (n = 6, two-way ANOVA and Šídák's multiple comparisons test for CCK-8 assays analysis, unpaired *t*-test for colony formation assays analysis). **f, g** Immunofluorescence staining **f** and quantification **g** showed that the percentage of $KI67^+$ cells in UW228 cells in the siEFTUD2 group was significantly reduced (n = 6, unpaired *t*-test). Scale bars = 20 μm and 100 μm, respectively. **h, i** Immunofluorescence staining **h** and quantification **i** showing no significant change in the percentage of $Cleaved\text{-}CASPASE3^+$ cells in UW228 cells following *EFTUD2* silencing (C-CASPASE3, Cleaved-CASPASE3, n=6, unpaired *t*-test). Scale bars = 20 μm and 100 μm. **j, k** Western blot validation **j** and quantification **k** of EFTUD2 expression following *EFTUD2* silencing in ONS76 cells (n = 6, unpaired *t*-test). **l-n** Effect of *EFTUD2* silencing on cellular viability in ONS76 cells, assessed by CCK-8 assays **l** and colony formation assays **m**, **n** (n = 6, two-way ANOVA and Šídák's multiple comparisons test for CCK-8 analysis, unpaired *t*-test for colony formation analysis). **o,**

p Immunofluorescence staining **o** and quantification **p** showing a significant reduction in the percentage of $KI67^{+}$ cells in the siEFTUD2 group compared to that in the Scramble group (n = 6, unpaired *t*-test). Scale bars = 20 µm and 100 µm. **q, r** Immunofluorescence staining **q** and quantification **r** showing no significant change in the percentage of Cleaved-$CASPASE3^{+}$ cells in ONS76 cells following *EFTUD2* silencing (C-CASPASE3, Cleaved-CASPASE3, n=6, unpaired *t*-test). Scale bars = 20 µm and 100 µm. Data are presented as the mean ± SD (bar plots). $^{****}p < 0.0001$, *n.s.*, no significant.

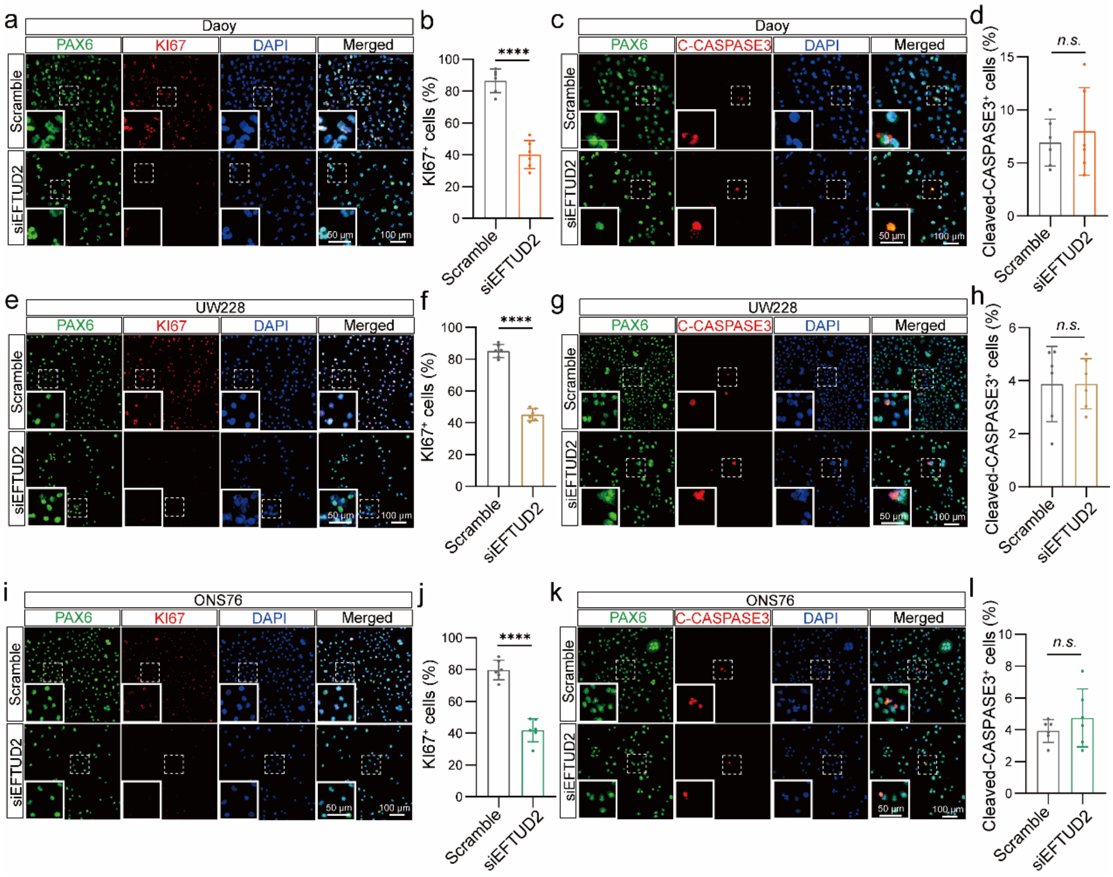

Fig. S5 EFTUD2 promotes malignant proliferation in GNP-like Daoy, UW228, and ONS76 Cells. a, b Immunofluorescence staining **a** and quantitative analysis **b** showing a significant reduction in the percentage of $KI67^+$ cells among $PAX6^+$ Daoy cells following *EFTUD2* silencing (n = 6, unpaired *t*-test). Scale bars: 20 μm and 100 μm. **c, d** Immunofluorescence staining **c** and quantitative analysis **d** showing no significant change in the percentage of Cleaved-$CASPASE3^+$ (C- CASPASE3) $PAX6^+$ Daoy cells following *EFTUD2* silencing (n = 6, unpaired *t*-test). **e, f** Immunofluorescence staining **e** and quantitative analysis **f** demonstrating a significant decrease in the proportion of $KI67^+$ cells among $PAX6^+$ UW228 cells following *EFTUD2* silencing (n = 6, unpaired *t*-test). Scale bars: 20 μm and 100 μm. **g, h** Immunofluorescence staining **g** and quantitative analysis **h** showing no significant alteration in the percentage of Cleaved-$CASPASE3^+$ cells in $PAX6^+$ UW228 cells after EFTUD2 knockdown (n = 6, unpaired *t*-test). **i, j** Immunofluorescence staining **i** and quantitative analysis **j** revealing a significant reduction in the percentage of $KI67^+$ cells in $PAX6^+$ ONS76 cells following *EFTUD2* silencing (n = 6, unpaired *t*-test). Scale bars: 20 μm and 100 μm. **k, l** Immunofluorescence staining **k** and quantitative analysis **l** showing no significant change in Cleaved-$CASPASE3^+$ levels in $PAX6^+$ ONS76 cells following *EFTUD2* silencing (n = 6, unpaired *t*-test). Scale bars: 20 μm and 100 μm. Data are presented as mean ± SD in bar plots. $^{****}p < 0.0001$; n.s., not significant.

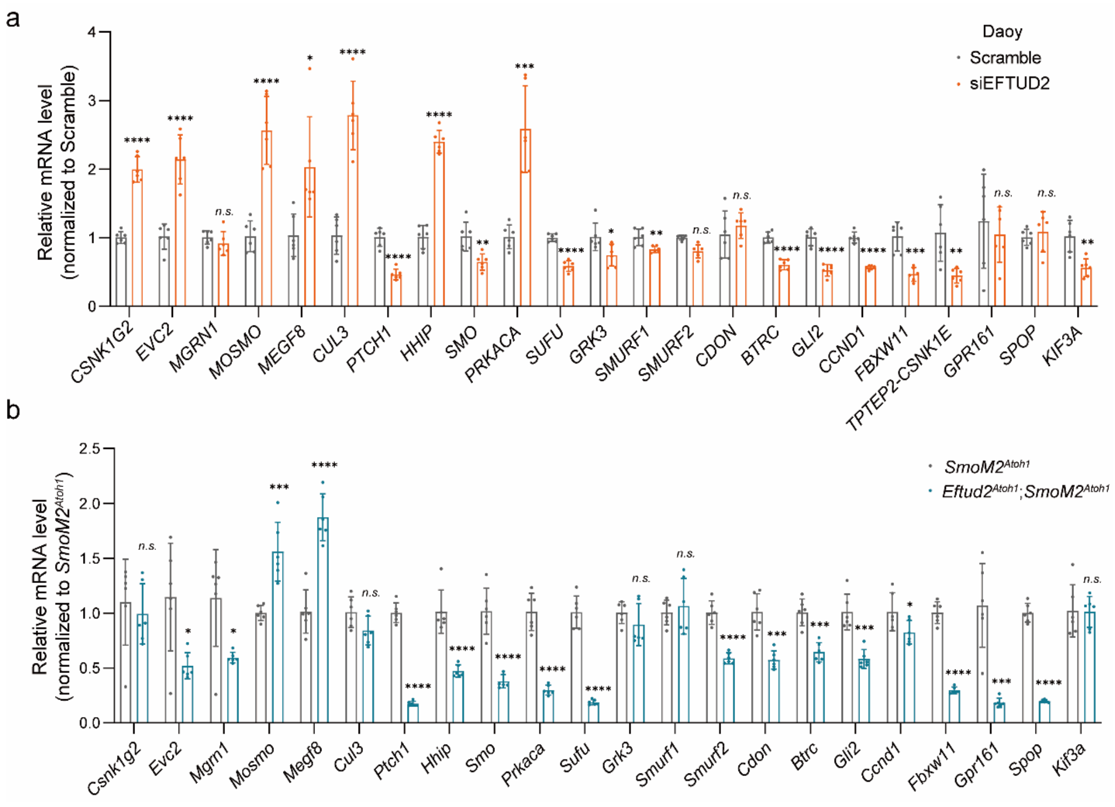

Fig. S6 RNA expression of SHH signaling pathway genes in EFTUD2-regulated Daoy cells and medulloblastoma mice. a, b qPCR validation of key SHH pathway factors in Daoy cells **a** treated with Scramble or EFTUD2 siRNA, and in medulloblastoma samples from *SmoM2*^{Atoh1} and *Eftud2*^{Atoh1}; *SmoM2*^{Atoh1} mice **b** (n = 6 mice, 3 males, 3 females; unpaired *t*-test). Data are presented as mean ± SD (bar plots). $^{*}p < 0.05$, $^{**}p < 0.01$, $^{***}p < 0.001$, $^{****}p < 0.0001$, *n.s.*, no significant.

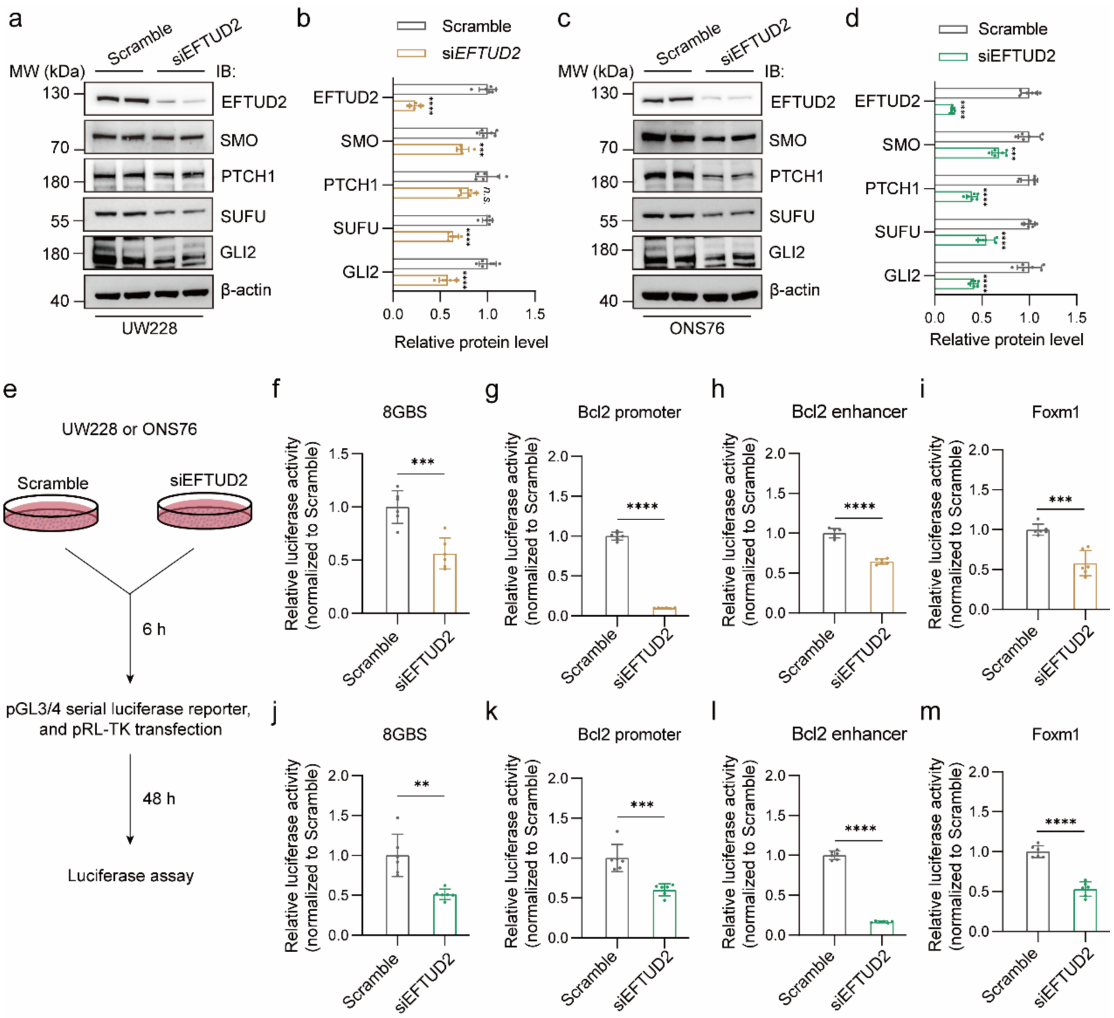

Fig. S7 Knockdown of EFTUD2 inhibits the activation of SHH pathway and the expression and transcriptional activity of GLI2 in UW228 and ONS76 cells. a-d Western blot validation **a**, **c** and quantification **b**, **d** of essential SHH signaling pathway factors in Scramble and siEFTUD2-treated UW228 **a**, **b** and ONS76 **c**, **d** cells, respectively (n = 6, unpaired *t*-test). **e-m** Schematic of luciferase assay **e** and quantification of transcriptional activity of four different GLI2 downstream effectors-8GBS, Bcl2 promoter, Bcl2 enhancer, and Foxm1-in UW228 **f-i** and ONS76 **j-m** cells (n = 6, unpaired *t*-test). Data are presented as mean ± SD (bar plots). $^{**}p < 0.01$, $^{***}p < 0.001$, $^{****}p < 0.0001$.

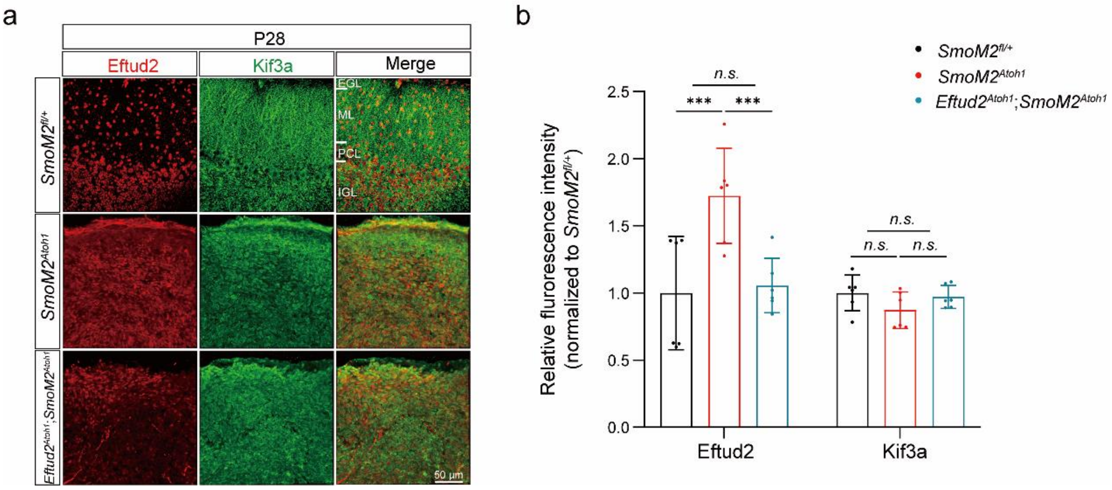

Fig. S8 Eftud2 does not alter the protein level of Kif3a in SHH-subgroup medulloblastoma. a Immunofluorescent co-staining with anti-Eftud2 and anti-Kif3a in sagittal section of the cerebellar vermis from P28 *SmoM2*$^{fl/+}$, *SmoM2*^{Atoh1} and *Eftud2*^{Atoh1}*; SmoM2*^{Atoh1} mice. Scale bars = 50 μm. **b** Quantification of fluorescence intensity of Eftud2 and Kif3a in the cerebellum of *SmoM2*$^{fl/+}$ mice, *SmoM2*^{Atoh1} mice and *Eftud2*^{Atoh1}*; SmoM2*^{Atoh1} mice at P28 (n = 6 mice, 3 males, 3 females; one-way ANOVA and Bonferroni's multiple comparisons test). Molecular Layer (ML), the Purkinje Layer (PL), and the Internal Granule Layer (IGL). Data are presented as mean ± SD (bar plots). $^{***}p < 0.001$, *n.s.*, no significant.

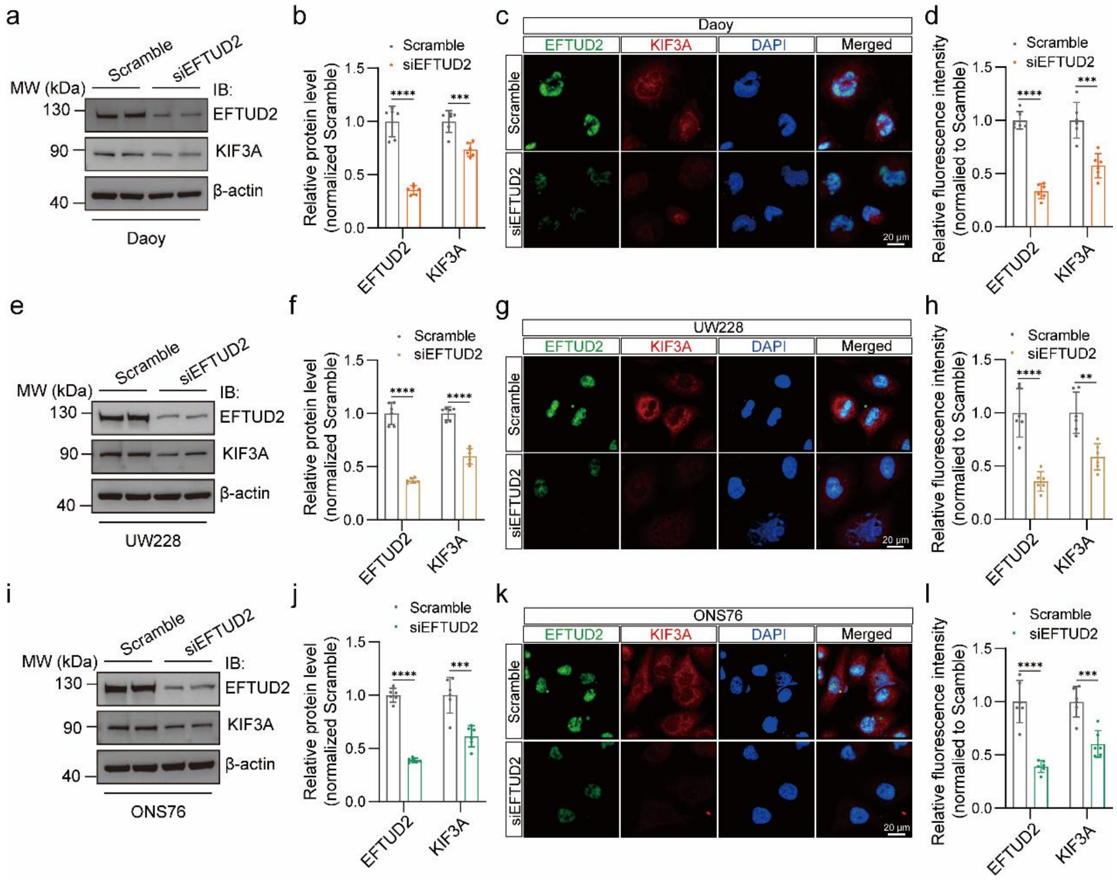

Fig. S9 EFTUD2 maintains the protein level of KIF3A in human medulloblastoma cell lines. a, b Western blot validation **a** and quantification **b** of EFTUD2 and KIF3A following *EFTUD2* silencing in Daoy cells (n = 6, unpaired *t*-test). **c, d** Immunofluorescence staining **c** and quantification **d** showing a significant reduction in the fluorescence intensity of EFTUD2 and KIF3A in Daoy cells after EFTUD2 knockdown (n = 6, unpaired *t*-test). Scale bars = 20 μm. **e, f** Western blot validation **e** and quantification **f** of EFTUD2 and KIF3A following *EFTUD2* silencing in UW228 cells (n = 6, unpaired *t*-test). **c-d** Immunofluorescence staining **c** and quantification **d** showing a significant reduction in the fluorescence intensity of EFTUD2 and KIF3A in UW228 cells in the siEFTUD2 group was significantly reduced after EFTUD2 knockdown (n = 6, unpaired *t*-test). Scale bars = 20 μm. **i, j** Western blot validation **i** and quantification **j** of EFTUD2 and KIF3A following EFTUD2 silencing in ONS76 cells (n = 6, unpaired *t*-test). **k-i** Immunofluorescence staining **k** and quantification **i** showing a significant reduction in the fluorescence intensity of EFTUD2 and KIF3A in ONS76 cells after EFTUD2 knockdown (n = 6, unpaired *t*-test). Scale bars = 20 μm. Data are presented as mean ± SD (bar plots). $^{**}p < 0.01$, $^{***}p < 0.001$, $^{****}p < 0.0001$.

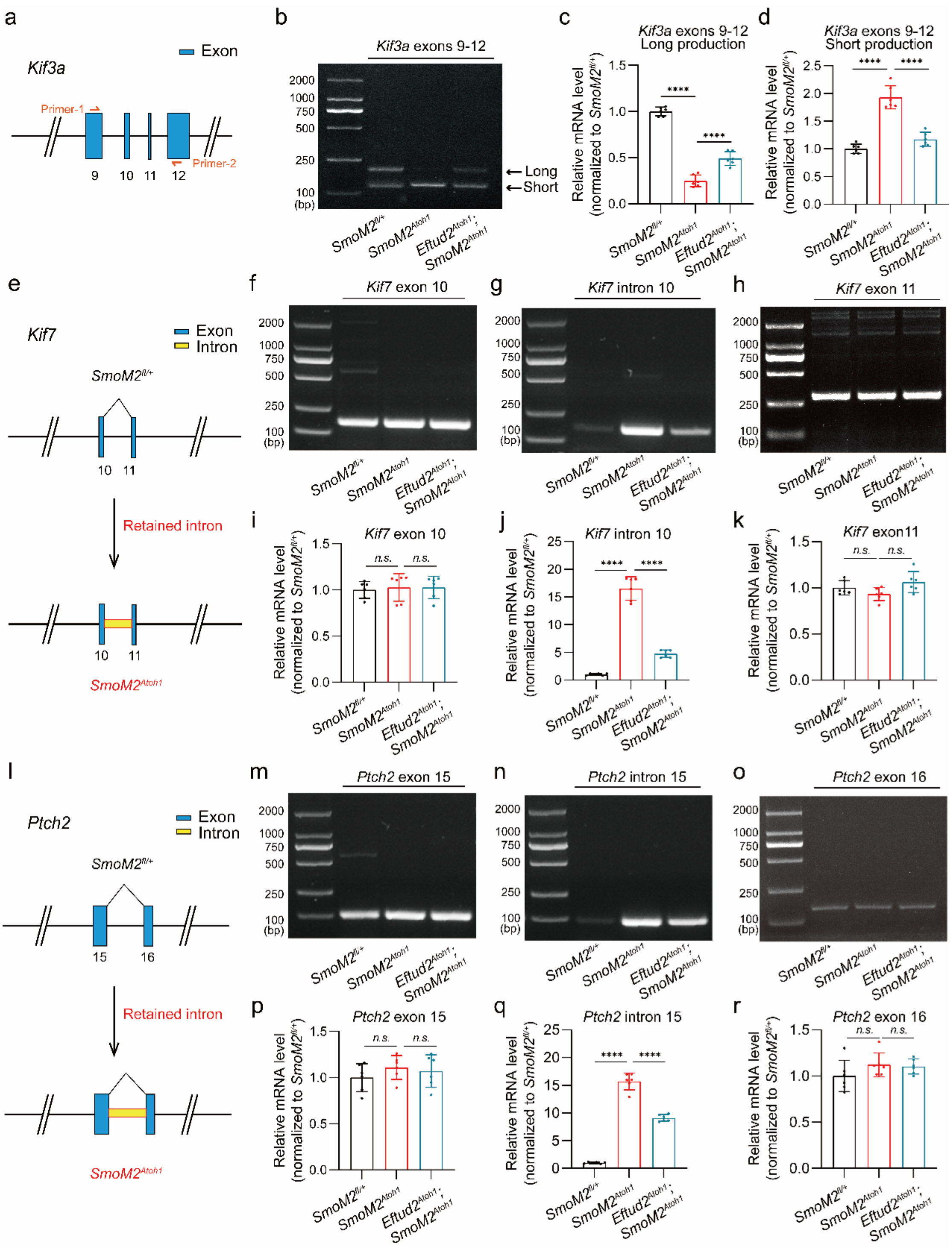

Fig. S10 Eftud2 regulates RNA alternative splicing of genes in the Hedgehog signaling pathway in SHH-subgroup medulloblastoma. a *Kif3a* exons 10-11 skipping in *SmoM2*^{Atoh1} mouse tumors. **b-d** RT-PCR validation **b** and quantification **c-d** of *Kif7* short exons 9-12 **c** and long exons 9-12 **d** in the cerebellum of *SmoM2*$^{fl/+}$ and *SmoM2*^{Atoh1} mouse tumors (n = 6 mice, 3 males, 3 females; one-way ANOVA and Bonferroni's multiple comparisons test). **e** *Kif7* intron 10 retention in *SmoM2*^{Atoh1} mouse tumors. **f-k** RT-PCR validation **f-h** and quantification **i-k** of *Kif7* exon 10 **f**, **i**, intron 10 **g**, **j** and exon 11 **h**, **k** in the cerebellum of *SmoM2*$^{fl/+}$ and *SmoM2*^{Atoh1} mouse tumors (n

= 6 mice, 3 males, 3 females; one-way ANOVA and Bonferroni's multiple comparisons test). **l** *Ptch2* intron 15 retention in *SmoM2*^{Atoh1} mouse tumors. **m-r** RT-PCR validation **m-o** and quantification **p-r** of *Ptch2* exon 15 **m**, **p**, intron 15 **n**, **q** and exon 16 **o**, **r** in the cerebellum of *SmoM2*$^{fl/+}$ and *SmoM2*^{Atoh1} mouse tumors (n = 6, mice, 3 males, 3 females; one-way ANOVA and Bonferroni's multiple comparisons test). Data are presented as mean ± SD (bar plots). $^{****}p < 0.0001$, *n.s.*, no significant.

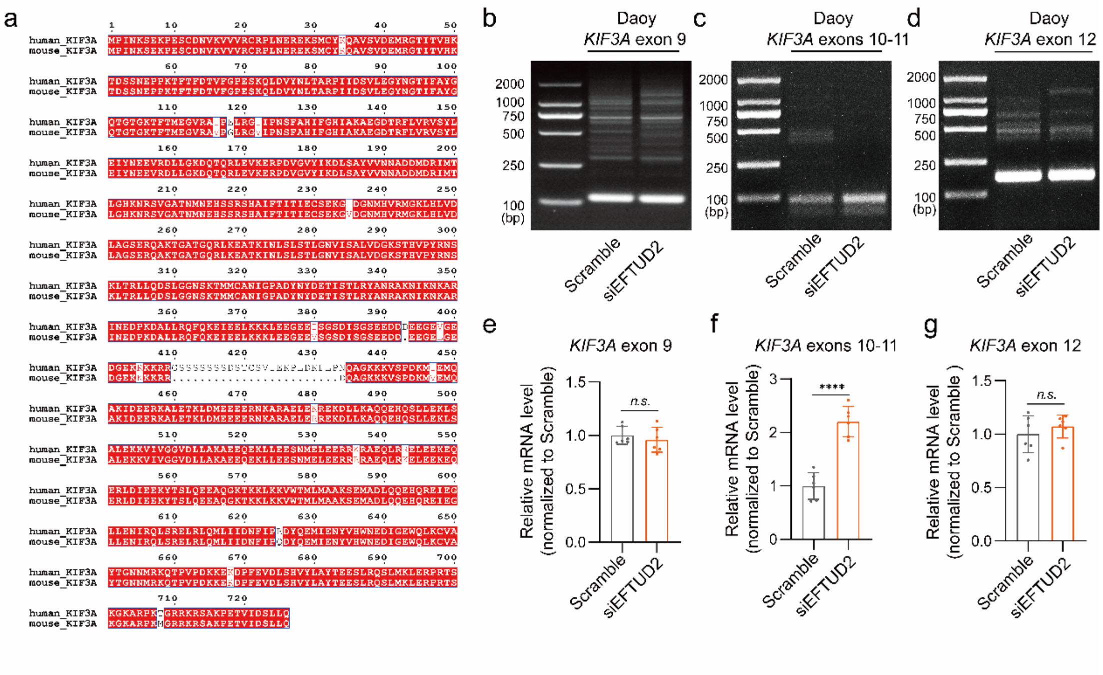

Fig. S11 Knockdown of EFTUD2 induces skipping of *KIF3A* exon 10 and 11 in Daoy cells. a Cross-species sequence alignment of KIF3A showing high conservation of the protein between human and mouse**. b-g** RT-PCR validation **b-d** and quantification **e-g** of *KIF3A* exon 9 **b**, **e**, exons 10-11 **c**, **f** and exon 12 **d**, **g** in Daoy cell treated with Scramble siRNA or siRNA targeting EFTUD2 (n = 6, unpaired *t*-test). Data are presented as mean ± SD (bar plots). $^{****}p < 0.0001$, *n.s.*, no significant.

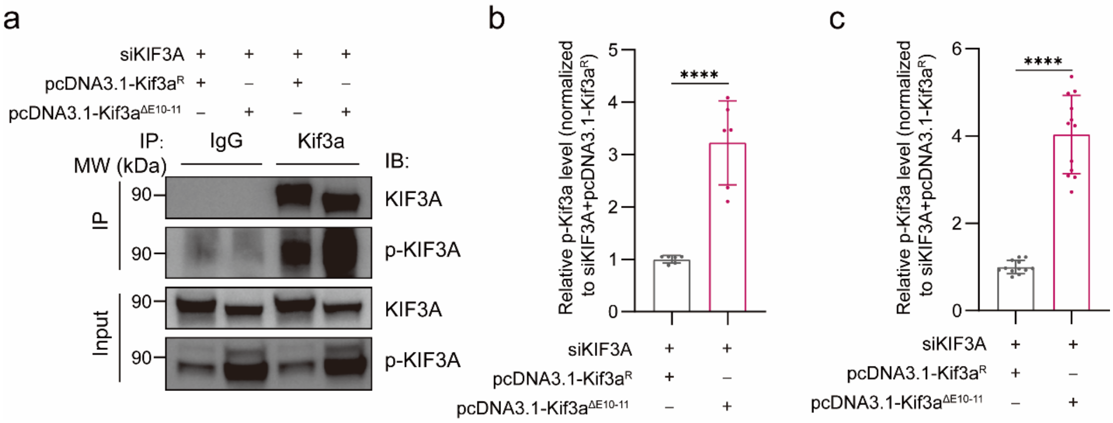

Fig. S12 Skipping of exons 10-11 in *Kif3a* enhances its phosphorylation level. a, b Representative immunoblot **a** and quantification **b** of phosphorylation levels in immunoprecipitated Kif3a^{R} and Kif3a$^{\Delta E10\text{-}11}$. HEK293T cells were transfected for 48 hours with either Kif3a^{R} or Kif3a$^{\Delta E10\text{-}11}$ construct (n = 6, unpaired Student's t-test). **c** Detection of phosphorylation levels in Kif3a^{R} and Kif3a$^{\Delta E10\text{-}11}$ construct (n = 12, unpaired *t*-test). Data are presented as mean ± SD (bar plots). $^{****}p < 0.0001$.

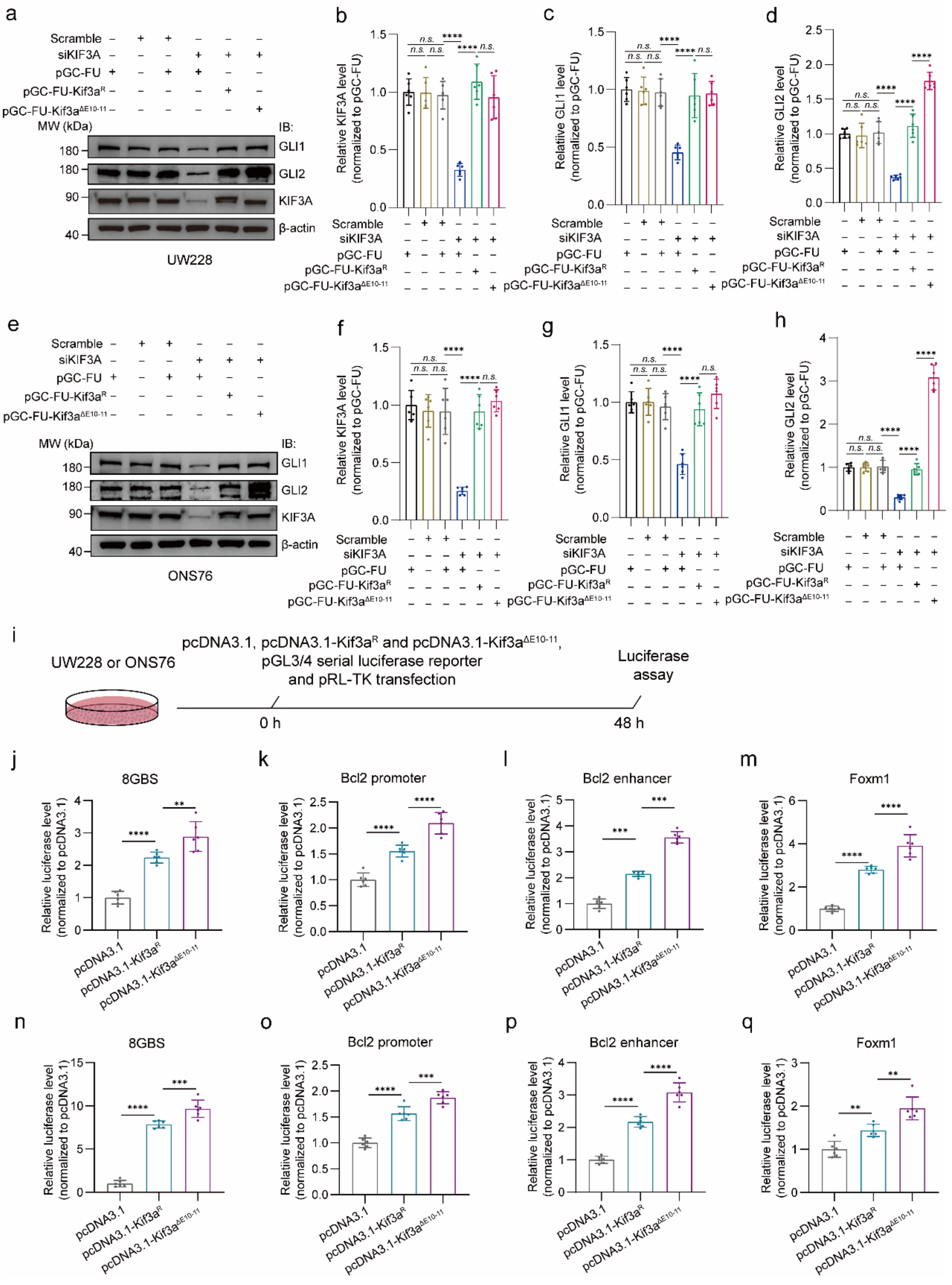

Fig. S13 Skipping of exons 10-11 in *Kif3a* enhances the transcriptional activity of GLI2 in UW228 and ONS76 cells. a-h Western blot validation **a**, **e** and quantification **b-d, f-h** of GLI1, GLI2 and KIF3A in in various treatment groups: pGC-FU control, Scramble siRNA control, pGC-FU + Scramble siRNA control, pGC-FU + siKIF3A, siKIF3A + Kif3a^{R} and siKIF3A + Kif3a$^{\Delta E10\text{-}11}$ in UW228 **b-d** and ONS76 **f-h** cells ($n = 6$, one-way ANOVA and Bonferroni's multiple comparisons test). **i-q** Schematic of luciferase assay **i** and quantification **j-q** of transcriptional activity of four different GLI2 downstream effectors (8GBS, Bcl2 promoter, Bcl2 enhancer, and Foxm1) in UW228 **j-**

m and ONS76 **n-q** cells (n = 6, one-way ANOVA and Bonferroni's multiple comparisons test). Data are presented as the mean ± SD (bar plots). $^{**}p < 0.01$, $^{***}p < 0.001$, $^{****}p < 0.0001$, *n.s.*, no significant.

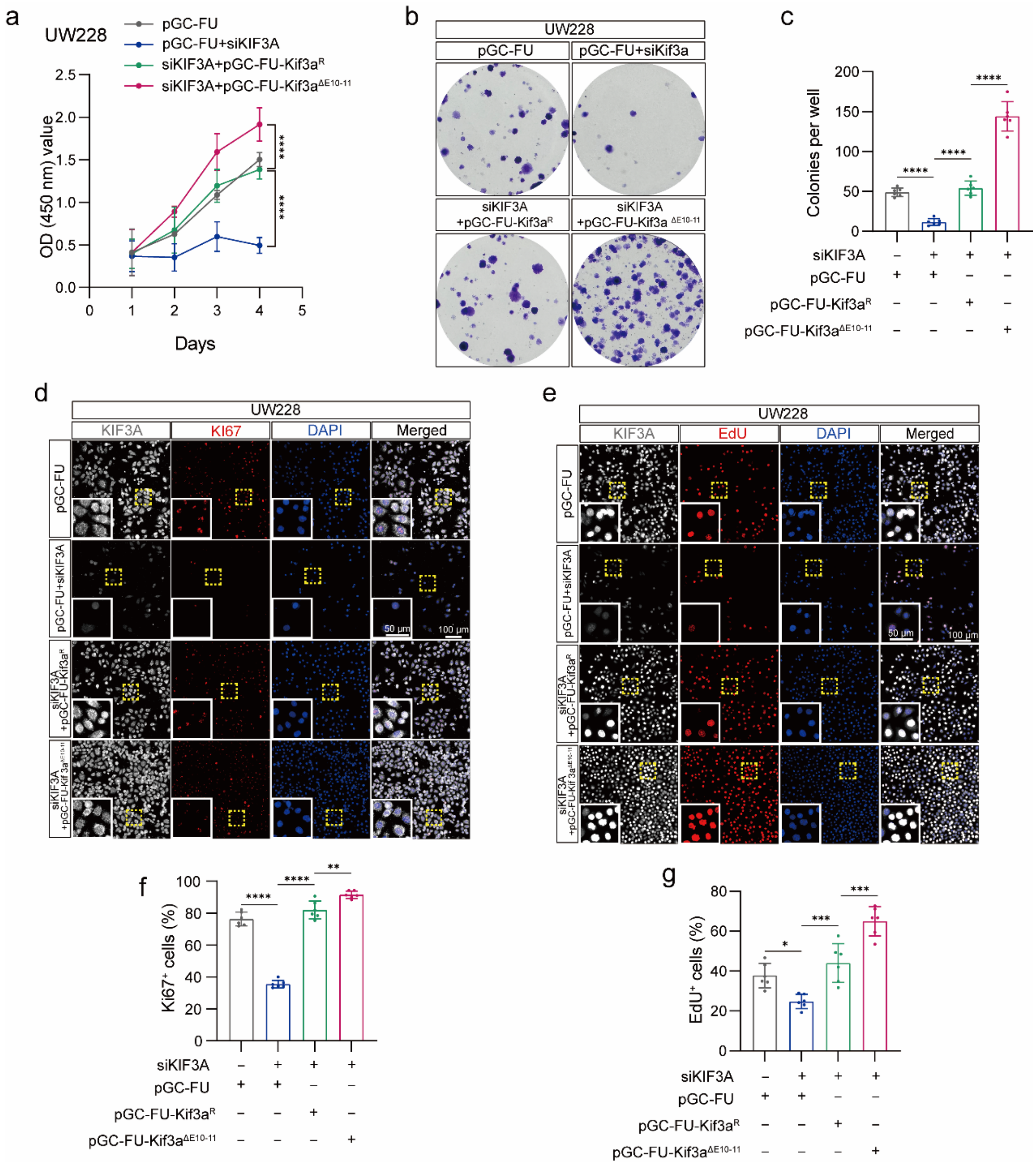

Fig. S14 Skipping of exons 10-11 in *Kif3a* promotes the proliferation of UW228 cells. a-c The impact of Kif3a and Kif3a$^{\Delta E10\text{-}11}$ on the viability of UW228 cells was assessed using CCK-8 assays **a** and colony formation assays **b, c** (n = 6, two-way ANOVA and Šidák's multiple comparisons test for CCK-8 assays, one-way ANOVA and Bonferroni's multiple comparisons test for colony formation assays analysis). **d, f** Immunofluorescent co-staining **d** with anti-KIF3A and anti-KI67 and quantification **f** in pGC-FU control, pGC-FU + siKIF3A, siKIF3A + Kif3a^{R} and siKIF3A + Kif3a$^{\Delta E10\text{-}11}$ treated UW228 cells (one-way ANOVA and Bonferroni's multiple comparisons test). Scale bars = 50 μm and 100 μm. **e, g** EdU incorporation assays **e** and quantification **g** to identify UW228 cells in S phase under the same treatment conditions (one-way ANOVA and Bonferroni's multiple comparisons test). Scale bars = 50 μm and 100 μm. Data are presented as mean ± SD (bar plots). $^{*}p < 0.05$, $^{**}p < 0.01$, $^{***}p < 0.001$, $^{****}p < 0.0001$.

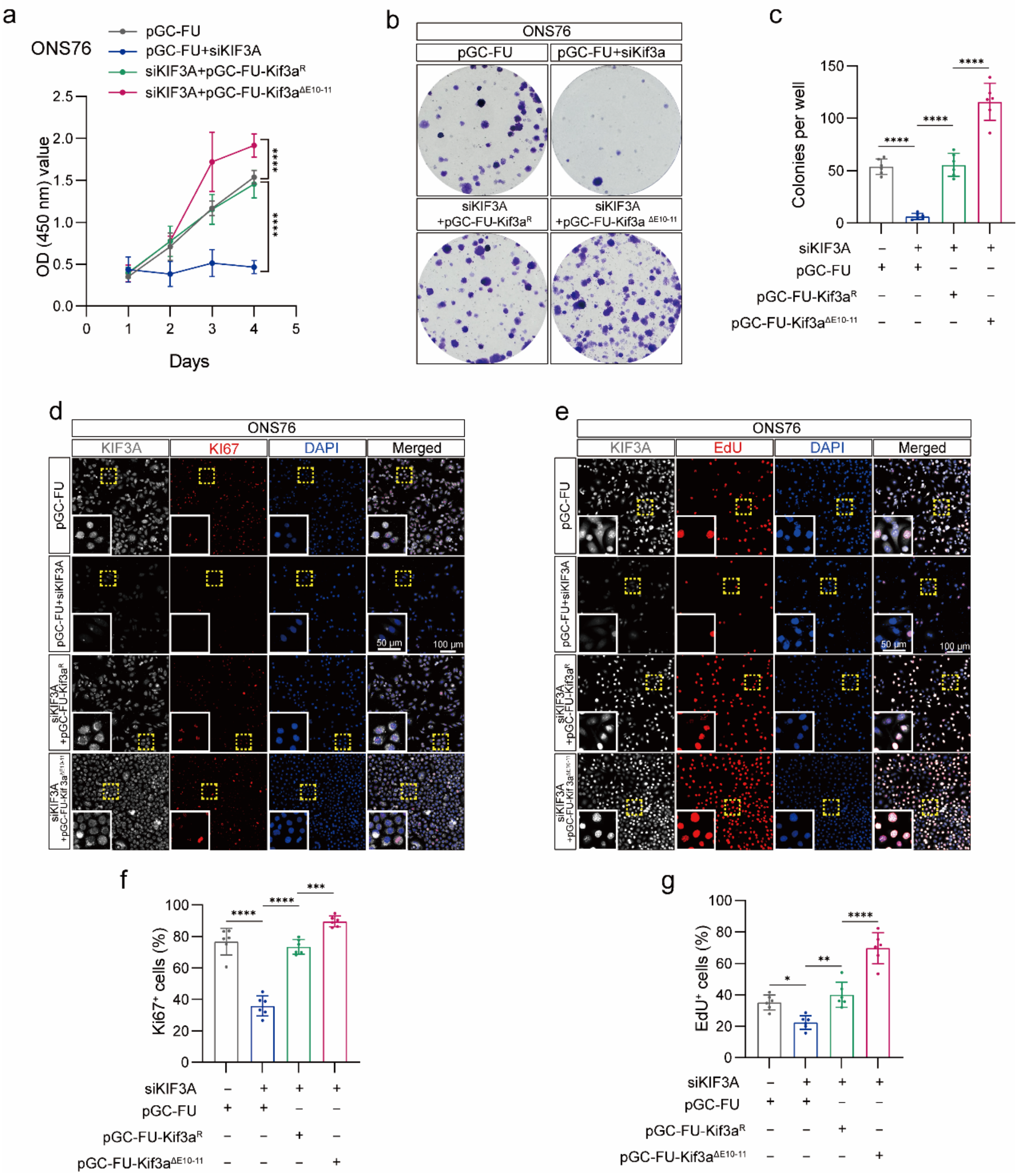

Fig. S15 Skipping of exons 10-11 in *Kif3a* promotes the proliferation of ONS76 cells. a-c The effect of Kif3a and Kif3a$^{\Delta E10\text{-}11}$ on the viability of ONS76 cells was evaluated with CCK-8 assays **a** and colony formation assays **b, c** (n = 6, two-way ANOVA and Šídák's multiple comparisons test for CCK-8 assays, one-way ANOVA (one-way ANOVA and Bonferroni's multiple comparisons test for colony formation assays analysis). **d, f** Immunofluorescent co-staining **d** with anti-KIF3A and anti-KI67 and quantification **f** in pGC-FU control, pGC-FU + siKIF3A, siKIF3A + Kif3a^{R} and siKIF3A + Kif3a$^{\Delta E10\text{-}11}$ treated ONS76 cells (one-way ANOVA and Bonferroni's multiple comparisons test). Scale bars = 50 μm and 100 μm. **e, g** EdU incorporation assays **e** and quantification **g** to identify ONS76 cells in the S phase under the same treatment conditions (one-way ANOVA and Bonferroni's multiple comparisons test). Scale bars = 50 μm and 100 μm. Data are presented as mean ± SD (bar plots). $^{*}p < 0.05$, $^{**}p < 0.01$, $^{***}p < 0.001$, $^{****}p < 0.0001$.

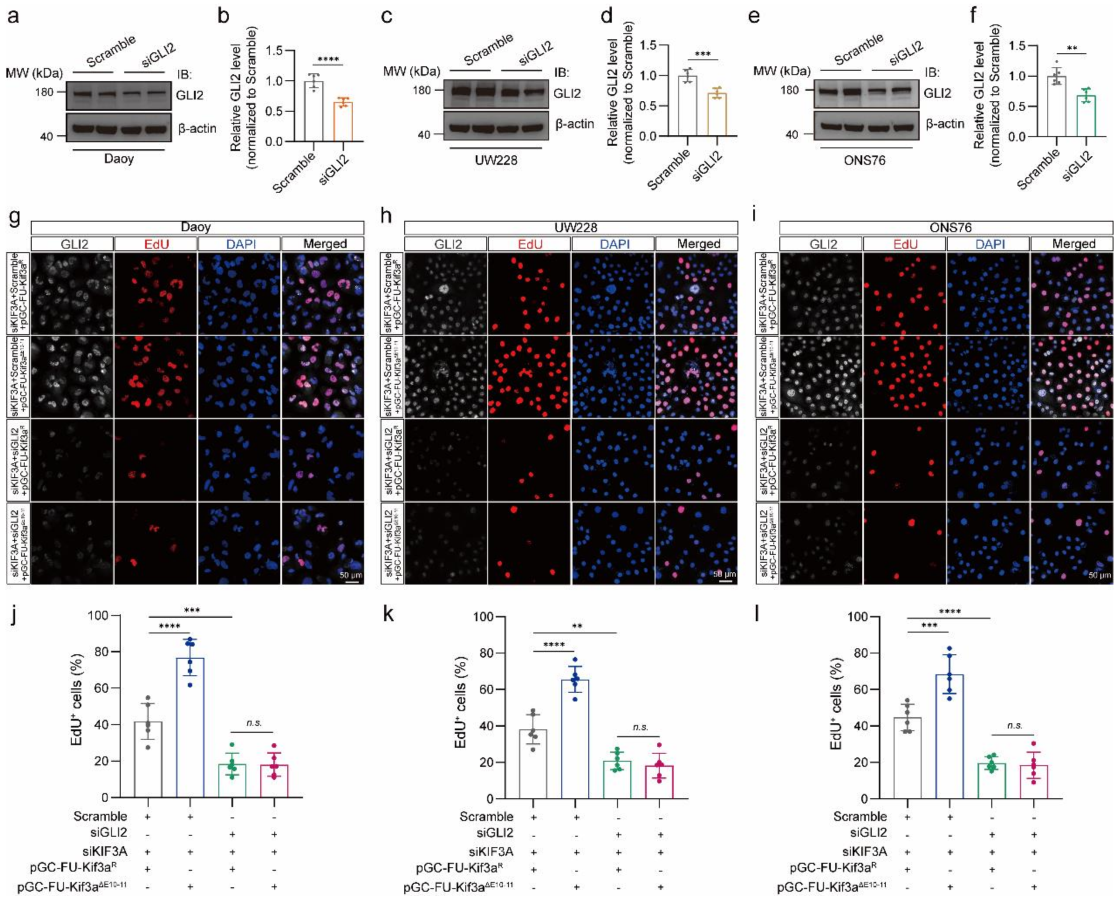

Fig. S16 Skipping of exons 10-11 in *Kif3a* enhances the proliferation of human medulloblastoma cells via the transcriptional factor GLI2. a-f Western blot validation **a, c, e** and quantification **b, d, f** of GLI2 expression following *GLI2* silencing in Daoy **a, b**, UW228 **c, d**, and ONS76 **e, f** cell lines (n = 6, unpaired *t*-test). **g-l** Immunofluorescence staining **g, h, i** and quantification **j, k, l** of the percentage of EdU$^+$ cells in Daoy, UW228 and ONS76 cells treated with siKIF3A, Scramble siRNA or siRNA targeting to GLI2, and lentivirus expressing Kif3a^R or Kif3a$^{\Delta E10-11}$, respectively (n = 6, one-way ANOVA and Bonferroni's multiple comparisons test). Scale bars = 50 µm. Data are presented as mean ± SD (bar plots). $^{**}p < 0.01$, $^{***}p < 0.001$, $^{****}p < 0.0001$, *n.s.*, no significant.

Table S1 Primers used for PCR

**Primers used for genotyping**			
Cre-F	GCCTGCATTACCGGTCGATGCAACGA	*Cre*-R	GTGGCAGATGGCGCGGCAACACCATT
SmoM2-WT-F	CTGGCTTCTGAGGACCG	*SmoM2*-WT-R	AGCCTGCCCAGAAGACTCC
SmoM2-Mutant-F	TCCCCATCAAGATCCATTTC	*SmoM2*-Mutant-R	CTGAACTTGTGGCCGTTTAC
Eftud2$^{fl/fl}$-F	GC AGGAAAGGTTAGCAGTC	*Eftud2*$^{fl/fl}$-R	GTTCTCGTCGGTGGAATA
**Primers used for qRT-PCR**			
Eftud2-F	GTGCTGGGGGAGAACTACAC	*Eftud2*-R	GCCATCAAGCATTTTGGGCA
Hnrnpa1-F	GGTTATGGAAACCAGGGCAGT	*Hnrnpa1*-R	AGTTTCCTCCCTTCATCGGC
Ppil1-F	AGTGGGCATGGTGGAAACAA	*Ppil1*-R	CACACAGCACCCTACGTCAT
Magoh-F	CCGGCAGGAGCTTGAAATTG	*Magoh*-R	CCTTCCGGATCCTTGGACTG
Sf3b1-F	GACTGTCAGGAGTGCTAGGC	*Sf3b1*-R	AACAAGAATCCGTGCGTCCT
Prpf8-F	GCCATGTGCAGTATCGCTTG	*Prpf8*-R	TACAGGACCCGTGTTGAAGC
Tra2b-F	ACGGCGAGCGGGAATC	*Tra2b*-R	ACGCCTTGAGTCTTCCTTGG
Snrpb-F	ATCCCAACAGGTGAGGAAGAG	*Snrpb*-R	TGGGCCCATCATACCTGGA
Ptch1-F	GGGCCATGGACCTGCTTAAT	*Ptch1*-R	GTCTTCTCCTCCTCCTCCGT
Ptch2-F	CAGCTGCTGAGAGGCCTTG	*Ptch2*-R	CTCCAGCCGATGTCATGTGT
Smo-F	AGGGTTCCCAGGGTTGAAGA	*Smo*-R	CCCTCGACTCCCAACTTTGC
Sufu-F	CGATCTGGCCTCATCTGCAT	*Sufu*-R	GTCTGCTCCAGACAGGCTTT
Gli1-F	ATCACCTGTTGGGGATGCTG	*Gli1*-R	AATCGAACTCCTGGCTGCAA
Gli2-F	GCTCTTGTGGTGTGGACTCA	*Gli2*-R	GCACCAAATTTACTGCCTGGA
Gli3-F	AGCAAGTGGTTCCTATGGGC	*Gli3*-R	ATGTTGGAGCAGGGTGGATG

Actb-F	GTGACGTTGACATCCGTAAAGA	*Actb*-R	GCCGGACTCATCGTACTCC
CSNK1G2-F	ATACGTGGCTATCAAATTGGAGC	*CSNK1G2*-R	GGACCGAAGTAGTAGACCTGAG
EVC2-F	ACCACTTGGAATGAAATTGGACA	*EVC2*-R	GCGGTGTGTTATAGGAGACTCT
MGRN1-F	CAACAGCAACGAGTGTGTGG	*MGRN1*-R	ACGCTGTCAGAGTTAGAGTGC
MOSMO-F	CAACCCGGACTGGATCAACA	*MOSMO*-R	GGGCAGCCATACAGAAAAGGA
MEGF8-F	GCACGACCATCACCCTAACA	*MEGF8*-R	ACTGCATACTTGTGGTCCCG
CUL3-F	CGAATCTGAGCAAAGGCACG	*CUL3*-R	ATCTTCTCGCACCGGAAAGG
PTCH1-F	CACACACACAGAGCGGAGTC	*PTCH1*-R	TTTCTTGTGCTCCTCGGCAA
HHIP-F	AGACCGAACAAGTGCCTCTG	*HHIP*-R	TGATCAAGAATACCTGCCCTGG
SMO-F	GTCGGGCCTCCGGAATG	*SMO*-R	GTACCAGCTCTTGGGGTTGT
PRKACA-F	GGCCACTGTTCAACTCCCTT	*PRKACA*-R	ATCTGACTCACTCGGCAACC
SUFU-F	TCCAGATCGTTGGTGTCTGC	*SUFU*-R	AACTCTCTCTTGCAGGTGTGG
GRK3-F	TGCTGGCCGATGTCAGTTAC	*GRK3*-R	CAGGACGATCCTCTTGCTGG
SMURF1-F	CCCGCTCCAAGGCTTCAA	*SMURF1*-R	GAGGTGCACAGAAGCTGGAT
SMURF2-F	TCAAGCTGCGCCTGACA	*SMURF2*-R	TTGAGGCGGTTGATGGCATT
CDON-F	GGCATGATGGGAATCTCCGT	*CDON*-R	GTGGTCACAGGTTGAGCAGA
BTRC-F	CCTCGGCGATTATGGACCC	*BTRC*-R	AGAGACCTGGGCATAGAGCA
GLI2-F	CTGCCTCCGAGAAGCAAGAAG	*GLI2*-R	GCATGGAATGGTGGCAAGAG
CCND1-F	GAGGCGGAGGAGAACAAACA	*CCND1*-R	GGAGGGCGGATTGGAAATGA
FBXW11-F	CCGACTCGGTGATTGAGGAC	*FBXW11*-R	CAGCCTAGCCACAAAGACCT
TPTEP2-CSNK1E-F	TTTCTGACCCAACAGTCCCC	*TPTEP2-CSNK1E*-R	TTTTCTCGGCCTTTTGTGCG
GPR161-F	AGAGAGACATGGAAAAGGCGA	*GPR161*-R	CATGCTGGACGACTCCTCTT

SPOP-F	TGCGGACAGTATGAGGCAAG	*SPOP*-R	CCAAAGTCAGGGGGCAAAGA
KIF3A-F	GCAGTAGCAGCAGCAGTAGT	*KIF3A*-R	GACTGATGCTCTTGTTGGGC
ACTB-F	CTCGCCTTTGCCGATCC	*ACTB*-R	TTCTCCATGTCGTCCCAGTTG
Csnk1g2-F	AGGAGTACATCGACCCTGAGA	*Csnk1g2*-R	CTCTGCTCTTTGCCCAAGTG
Evc2-F	TGCTGGCTGGGAATATCCTG	*Evc2*-R	GGGCTTCTCTTGAAATATCGCT
Mgrn1-F	CCGAAGTCCGCAGGAAACTA	*Mgrn1*-R	TACCTCACAAGCCGGAGAGA
Mosmo-F	CAACCCGGACTGGATCAACA	*Mosmo*-R	AGGGCAGCCATACAGAAAAGG
Megf8-F	TTGTTCAGTCAGACCGGAGC	*Megf8*-R	GCTTCCCAGTGCAACACAAG
Cul3-F	GAAGGACACCAAGATGCGGA	*Cul3*-R	GTCCAGTGTAGAGCTTTTCTCCA
Hhip-F	GAAACGGCTACTACACCCCC	*Hhip*-R	GGGCTGCCCTGGTCAC
Prkaca-F	TTTCTCTTCCTGTTCCCACCC	*Prkaca*-R	TCTTCCTTGGCTTTGGCTAGG
Grk3-F	AGGAAGCCCTGAGCTAATGC	*Grk3*-R	TGTAGGCGTCGTACATCTGC
Smurf1-F	GGCTCTGCAAGGCTCTACA	*Smurf1*-R	TGTGAGCAAGACTCTGTGGC
Smurf2-F	CCTGACAGTACTCTGTGCAAAA	*Smurf2*-R	ATATGCAGGCCTTGAGCGAG
Cdon-F	ATCCAGACCTCGGACCCTTAT	*Cdon*-R	GGCAGTAACAGGTTTAGCAGAAC
Btrc-F	TGCCCAGGACTCGTTAATGT	*Btrc*-R	TGATAGCAGCACTGAGGCAG
Ccnd1-F	AAAATGCCAGAGGCGGATGA	*Ccnd1*-R	GAGGGGGTCCTTGTTTAGCC
Fbxw11-F	GAGCATGCCCAGTGTCAGAT	*Fbxw11*-R	CTGACGGCCTCTTTCTGGAG
Gpr161 -F	GGCTGTATTTCCTGTCGTCCA	*Gpr161* -R	CCCTCTGTTGCAGTGAGGTT
Spop -F	CGTCGACGTGCTGACGA	*Spop* -R	CTCATACTGCCCGCAACATC
Kif3a -F	ATGCCGATCAATAAGTCGGAGA	*Kif3a* -R	GTTCCCCTCATTTCATCCACG
**Primers used for RT-PCR**			

Kif3a exon 9-F	GGGAAGAAGTGTCAGGCTC	*Kif3a exon 9*-R	CCCTTCTCTTTTTCTTTTTTCTCCATC
Kif3a exons 10-11-F	GCAGTAGCAGCAGCAGTAG	*Kif3a exons 10-11*-R	TGCTTGGTGAGGCAAGAA
Kif3a exon 12-F	GAAAGTTTCCCCAGATAAGATGGTG	*Kif3a exon 12*-R	TGGGCTTTCAGAAGGTCCT
Kif3a exons 9-12-F	GGGTGAACTTGGAGAAGATGG	*Kif3a exons 9-12*-R	CTCTCCTCGTCAATTTTCGC
Kif7 exon 10-F	TCGGTAGCGGCAAGGTC	*Kif7 exon 10*-R	CTGTCCGGACCAGCTCA
Kif7 intron 10-F	CAGTCGGTAGCGGCAAG	*Kif7 intron 10*-R	CTGTCCGGACCAGCTCA
Kif7 exon 11-F	AGGCCCTGAACCGTCAA	*Kif7 exon 11*-R	CTGTCCGGACCAGCTCA
Ptch2 exon 15-F	GCCCTGGTGTTGCTGTTC	*Ptch2 exon 15*-R	CTGAGCTGGGCGCTCA
Ptch2 intron 15-F	GTACTTCTCCCTGTACGAGGT	*Ptch2 intron 15*-R	CTGGTGCAGATCAAAGAGGG
Ptch2 exon 16-F	GTATCCAGGCTGCATTTGACC	*Ptch2 exon 16*-R	CTGGCTGAAATCCAGAGGCT
KIF3A exon 9-F	GAGAAGAAATATCAGGCTCTGAT	*KIF3A exon 9*-R	CCCTTCTTTTTTTCCTTTTCTCTC
KIF3A exons 10-11-F	GCAGTAGCAGCAGCAGT	*KIF3A exons 10-11*-R	CTGCTTGATTAGGCAAGAACT
KIF3A exon 12-F	GAAAAAAGAAAGTCTCCCCAGAC	*KIF3A exon 12*-R	TGGGCTTTAAGAAGATCTTTTTCCC

Table S2 Patient information of the medulloblastoma microarray

Microarray of medulloblastoma patients (DC-Bra01022)						
**Position**	**No.**	**Age**	**Sex**	**Organ/Anatomic Site**	**Pathology diagnosis**	**Type**
A1	1	27	F	Cerebellum	Medulloblastoma	Malignant
A2	2	27	F	Cerebellum	Medulloblastoma	Malignant
A3	3	27	F	Cerebellum	Medulloblastoma	Malignant
A4	4	41	M	Cerebellum	Medulloblastoma of vermis cerebellum	Malignant
A5	5	41	M	Cerebellum	Medulloblastoma of vermis cerebellum	Malignant
A6	6	41	M	Cerebellum	Medulloblastoma of vermis cerebellum	Malignant
A7	7	34	M	Cerebellum	Medulloblastoma	Malignant
A8	8	34	M	Cerebellum	Medulloblastoma	Malignant
A9	9	34	M	Cerebellum	Medulloblastoma	Malignant
B1	10	7	M	Cerebellum	Medulloblastoma	Malignant
B2	11	7	M	Cerebellum	Medulloblastoma	Malignant
B3	12	7	M	Cerebellum	Medulloblastoma	Malignant
B4	13	12	F	Cerebellum	Medulloblastoma	Malignant
B5	14	12	F	Cerebellum	Medulloblastoma	Malignant
B6	15	12	F	Cerebellum	Medulloblastoma	Malignant
B7	16	8	F	Cerebellum	Medulloblastoma	Malignant
B8	17	8	F	Cerebellum	Medulloblastoma	Malignant
B9	18	8	F	Cerebellum	Medulloblastoma	Malignant
C1	19	9	M	Cerebellum	Medulloblastoma	Malignant
C2	20	9	M	Cerebellum	Medulloblastoma	Malignant
C3	21	9	M	Cerebellum	Medulloblastoma	Malignant
C4	22	32	F	Cerebellum	Medulloblastoma of posterior cranial fossa	Malignant
C5	23	32	F	Cerebellum	Medulloblastoma of posterior cranial fossa	Malignant
C6	24	32	F	Cerebellum	Medulloblastoma of posterior cranial fossa	Malignant
C7	25	53	M	Cerebellum	Medulloblastoma	Malignant
C8	26	53	M	Cerebellum	Medulloblastoma	Malignant
C9	27	53	M	Cerebellum	Medulloblastoma	Malignant
D1	28	4	F	Cerebellum	Medulloblastoma of vermis cerebellum	Malignant

D2	29	4	F	Cerebellum	Medulloblastoma of vermis cerebellum	Malignant
D3	30	4	F	Cerebellum	Medulloblastoma of vermis cerebellum	Malignant
D4	31	6	F	Cerebellum	Medulloblastoma of vermis cerebellum	Malignant
D5	32	6	F	Cerebellum	Medulloblastoma of vermis cerebellum	Malignant
D6	33	6	F	Cerebellum	Medulloblastoma of vermis cerebellum	Malignant
D7	34	6	M	Cerebellum	Medulloblastoma of vermis cerebellum	Malignant
D8	35	6	M	Cerebellum	Medulloblastoma of vermis cerebellum	Malignant
D9	36	6	M	Cerebellum	Medulloblastoma of vermis cerebellum	Malignant
E1	37	41	M	Cerebellum	Medulloblastoma of posterior cranial fossa	Malignant
E2	38	41	M	Cerebellum	Medulloblastoma of posterior cranial fossa	Malignant
E3	39	41	M	Cerebellum	Medulloblastoma of posterior cranial fossa	Malignant
E4	40	49	M	Cerebellum	Medulloblastoma of posterior cranial fossa	Malignant
E5	41	49	M	Cerebellum	Medulloblastoma of posterior cranial fossa	Malignant
E6	42	49	M	Cerebellum	Medulloblastoma of posterior cranial fossa	Malignant
E7	43	18	F	Cerebellum	Medulloblastoma of the fourth ventricle	Malignant
E8	44	18	F	Cerebellum	Medulloblastoma of the fourth ventricle	Malignant
E9	45	18	F	Cerebellum	Medulloblastoma of the fourth ventricle	Malignant
F1	46	25	M	Cerebellum	Medulloblastoma of the fourth ventricle	Malignant
F2	47	25	M	Cerebellum	Medulloblastoma of the fourth ventricle	Malignant

F3	48	25	M	Cerebellum	Medulloblastoma of the fourth ventricle	Malignant
F4	49	11	M	Cerebellum	Medulloblastoma of the fourth ventricle	Malignant
F5	50	11	M	Cerebellum	Medulloblastoma of the fourth ventricle	Malignant
F6	51	11	M	Cerebellum	Medulloblastoma of the fourth ventricle	Malignant
F7	52	23	M	Cerebellum	Medulloblastoma	Malignant
F8	53	23	M	Cerebellum	Medulloblastoma	Malignant
F9	54	23	M	Cerebellum	Medulloblastoma	Malignant
G1	55	39	F	Cerebellum	Cerebellum tissue	Normal
G2	56	39	F	Cerebellum	Cerebellum tissue	Normal
G3	57	39	F	Cerebellum	Cerebellum tissue	Normal
G4	58	47	M	Cerebellum	Cerebellum tissue	Normal
G5	59	47	M	Cerebellum	Cerebellum tissue	Normal
G6	60	47	M	Cerebellum	Cerebellum tissue	Normal
G7	61	54	F	Cerebellum	Cerebellum tissue	Normal
G8	62	54	F	Cerebellum	Cerebellum tissue	Normal
G9	63	54	F	Cerebellum	Cerebellum tissue	Normal
**Microarray of medulloblastoma patients (N035Cb01)**						
**Position**	**No.**	**Age**	**Sex**	**Organ/Anatomic Site**	**Pathology diagnosis**	**Type**
A1	1	33	M	Cerebellum	Glioblastoma	Malignant
A2	2	65	F	Cerebellum	Glioblastoma	Malignant
A3	3	32	M	Cerebellum	Glioblastoma	Malignant
A4	4	41	M	Cerebellum	Medulloblastoma	Malignant
A5	5	14	F	Cerebellum	Medulloblastoma	Malignant
A6	6	6	M	Cerebellum	Medulloblastoma	Malignant
A7	7	7	M	Cerebellum	Medulloblastoma	Malignant
B1	8	6	F	Cerebellum	Medulloblastoma	Malignant
B2	9	8	M	Cerebellum	Medulloblastoma	Malignant
B3	10	8	F	Cerebellum	Medulloblastoma	Malignant
B4	11	27	F	Cerebellum	Medulloblastoma	Malignant
B5	12	42	M	Cerebellum	Medulloblastoma	Malignant
B6	13	4	M	Cerebellum	Medulloblastoma	Malignant
B7	14	6	M	Cerebellum	Medulloblastoma	Malignant
C1	15	9	M	Cerebellum	Medulloblastoma	Malignant
C2	16	7	M	Cerebellum	Medulloblastoma	Malignant
C3	17	8	M	Cerebellum	Medulloblastoma	Malignant
C4	18	15	F	Cerebellum	Medulloblastoma	Malignant
C5	19	30	F	Cerebellum	Medulloblastoma	Malignant
C6	20	8	F	Cerebellum	Medulloblastoma	Malignant

C7	21	32	M	Cerebellum	Medulloblastoma	Malignant
D1	22	11	F	Cerebellum	Medulloblastoma	Malignant
D2	23	33	M	Cerebellum	Medulloblastoma	Malignant
D3	24	44	M	Cerebellum	Medulloblastoma	Malignant
D4	25	7	M	Cerebellum	Medulloblastoma	Malignant
D5	26	16	M	Cerebellum	Medulloblastoma	Malignant
D6	27	8	M	Cerebellum	Medulloblastoma	Malignant
D7	28	3	M	Cerebellum	Medulloblastoma	Malignant
E1	29	16	M	Cerebellum	Cerebellum tissue	Normal
E2	30	16	M	Cerebellum	Cerebellum tissue	Normal
E3	31	43	M	Cerebellum	Cerebellum tissue	Normal
E4	32	50	M	Cerebellum	Cerebellum tissue	Normal
E5	33	56	M	Cerebellum	Cerebellum tissue	Normal
E6	34	55	M	Cerebellum	Cerebellum tissue	Normal
E7	35	42	F	Cerebellum	Cerebellum tissue	Normal

Table S3 Depth and reads of RNA-seq assay

Sample	Depth (×)	Reads
MB-1 (*SmoM2*^{Atoh1})	10.48	55,533,738
MB-2 (*SmoM2*^{Atoh1})	10.06	53,354,666
MB-3 (*SmoM2*^{Atoh1})	10.13	54,547,890
WT-1 (*SmoM2*$^{fl/+}$)	10.24	49,778,738
WT-2 (*SmoM2*$^{fl/+}$)	10.86	54,478,356
WT-3 (*SmoM2*$^{fl/+}$)	11.33	69,136,908
Daoy-Scramble-1	4.72	44,056,696
Daoy-Scramble-2	5.21	54,048,076
Daoy-Scramble-3	5.12	53,208,302
Daoy-siEFTUD2-1	5.23	50,294,052
Daoy-siEFTUD2-2	5.32	55,813,366
Daoy-siEFTUD2-3	4.94	44,278,120

Table S4 Splicing changes of SHH pathway transcripts in RNA-seq and ISO-seq assays

RNA-seq (Genomic Sequence:NC_000077.7 Chromosome Reference GRCm39 C57BL/6J)										
**Gene**	**AS**	**Chr**	**Strand**	**P Value**	**LongExonStart**	**LongExonEnd**	**ShortES**	**ShortEE**	**FlankingES**	**FlankingEE**
Csnk1e	A3SS	15	-	4.90E-07	79305017	79308255	79305017	79305210	79309039	79309188
Csnk1a1	SE	18	+	0	61704944	61705028	61702534	61702633	61708480	61708620
Btrc	SE	19	+	2.60E-08	45444971	45445049	45411571	45411679	45459453	45459543
Grk2	SE	19	-	0	4338439	4338535	4337850	4338013	4338619	4338686
Grk2	SE	19	-	0.000347	4339925	4340048	4339700	4339767	4340101	4340196
Grk2	MXE	19	-	4.12E-07	4337850	4338013	4338439	4338535	236,144,128	70,90,110
Grk3	SE	5	-	1.02E-05	113085574	113085682	113077538	113077605	113089498	113089593
Csnk1g3	SE	18	+	8.62E-05	54069924	54070020	54066521	54066617	54081119	54081226
Csnk1g3	SE	18	+	0	54081716	54081740	54081119	54081226	54086316	54088620
Csnk1d	SE	11	-	1.52E-09	120855773	120855836	120852574	120854732	120858820	120858960
Csnk1g1	SE	9	+	1.49E-06	65828285	65828350	65816255	65816450	65844078	65844196
Csnk1g1	SE	9	+	0	65942331	65942355	65939520	65939627	65946858	65947011
Ptch2	RI	4	+	4.36E-06	116967391	116967704	282,365,345	42,57,40	8,6,7	0,0,0
Ptch1	SE	13	-	0	63685295	63685422	63682103	63682222	63687046	63687172
Ptch1	MXE	13	-	0	63685295	63685422	63687046	63687172	91712481405	24,20,17
Ptch1	A5SS	13	-	0	63713175	63722709	63720905	63722709	63711436	63711629
Kif3a	SE	11	+	2.01E-09	53481543	53481552	53479515	53479587	53484200	53484357
Kif3a	RI	11	+	1.11E-16	53479515	53481552	554,537,643	4,6,3	482,602,532	28,40,27
Kif3a	MXE	11	+	0	53479515	53479587	53481543	53481552	3,1,3	10,7,10
**Iso-seq (Genomic Sequence: NC_000077.6 Chromosome 11 Reference GRCm38.p6 C57BL/6J)**										
**Gene**	**Splicing**	**chr**	**strand**	**P Value**	**longExonStart**	**longExonEnd**	**shortES**	**shortEE**	**flankingES**	**flankingEE**
Fbxw11	MXE	chr11	+	0.000704	32680200	32680302	32711771	32711997	32652747	32652904
Csnk1a1	SE	chr18	+	0	61571873	61571957	61569463	61569562	61575409	61575549
Kif7	RI	chr7	-	0.000544	79702105	79702282	79702105	79702279	79702452	79702578
Kif7	RI	chr7	-	0.000117	79706735	79706888	79706735	79706885	79706988	79707104
Btrc	SE	chr19	+	2.40E-07	45456532	45456610	45423132	45423240	45471014	45471104
Csnk1g3	SE	chr18	+	7.08E-05	53936852	53936948	53933449	53933545	53948047	53948154
Csnk1g3	SE	chr18	+	7.62E-05	53936852	53936942	53933449	53933545	53948047	53948154
Csnk1g3	SE	chr18	+	0	53948644	53948668	53948047	53948154	53953244	53955578
Csnk1d	SE	chr11	-	2.38E-08	120964947	120965010	120961740	120963906	120967994	120968134
Csnk1g1	SE	chr9	+	1.45E-06	65921003	65921068	65908961	65909168	65936796	65936914
Ptch2	RI	chr4	+	5.21E-06	117110194	117110507	117110194	117110331	117110419	117110507
Adrbk1	SE	chr19	-	0	4288411	4288507	4287822	4287985	4288591	4288658
Kif3a	SE	chr11	+	0	53588688	53588760	53586834	53586930	53590716	53590725
Kif3a	SE	chr11	+	2.20E-07	53590716	53590725	53588688	53588760	53593373	53593530

Table S5 Information of mouse survival assay

Genotype	Sex	Overall survival（Days）
SmoM2$^{fl/+}$	F	> 180
SmoM2$^{fl/+}$	F	> 180
SmoM2$^{fl/+}$	M	> 180
SmoM2$^{fl/+}$	M	> 180
SmoM2$^{fl/+}$	F	> 180
SmoM2$^{fl/+}$	M	> 180
SmoM2$^{fl/+}$	M	> 180
SmoM2$^{fl/+}$	F	> 180
SmoM2$^{fl/+}$	F	> 180
SmoM2$^{fl/+}$	M	> 180
SmoM2$^{fl/+}$	M	> 180
SmoM2$^{fl/+}$	F	> 180
SmoM2$^{fl/+}$	F	> 180
SmoM2$^{fl/+}$	F	> 180
SmoM2$^{fl/+}$	M	> 180
SmoM2$^{fl/+}$	F	> 180
SmoM2^{Atoh1}	F	30
SmoM2^{Atoh1}	M	29
SmoM2^{Atoh1}	M	30
SmoM2^{Atoh1}	F	40
SmoM2^{Atoh1}	M	38
SmoM2^{Atoh1}	M	28
SmoM2^{Atoh1}	F	35
SmoM2^{Atoh1}	F	37
SmoM2^{Atoh1}	F	35
SmoM2^{Atoh1}	M	37
SmoM2^{Atoh1}	M	34
SmoM2^{Atoh1}	F	33
SmoM2^{Atoh1}	M	33
SmoM2^{Atoh1}	F	32
SmoM2^{Atoh1}	F	37
SmoM2^{Atoh1}	M	35
Eftud2^{Atoh1};*SmoM2*^{Atoh1}	M	60
Eftud2^{Atoh1};*SmoM2*^{Atoh1}	F	65
Eftud2^{Atoh1};*SmoM2*^{Atoh1}	M	72
Eftud2^{Atoh1};*SmoM2*^{Atoh1}	M	84
Eftud2^{Atoh1};*SmoM2*^{Atoh1}	F	77
Eftud2^{Atoh1};*SmoM2*^{Atoh1}	F	75
Eftud2^{Atoh1};*SmoM2*^{Atoh1}	M	75
Eftud2^{Atoh1};*SmoM2*^{Atoh1}	F	67

Eftud2^{Atoh1};*SmoM2*^{Atoh1}	M	78
Eftud2^{Atoh1};*SmoM2*^{Atoh1}	M	65
Eftud2^{Atoh1};*SmoM2*^{Atoh1}	F	72
Eftud2^{Atoh1};*SmoM2*^{Atoh1}	M	73
Eftud2^{Atoh1};*SmoM2*^{Atoh1}	F	69
Eftud2^{Atoh1};*SmoM2*^{Atoh1}	F	72
Eftud2^{Atoh1};*SmoM2*^{Atoh1}	F	68
Eftud2^{Atoh1};*SmoM2*^{Atoh1}	F	66

Table S6 Information of statistical data analysis

Position	Replication methods	Statistical methods	Statistical correction	*P* value	*P* value summary	Details
Fig. 1a	Biological replication	Wilcoxon rank-sum test	False discovery rate (FDR)	< 0.0001	****	N/A
Fig. 1b-SE	Biological replication	Unpaired t test	N/A	< 0.0001	****	Two-tailed
Fig. 1b-A5SS	Biological replication	Unpaired t test	N/A	< 0.0001	****	Two-tailed
Fig. 1b-A3SS	Biological replication	Unpaired t test	N/A	< 0.0001	****	Two-tailed
Fig. 1b-MXE	Biological replication	Unpaired t test	N/A	0.0145	*	Two-tailed
Fig. 1b-RI	Biological replication	Unpaired t test	N/A	< 0.0001	****	Two-tailed
Fig. 1c	Biological replication	Wilcoxon rank-sum test	False discovery rate (FDR)	< 0.0001	****	N/A
Fig. 1d-SE	Biological replication	Unpaired t test	N/A	< 0.0001	****	Two-tailed
Fig. 1d-A5SS	Biological replication	Unpaired t test	N/A	< 0.0001	****	Two-tailed
Fig. 1d-A3SS	Biological replication	Unpaired t test	N/A	0.0003	***	Two-tailed
Fig. 1d-MXE	Biological replication	Unpaired t test	N/A	0.1683	*n.s.*	Two-tailed
Fig. 1d-RI	Biological replication	Unpaired t test	N/A	< 0.0001	****	Two-tailed
Fig. 1e	Biological replication	Wilcoxon rank-sum test	False discovery rate (FDR)	< 0.0001	****	N/A
Fig. 1f-SE	Biological replication	Unpaired t test	N/A	0.0008	***	Two-tailed
Fig. 1f-A5SS	Biological replication	Unpaired t test	N/A	< 0.0001	****	Two-tailed
Fig. 1f-A3SS	Biological replication	Unpaired t test	N/A	< 0.0001	****	Two-tailed
Fig. 1f-MXE	Biological replication	Unpaired t test	N/A	0.0366	*	Two-tailed
Fig. 1f-RI	Biological replication	Unpaired t test	N/A	< 0.0001	****	Two-tailed
Fig. 1g	Biological replication	Wilcoxon rank-sum test	False discovery rate (FDR)	< 0.0001	****	N/A
Fig. 1h-SE	Biological replication	Unpaired t test	N/A	0.0008	***	Two-tailed

Fig. 1h-A5SS	Biological replication	Unpaired t test	N/A	0.0009	***	Two-tailed
Fig. 1h-A3SS	Biological replication	Unpaired t test	N/A	0.0003	***	Two-tailed
Fig. 1h-MXE	Biological replication	Unpaired t test	N/A	0.0766	*n.s.*	Two-tailed
Fig. 1h-RI	Biological replication	Unpaired t test	N/A	< 0.0001	****	Two-tailed
Fig. 2e-Eftud2	Biological replication	Unpaired t test	N/A	< 0.0001	****	Two-tailed
Fig. 2e-Hnrnpa1	Biological replication	Unpaired t test	N/A	0.6854	*n.s.*	Two-tailed
Fig. 2e-Snrpb	Biological replication	Unpaired t test	N/A	0.0542	*n.s.*	Two-tailed
Fig. 2e-Ppil1	Biological replication	Unpaired t test	N/A	0.0316	*	Two-tailed
Fig. 2e-Magoh	Biological replication	Unpaired t test	N/A	0.0024	**	Two-tailed
Fig. 2e-Smo	Biological replication	Unpaired t test	N/A	< 0.0001	****	Two-tailed
Fig. 2e-Ptch1	Biological replication	Unpaired t test	N/A	< 0.0001	****	Two-tailed
Fig. 2e-Sufu	Biological replication	Unpaired t test	N/A	< 0.0001	****	Two-tailed
Fig. 2e-Gli1	Biological replication	Unpaired t test	N/A	< 0.0001	****	Two-tailed
Fig. 2e-Gli2	Biological replication	Unpaired t test	N/A	< 0.0001	****	Two-tailed
Fig. 2g	Biological replication	Unpaired t test	N/A	0.0484	*	Two-tailed
Fig. 3b	Biological replication	Log-rank (Mantel-Cox) test (recommended)	Gehan-Breslow-Wilcoxon test	< 0.0001	****	N/A
Fig. 3d-P14 $SmoM2^{fl/+}$ *vs.* $SmoM2^{Atoh1}$	Biological replication	Two-way ANOVA	Šídák's multiple comparisons test	0.9989	*n.s.*	N/A
Fig. 3d-P14 $SmoM2^{fl/+}$ *vs.* $Eftud2^{Atoh1}$;$SmoM2^{Atoh1}$	Biological replication	Two-way ANOVA	Šídák's multiple comparisons test	0.0513	*n.s.*	N/A
Fig. 3d-P14 $SmoM2^{Atoh1}$ *vs.* $Eftud2^{Atoh1}$;$SmoM2^{Atoh1}$	Biological replication	Two-way ANOVA	Šídák's multiple comparisons test	0.0181	*	N/A
Fig. 3d-P28 $SmoM2^{fl/+}$ *vs.* $SmoM2^{Atoh1}$	Biological replication	Two-way ANOVA	Šídák's multiple comparisons test	< 0.0001	****	N/A
Fig. 3d-P28	Biological replication	Two-way ANOVA	Šídák's multiple comparisons test	< 0.0001	****	N/A

$SmoM2^{fl/+}$ *vs.*<br>$Eftud2^{Atoh1};SmoM2^{Atoh1}$						
Fig. 3d-P28<br>$SmoM2^{Atoh1}$ *vs.*<br>$Eftud2^{Atoh1};SmoM2^{Atoh1}$	Biological replication	Two-way ANOVA	Šídák's multiple comparisons test	< 0.0001	****	N/A
Fig. 3e-P14<br>$SmoM2^{fl/+}$ *vs.* $SmoM2^{Atoh1}$	Biological replication	Two-way ANOVA	Šídák's multiple comparisons test	0.0075	**	N/A
Fig. 3e-P14<br>$SmoM2^{fl/+}$ *vs.*<br>$Eftud2^{Atoh1};SmoM2^{Atoh1}$	Biological replication	Two-way ANOVA	Šídák's multiple comparisons test	> 0.9999	*n.s.*	N/A
Fig. 3e-P14<br>$SmoM2^{Atoh1}$ *vs.*<br>$Eftud2^{Atoh1};SmoM2^{Atoh1}$	Biological replication	Two-way ANOVA	Šídák's multiple comparisons test	0.0067	**	N/A
Fig. 3e-P28<br>$SmoM2^{fl/+}$ *vs.* $SmoM2^{Atoh1}$	Biological replication	Two-way ANOVA	Šídák's multiple comparisons test	< 0.0001	****	N/A
Fig. 3e-P28<br>$SmoM2^{fl/+}$ *vs.*<br>$Eftud2^{Atoh1};SmoM2^{Atoh1}$	Biological replication	Two-way ANOVA	Šídák's multiple comparisons test	< 0.0001	****	N/A
Fig. 3e-P28<br>$SmoM2^{Atoh1}$ *vs.*<br>$Eftud2^{Atoh1};SmoM2^{Atoh1}$	Biological replication	Two-way ANOVA	Šídák's multiple comparisons test	< 0.0001	****	N/A
Fig. 3g-P14<br>$SmoM2^{fl/+}$ *vs.* $SmoM2^{Atoh1}$	Biological replication	Two-way ANOVA	Šídák's multiple comparisons test	< 0.0001	****	N/A
Fig. 3g-P14<br>$SmoM2^{fl/+}$ *vs.*<br>$Eftud2^{Atoh1};SmoM2^{Atoh1}$	Biological replication	Two-way ANOVA	Šídák's multiple comparisons test	< 0.0001	****	N/A
Fig. 3g-P14<br>$SmoM2^{Atoh1}$ *vs.*<br>$Eftud2^{Atoh1};SmoM2^{Atoh1}$	Biological replication	Two-way ANOVA	Šídák's multiple comparisons test	< 0.0001	****	N/A
Fig. 3g-P28<br>$SmoM2^{fl/+}$ *vs.* $SmoM2^{Atoh1}$	Biological replication	Two-way ANOVA	Šídák's multiple comparisons test	< 0.0001	****	N/A
Fig. 3g-P28<br>$SmoM2^{fl/+}$ *vs.*<br>$Eftud2^{Atoh1};SmoM2^{Atoh1}$	Biological replication	Two-way ANOVA	Šídák's multiple comparisons test	< 0.0001	****	N/A
Fig. 3g-P28<br>$SmoM2^{Atoh1}$ *vs.*<br>$Eftud2^{Atoh1};SmoM2^{Atoh1}$	Biological replication	Two-way ANOVA	Šídák's multiple comparisons test	< 0.0001	****	N/A
Fig. 3h-P14<br>$SmoM2^{fl/+}$ *vs.* $SmoM2^{Atoh1}$	Biological replication	Two-way ANOVA	Šídák's multiple comparisons test	< 0.0001	****	N/A
Fig. 3h-P14<br>$SmoM2^{fl/+}$ *vs.*<br>$Eftud2^{Atoh1};SmoM2^{Atoh1}$	Biological replication	Two-way ANOVA	Šídák's multiple comparisons test	< 0.0001	****	N/A
Fig. 3h-P14	Biological replication	Two-way ANOVA	Šídák's multiple comparisons test	0.9994	*n.s.*	N/A

$SmoM2^{Atoh1}$ vs. $Eftud2^{Atoh1}$;$SmoM2^{Atoh1}$						
Fig. 3h-P28 *$SmoM2^{fl/+}$ vs. $SmoM2^{Atoh1}$*	Biological replication	Two-way ANOVA	Šídák's multiple comparisons test	< 0.0001	****	N/A
Fig. 3h-P28 *$SmoM2^{fl/+}$ vs. $Eftud2^{Atoh1}$;$SmoM2^{Atoh1}$*	Biological replication	Two-way ANOVA	Šídák's multiple comparisons test	< 0.0001	****	N/A
Fig. 3h-P28 *$SmoM2^{Atoh1}$ vs. $Eftud2^{Atoh1}$;$SmoM2^{Atoh1}$*	Biological replication	Two-way ANOVA	Šídák's multiple comparisons test	0.1314	*n.s.*	N/A
Fig. 4e-EFTUD2	Technical replication	Unpaired t test	N/A	< 0.0001	****	Two-tailed
Fig. 4e-SMO	Technical replication	Unpaired t test	N/A	< 0.0001	****	Two-tailed
Fig. 4e-PTCH1	Technical replication	Unpaired t test	N/A	0.0002	***	Two-tailed
Fig. 4e-SUFU	Technical replication	Unpaired t test	N/A	< 0.0001	****	Two-tailed
Fig. 4e-GLI2	Technical replication	Unpaired t test	N/A	< 0.0001	****	Two-tailed
Fig. 4g-Eftud2	Biological replication	Unpaired t test	N/A	< 0.0001	****	Two-tailed
Fig. 4g-Smo	Biological replication	Unpaired t test	N/A	< 0.0001	****	Two-tailed
Fig. 4g-Ptch1	Biological replication	Unpaired t test	N/A	0.0002	***	Two-tailed
Fig. 4g-Sufu	Biological replication	Unpaired t test	N/A	< 0.0001	****	Two-tailed
Fig. 4g-Gli2	Biological replication	Unpaired t test	N/A	<0.0001	****	Two-tailed
Fig. 4i	Technical replication	Unpaired t test	N/A	0.0010	**	Two-tailed
Fig. 4j	Technical replication	Unpaired t test	N/A	0.0040	**	Two-tailed
Fig. 4k	Technical replication	Unpaired t test	N/A	< 0.0001	****	Two-tailed
Fig. 4l	Technical replication	Unpaired t test	N/A	< 0.0001	****	Two-tailed
Fig. 5a	Biological replication	Wilcoxon rank-sum test	False discovery rate (FDR)	0.026	*	N/A
Fig. 5g *$SmoM2^{fl/+}$ vs. $SmoM2^{Atoh1}$*	Biological replication	Ordinary one-way ANOVA	Bonferroni's multiple comparisons test	0.7001	*n.s.*	N/A
Fig. 5g	Biological replication	Ordinary one-way ANOVA	Bonferroni's multiple comparisons test	0.6270	*n.s.*	N/A

SmoM2^{Atoh1} vs. Eftud2^{Atoh1};SmoM2^{Atoh1}						
Fig. 5l *SmoM2$^{fl/+}$ vs. SmoM2^{Atoh1}*	Biological replication	Ordinary one-way ANOVA	Bonferroni's multiple comparisons test	> 0.9999	*n.s.*	N/A
Fig. 5l *SmoM2^{Atoh1} vs. Eftud2^{Atoh1};SmoM2^{Atoh1}*	Biological replication	Ordinary one-way ANOVA	Bonferroni's multiple comparisons test	> 0.9999	*n.s.*	N/A
Fig. 5m *SmoM2$^{fl/+}$ vs. SmoM2^{Atoh1}*	Biological replication	Ordinary one-way ANOVA	Bonferroni's multiple comparisons test	< 0.0001	****	N/A
Fig. 5m *SmoM2^{Atoh1} vs. Eftud2^{Atoh1};SmoM2^{Atoh1}*	Biological replication	Ordinary one-way ANOVA	Bonferroni's multiple comparisons test	< 0.0001	****	N/A
Fig. 5n *SmoM2$^{fl/+}$ vs. SmoM2^{Atoh1}*	Biological replication	Ordinary one-way ANOVA	Bonferroni's multiple comparisons test	> 0.9999	*n.s.*	N/A
Fig. 5n *SmoM2^{Atoh1} vs. Eftud2^{Atoh1};SmoM2^{Atoh1}*	Biological replication	Ordinary one-way ANOVA	Bonferroni's multiple comparisons test	> 0.9999	*n.s.*	N/A
Fig. 6b pGC-FU *vs.* Scramble	Technical replication	Ordinary one-way ANOVA	Bonferroni's multiple comparisons test	0.5020	*n.s.*	N/A
Fig. 6b Scramble *vs.* pGC-FU+Scramble	Technical replication	Ordinary one-way ANOVA	Bonferroni's multiple comparisons test	> 0.9999	*n.s.*	N/A
Fig. 6b pGC-FU *vs.* pGC-FU+Scramble	Technical replication	Ordinary one-way ANOVA	Bonferroni's multiple comparisons test	0.1405	*n.s.*	N/A
Fig. 6b pGC-FU+Scramble *vs.* pGC-FU+siKIF3A	Technical replication	Ordinary one-way ANOVA	Bonferroni's multiple comparisons test	< 0.0001	****	N/A
Fig. 6b pGC-FU+siKIF3A *vs.* siKIF3A+ pGC-FU-Kif3a^{R}	Technical replication	Ordinary one-way ANOVA	Bonferroni's multiple comparisons test	< 0.0001	****	N/A
Fig. 6b siKIF3A+ pGC-FU-Kif3a^{R} *vs.* siKIF3A+ pGC-FU-Kif3a$^{\Delta E10\text{-}11}$	Technical replication	Ordinary one-way ANOVA	Bonferroni's multiple comparisons test	> 0.9999	*n.s.*	N/A
Fig. 6c pGC-FU *vs.* Scramble	Technical replication	Ordinary one-way ANOVA	Bonferroni's multiple comparisons test	> 0.9999	*n.s.*	N/A
Fig. 6c Scramble *vs.* pGC-FU+Scramble	Technical replication	Ordinary one-way ANOVA	Bonferroni's multiple comparisons test	> 0.9999	*n.s.*	N/A
Fig. 6c pGC-FU *vs.* pGC-FU+Scramble	Technical replication	Ordinary one-way ANOVA	Bonferroni's multiple comparisons test	> 0.9999	*n.s.*	N/A

Fig. 6c pGC-FU+Scramble *vs.* pGC-FU+siKIF3A	Technical replication	Ordinary one-way ANOVA	Bonferroni's multiple comparisons test	< 0.0001	****	N/A
Fig. 6c pGC-FU+siKIF3A *vs.* siKIF3A+ pGC-FU-Kif3a^R	Technical replication	Ordinary one-way ANOVA	Bonferroni's multiple comparisons test	< 0.0001	****	N/A
Fig. 6c siKIF3A+ pGC-FU-Kif3a^R *vs.* siKIF3A+ pGC-FU-Kif3a$^{\Delta E10-11}$	Technical replication	Ordinary one-way ANOVA	Bonferroni's multiple comparisons test	> 0.9999	*n.s.*	N/A
Fig. 6d pGC-FU *vs.* Scramble	Technical replication	Ordinary one-way ANOVA	Bonferroni's multiple comparisons test	> 0.9999	*n.s.*	N/A
Fig. 6d Scramble *vs.* pGC-FU+Scramble	Technical replication	Ordinary one-way ANOVA	Bonferroni's multiple comparisons test	> 0.9999	*n.s.*	N/A
Fig. 6d pGC-FU *vs.* pGC-FU+Scramble	Technical replication	Ordinary one-way ANOVA	Bonferroni's multiple comparisons test	> 0.9999	*n.s.*	N/A
Fig. 6d pGC-FU+Scramble *vs.* pGC-FU+siKIF3A	Technical replication	Ordinary one-way ANOVA	Bonferroni's multiple comparisons test	< 0.0001	****	N/A
Fig. 6d pGC-FU+siKIF3A *vs.* siKIF3A+ pGC-FU-Kif3a^R	Technical replication	Ordinary one-way ANOVA	Bonferroni's multiple comparisons test	< 0.0001	****	N/A
Fig. 6d siKIF3A+ pGC-FU-Kif3a^R *vs.* siKIF3A+ pGC-FU-Kif3a$^{\Delta E10-11}$	Technical replication	Ordinary one-way ANOVA	Bonferroni's multiple comparisons test	< 0.0001	****	N/A
Fig. 6f pcDNA3.1 *vs.* pcDNA3.1-Kif3a^R	Technical replication	Ordinary one-way ANOVA	Bonferroni's multiple comparisons test	< 0.0001	****	N/A
Fig. 6f pcDNA3.1-Kif3a^R *vs.* pcDNA3.1- Kif3a$^{\Delta E10-11}$	Technical replication	Ordinary one-way ANOVA	Bonferroni's multiple comparisons test	< 0.0001	****	N/A
Fig. 6g pcDNA3.1 *vs.* pcDNA3.1-Kif3a^R	Technical replication	Ordinary one-way ANOVA	Bonferroni's multiple comparisons test	0.0086	**	N/A
Fig. 6g pcDNA3.1-Kif3a^R *vs.* pcDNA3.1- Kif3a$^{\Delta E10-11}$	Technical replication	Ordinary one-way ANOVA	Bonferroni's multiple comparisons test	< 0.0001	****	N/A
Fig. 6h pcDNA3.1 *vs.* pcDNA3.1-Kif3a^R	Technical replication	Ordinary one-way ANOVA	Bonferroni's multiple comparisons test	0.0003	***	N/A

Fig. 6h pcDNA3.1-Kif3a^{R} *vs.* pcDNA3.1- Kif3a$^{\Delta E10-11}$	Technical replication	Ordinary one-way ANOVA	Bonferroni's multiple comparisons test	0.0007	***	N/A
Fig. 6i pcDNA3.1 *vs.* pcDNA3.1-Kif3a^{R}	Technical replication	Ordinary one-way ANOVA	Bonferroni's multiple comparisons test	< 0.0001	****	N/A
Fig. 6i pcDNA3.1-Kif3a^{R} *vs.* pcDNA3.1- Kif3a$^{\Delta E10-11}$	Technical replication	Ordinary one-way ANOVA	Bonferroni's multiple comparisons test	< 0.0001	****	N/A
Fig. 7a pGC-FU *vs.* pGC-FU +siKIF3A	Technical replication	Two-way ANOVA	Šídák's multiple comparisons test	< 0.0001	****	N/A
Fig. 7a siKIF3A+ pGC-FU-Kif3a^{R} *vs.* siKIF3A+ pGC-FU-Kif3a$^{\Delta E10-11}$	Technical replication	Two-way ANOVA	Šídák's multiple comparisons test	< 0.0001	****	N/A
Fig. 7c pGC-FU *vs.* pGC-FU+siKIF3A	Technical replication	Ordinary one-way ANOVA	Bonferroni's multiple comparisons test	< 0.0001	****	N/A
Fig. 7c pGC-FU+siKIF3A *vs.* siKIF3A+ pGC-FU-Kif3a^{R}	Technical replication	Ordinary one-way ANOVA	Bonferroni's multiple comparisons test	< 0.0001	****	N/A
Fig. 7c siKIF3A+ pGC-FU-Kif3a^{R} *vs.* siKIF3A+ pGC-FU-Kif3a$^{\Delta E10-11}$	Technical replication	Ordinary one-way ANOVA	Bonferroni's multiple comparisons test	0.0451	*	N/A
Fig. 7f pGC-FU *vs.* pGC-FU+siKIF3A	Technical replication	Ordinary one-way ANOVA	Bonferroni's multiple comparisons test	< 0.0001	****	N/A
Fig. 7f pGC-FU+siKIF3A *vs.* siKIF3A+ pGC-FU-Kif3a^{R}	Technical replication	Ordinary one-way ANOVA	Bonferroni's multiple comparisons test	< 0.0001	****	N/A
Fig. 7f siKIF3A+ pGC-FU-Kif3a^{R} *vs.* siKIF3A+ pGC-FU-Kif3a$^{\Delta E10-11}$	Technical replication	Ordinary one-way ANOVA	Bonferroni's multiple comparisons test	0.0001	***	N/A
Fig. 7g pGC-FU *vs.* pGC-FU+siKIF3A	Technical replication	Ordinary one-way ANOVA	Bonferroni's multiple comparisons test	< 0.0001	****	N/A
Fig. 7g pGC-FU+siKIF3A *vs.* siKIF3A+ pGC-FU-Kif3a^{R}	Technical replication	Ordinary one-way ANOVA	Bonferroni's multiple comparisons test	0.0004	***	N/A
Fig. 7g	Technical replication	Ordinary one-way ANOVA	Bonferroni's multiple comparisons test	< 0.0001	****	N/A

siKIF3A+ pGC-FU-Kif3a^{R} *vs.* siKIF3A+ pGC-FU-Kif3a$^{\Delta E10-11}$						
Fig. S1a-*Eftud2*	Biological replication	Unpaired t test	N/A	< 0.0001	****	Two-tailed
Fig. S1a-*Hnrnpa1*	Biological replication	Unpaired t test	N/A	< 0.0001	****	Two-tailed
Fig. S1a-*Ppil1*	Biological replication	Unpaired t test	N/A	< 0.0001	****	Two-tailed
Fig. S1a-*Magoh*	Biological replication	Unpaired t test	N/A	0.0008	***	Two-tailed
Fig. S1a-*Sf3b1*	Biological replication	Unpaired t test	N/A	0.1776	*n.s.*	Two-tailed
Fig. S1a-*Prpf8*	Biological replication	Unpaired t test	N/A	0.3758	*n.s.*	Two-tailed
Fig. S1a-*Tra2b*	Biological replication	Unpaired t test	N/A	0.1929	*n.s.*	Two-tailed
Fig. S1a-*Snrpb*	Biological replication	Unpaired t test	N/A	0.2076	*n.s.*	Two-tailed
Fig. S1b-*Ptch1*	Biological replication	Unpaired t test	N/A	< 0.0001	****	Two-tailed
Fig. S1b-*Ptch2*	Biological replication	Unpaired t test	N/A	< 0.0001	****	Two-tailed
Fig. S1b-*Smo*	Biological replication	Unpaired t test	N/A	0.0044	**	Two-tailed
Fig. S1b-*Sufu*	Biological replication	Unpaired t test	N/A	0.0018	**	Two-tailed
Fig. S1b-*Gli1*	Biological replication	Unpaired t test	N/A	< 0.0001	****	Two-tailed
Fig. S1b-*Gli2*	Biological replication	Unpaired t test	N/A	< 0.0001	****	Two-tailed
Fig. S1b-*Gli3*	Biological replication	Unpaired t test	N/A	0.0003	***	Two-tailed
Fig. S2a-WNT-subgroup *vs.* Normal	Biological replication	Ordinary one-way ANOVA	Bonferroni's multiple comparisons test	0.0962	*n.s.*	N/A
Fig. S2a-SHH-subgroup *vs.* Normal	Biological replication	Ordinary one-way ANOVA	Bonferroni's multiple comparisons test	0.0183	*	N/A
Fig. S2a-G3-subgroup *vs.* Normal	Biological replication	Ordinary one-way ANOVA	Bonferroni's multiple comparisons test	0.0002	***	N/A
Fig. S2a-G4-subgroup *vs.* Normal	Biological replication	Ordinary one-way ANOVA	Bonferroni's multiple comparisons test	0.0156	*	N/A
Fig. S2b	Biological replication	Log-rank (Mantel-Cox) test (recommended)	Gehan-Breslow-Wilcoxon test	> 0.9999	*n.s.*	N/A

Fig. S3b	Technical replication	Unpaired t test	N/A	< 0.0001	****	Two-tailed
Fig. S3c	Technical replication	Two-way ANOVA	Šídák's multiple comparisons test	< 0.0001	****	N/A
Fig. S3e	Technical replication	Unpaired t test	N/A	< 0.0001	****	Two-tailed
Fig. S3g	Technical replication	Unpaired t test	N/A	< 0.0001	****	Two-tailed
Fig. S3i	Technical replication	Unpaired t test	N/A	< 0.0001	****	Two-tailed
Fig. S3k	Technical replication	Unpaired t test	N/A	0.5390	*n.s.*	Two-tailed
Fig. S3m	Technical replication	Unpaired t test	N/A	0.5681	*n.s.*	Two-tailed
Fig. S4b	Technical replication	Unpaired t test	N/A	< 0.0001	****	Two-tailed
Fig. S4c	Technical replication	Two-way ANOVA	Šídák's multiple comparisons test	< 0.0001	****	N/A
Fig. S4e	Technical replication	Unpaired t test	N/A	< 0.0001	****	Two-tailed
Fig. S4g	Technical replication	Unpaired t test	N/A	< 0.0001	****	Two-tailed
Fig. S4i	Technical replication	Unpaired t test	N/A	0.3512	*n.s.*	Two-tailed
Fig. S4k	Technical replication	Unpaired t test	N/A	< 0.0001	****	Two-tailed
Fig. S4l	Technical replication	Two-way ANOVA	Šídák's multiple comparisons test	< 0.0001	****	N/A
Fig. S4n	Technical replication	Unpaired t test	N/A	< 0.0001	****	Two-tailed
Fig. S4p	Technical replication	Unpaired t test	N/A	< 0.0001	****	Two-tailed
Fig. S4r	Technical replication	Unpaired t test	N/A	0.0847	*n.s.*	Two-tailed
Fig. S5b	Technical replication	Unpaired t test	N/A	< 0.0001	****	Two-tailed
Fig. S5d	Technical replication	Unpaired t test	N/A	0.5915	*n.s.*	Two-tailed
Fig. S5f	Technical replication	Unpaired t test	N/A	< 0.0001	****	Two-tailed
Fig. S5h	Technical replication	Unpaired t test	N/A	0.9895	*n.s.*	Two-tailed
Fig. S5j	Technical replication	Unpaired t test	N/A	< 0.0001	****	Two-tailed

Fig. S5l	Technical replication	Unpaired t test	N/A	0.3268	*n.s.*	Two-tailed
Fig. S6a-*CSNK1G2*	Technical replication	Unpaired t test	N/A	< 0.0001	****	Two-tailed
Fig. S6a-*EVC2*	Technical replication	Unpaired t test	N/A	< 0.0001	****	Two-tailed
Fig. S6a-*MGRN1*	Technical replication	Unpaired t test	N/A	0.3093	*n.s.*	Two-tailed
Fig. S6a-*MOSMO*	Technical replication	Unpaired t test	N/A	< 0.0001	****	Two-tailed
Fig. S6a-*MEGF8*	Technical replication	Unpaired t test	N/A	0.0116	*	Two-tailed
Fig. S6a-*CUL3*	Technical replication	Unpaired t test	N/A	< 0.0001	****	Two-tailed
Fig. S6a-*PTCH1*	Technical replication	Unpaired t test	N/A	< 0.0001	****	Two-tailed
Fig. S6a-*HHIP*	Technical replication	Unpaired t test	N/A	< 0.0001	****	Two-tailed
Fig. S6a-*SMO*	Technical replication	Unpaired t test	N/A	0.0037	**	Two-tailed
Fig. S6a-*PRKACA*	Technical replication	Unpaired t test	N/A	0.0002	***	Two-tailed
Fig. S6a-*SUFU*	Technical replication	Unpaired t test	N/A	< 0.0001	****	Two-tailed
Fig. S6a-*GRK3*	Technical replication	Unpaired t test	N/A	0.0274	*	Two-tailed
Fig. S6a-*SMURF1*	Technical replication	Unpaired t test	N/A	0.0078	**	Two-tailed
Fig. S6a-*SMURF2*	Technical replication	Unpaired t test	N/A	0.6870	*n.s.*	Two-tailed
Fig. S6a-*CDON*	Technical replication	Unpaired t test	N/A	0.4324	*n.s.*	Two-tailed
Fig. S6a-*BTRC*	Technical replication	Unpaired t test	N/A	<0.0001	****	Two-tailed
Fig. S6a-*GLI2*	Technical replication	Unpaired t test	N/A	< 0.0001	****	Two-tailed
Fig. S6a-*CCND1*	Technical replication	Unpaired t test	N/A	< 0.0001	****	Two-tailed
Fig. S6a-*FBXW11*	Technical replication	Unpaired t test	N/A	0.0002	***	Two-tailed
Fig. S6a-*TPTEP2-CSNK1E*	Technical replication	Unpaired t test	N/A	0.0049	**	Two-tailed
Fig. S6a-*GPR161*	Technical replication	Unpaired t test	N/A	0.5581	*n.s.*	Two-tailed

Fig. S6a-*SPOP*	Technical replication	Unpaired t test	N/A	0.5421	*n.s.*	Two-tailed
Fig. S6a-*KIF3A*	Technical replication	Unpaired t test	N/A	0.0016	**	Two-tailed
Fig. S6b-*Csnk1g2*	Biological replication	Unpaired t test	N/A	0.6013	*n.s.*	Two-tailed
Fig. S6b-*Evc2*	Biological replication	Unpaired t test	N/A	0.0126	*	Two-tailed
Fig. S6b-*Mgrn1*	Biological replication	Unpaired t test	N/A	0.0133	*	Two-tailed
Fig. S6b-*Mosmo*	Biological replication	Unpaired t test	N/A	0.0006	***	Two-tailed
Fig. S6b-*Megf8*	Biological replication	Unpaired t test	N/A	< 0.0001	****	Two-tailed
Fig. S6b-*Cul3*	Biological replication	Unpaired t test	N/A	0.0585	*n.s.*	Two-tailed
Fig. S6b-*Ptch1*	Biological replication	Unpaired t test	N/A	< 0.0001	****	Two-tailed
Fig. S6b-*Hhip*	Biological replication	Unpaired t test	N/A	< 0.0001	****	Two-tailed
Fig. S6b-*Smo*	Biological replication	Unpaired t test	N/A	< 0.0001	****	Two-tailed
Fig. S6b-*Prkaca*	Biological replication	Unpaired t test	N/A	< 0.0001	****	Two-tailed
Fig. S6b-*Sufu*	Biological replication	Unpaired t test	N/A	< 0.0001	****	Two-tailed
Fig. S6b-*Grk3*	Biological replication	Unpaired t test	N/A	0.2453	*n.s.*	Two-tailed
Fig. S6b-*Smurf1*	Biological replication	Unpaired t test	N/A	0.6220	*n.s.*	Two-tailed
Fig. S6b-*Smurf2*	Biological replication	Unpaired t test	N/A	< 0.0001	****	Two-tailed
Fig. S6b-*Cdon*	Biological replication	Unpaired t test	N/A	0.0002	***	Two-tailed
Fig. S6b-*Btrc*	Biological replication	Unpaired t test	N/A	0.0002	***	Two-tailed
Fig. S6b-*Gli2*	Biological replication	Unpaired t test	N/A	0.0002	***	Two-tailed
Fig. S6b-*Ccnd1*	Biological replication	Unpaired t test	N/A	0.0484	*	Two-tailed
Fig. S6b-*Fbxw11*	Biological replication	Unpaired t test	N/A	< 0.0001	****	Two-tailed
Fig. S6b-*Gpr161*	Biological replication	Unpaired t test	N/A	0.0002	***	Two-tailed

Fig. S6b-*Spop*	Biological replication	Unpaired t test	N/A	< 0.0001	****	Two-tailed
Fig. S6b-*Kif3a*	Biological replication	Unpaired t test	N/A	0.9396	*n.s.*	Two-tailed
Fig. S7b-EFTUD2	Technical replication	Unpaired t test	N/A	< 0.0001	****	Two-tailed
Fig. S7b-SMO	Technical replication	Unpaired t test	N/A	0.0001	***	Two-tailed
Fig. S7b-PTCH1	Technical replication	Unpaired t test	N/A	0.0607	*n.s.*	Two-tailed
Fig. S7b-SUFU	Technical replication	Unpaired t test	N/A	< 0.0001	****	Two-tailed
Fig. S7b-GLI2	Technical replication	Unpaired t test	N/A	< 0.0001	****	Two-tailed
Fig. S7d-EFTUD2	Technical replication	Unpaired t test	N/A	< 0.0001	****	Two-tailed
Fig. S7d-SMO	Technical replication	Unpaired t test	N/A	0.0002	***	Two-tailed
Fig. S7d-PTCH1	Technical replication	Unpaired t test	N/A	< 0.0001	****	Two-tailed
Fig. S7d-SUFU	Technical replication	Unpaired t test	N/A	< 0.0001	****	Two-tailed
Fig. S7d-GLI2	Technical replication	Unpaired t test	N/A	< 0.0001	****	Two-tailed
Fig. S7f	Technical replication	Unpaired t test	N/A	0.0005	***	Two-tailed
Fig. S7g	Technical replication	Unpaired t test	N/A	< 0.0001	****	Two-tailed
Fig. S7h	Technical replication	Unpaired t test	N/A	< 0.0001	****	Two-tailed
Fig. S7i	Technical replication	Unpaired t test	N/A	0.0001	***	Two-tailed
Fig. S7j	Technical replication	Unpaired t test	N/A	0.0013	**	Two-tailed
Fig. S7k	Technical replication	Unpaired t test	N/A	0.0004	***	Two-tailed
Fig. S7l	Technical replication	Unpaired t test	N/A	< 0.0001	****	Two-tailed
Fig. S7m	Technical replication	Unpaired t test	N/A	< 0.0001	****	Two-tailed
Fig. S8b-Eftud2 $SmoM2^{fl/+}$ *vs.* $SmoM2^{Atoh1}$	Biological replication	Ordinary one-way ANOVA	Bonferroni's multiple comparisons test	0.0002	***	N/A
Fig. S8b-Eftud2	Biological replication	Ordinary one-way ANOVA	Bonferroni's multiple comparisons test	0.9994	*n.s.*	N/A

SmoM2$^{fl/+}$ vs. Eftud2^{Atoh1};SmoM2^{Atoh1}						
Fig. S8b-Eftud2 *SmoM2^{Atoh1} vs. Eftud2^{Atoh1};SmoM2^{Atoh1}*	Biological replication	Ordinary one-way ANOVA	Bonferroni's multiple comparisons test	0.0005	***	N/A
Fig. S8b-Kif3a *SmoM2$^{fl/+}$ vs. SmoM2^{Atoh1}*	Biological replication	Ordinary one-way ANOVA	Bonferroni's multiple comparisons test	0.9482	*n.s.*	N/A
Fig. S8b-Kif3a *SmoM2$^{fl/+}$ vs. Eftud2^{Atoh1};SmoM2^{Atoh1}*	Biological replication	Ordinary one-way ANOVA	Bonferroni's multiple comparisons test	> 0.9999	*n.s.*	N/A
Fig. S8b-Kif3a *SmoM2^{Atoh1} vs. Eftud2^{Atoh1};SmoM2^{Atoh1}*	Biological replication	Ordinary one-way ANOVA	Bonferroni's multiple comparisons test	0.9853	*n.s.*	N/A
Fig. S9b-EFTUD2	Technical replication	Unpaired t test	N/A	< 0.0001	****	Two-tailed
Fig. S9b-KIF3A	Technical replication	Unpaired t test	N/A	0.0003	***	Two-tailed
Fig. S9d-EFTUD2	Technical replication	Unpaired t test	N/A	< 0.0001	****	Two-tailed
Fig. S9d-KIF3A	Technical replication	Unpaired t test	N/A	0.0004	***	Two-tailed
Fig. S9f-EFTUD2	Technical replication	Unpaired t test	N/A	< 0.0001	****	Two-tailed
Fig. S9f-KIF3A	Technical replication	Unpaired t test	N/A	< 0.0001	****	Two-tailed
Fig. S9h-EFTUD2	Technical replication	Unpaired t test	N/A	< 0.0001	****	Two-tailed
Fig. S9h-KIF3A	Technical replication	Unpaired t test	N/A	0.0013	**	Two-tailed
Fig. S9j-EFTUD2	Technical replication	Unpaired t test	N/A	< 0.0001	****	Two-tailed
Fig. S9j-KIF3A	Technical replication	Unpaired t test	N/A	0.0007	***	Two-tailed
Fig. S9l-EFTUD2	Technical replication	Unpaired t test	N/A	< 0.0001	****	Two-tailed
Fig. S9l-KIF3A	Technical replication	Unpaired t test	N/A	0.0005	***	Two-tailed
Fig. S10c *SmoM2$^{fl/+}$ vs. SmoM2^{Atoh1}*	Biological replication	Ordinary one-way ANOVA	Bonferroni's multiple comparisons test	< 0.0001	****	N/A
Fig. S10c *SmoM2^{Atoh1} vs. Eftud2^{Atoh1};SmoM2^{Atoh1}*	Biological replication	Ordinary one-way ANOVA	Bonferroni's multiple comparisons test	< 0.0001	****	N/A
Fig. S10d *SmoM2$^{fl/+}$ vs. SmoM2^{Atoh1}*	Biological replication	Ordinary one-way ANOVA	Bonferroni's multiple comparisons test	< 0.0001	****	N/A

Fig. S10d *SmoM2*^{Atoh1} *vs.* *Eftud2*^{Atoh1};*SmoM2*^{Atoh1}	Biological replication	Ordinary one-way ANOVA	Bonferroni's multiple comparisons test	< 0.0001	****	N/A
Fig. S10i *SmoM2*$^{fl/+}$ *vs.* *SmoM2*^{Atoh1}	Biological replication	Ordinary one-way ANOVA	Bonferroni's multiple comparisons test	> 0.9999	*n.s.*	N/A
Fig. S10i *SmoM2*^{Atoh1} *vs.* *Eftud2*^{Atoh1};*SmoM2*^{Atoh1}	Biological replication	Ordinary one-way ANOVA	Bonferroni's multiple comparisons test	> 0.9999	*n.s.*	N/A
Fig. S10j *SmoM2*$^{fl/+}$ *vs.* *SmoM2*^{Atoh1}	Biological replication	Ordinary one-way ANOVA	Bonferroni's multiple comparisons test	< 0.0001	****	N/A
Fig. S10j *SmoM2*^{Atoh1} *vs.* *Eftud2*^{Atoh1};*SmoM2*^{Atoh1}	Biological replication	Ordinary one-way ANOVA	Bonferroni's multiple comparisons test	< 0.0001	****	N/A
Fig. S10k *SmoM2*$^{fl/+}$ *vs.* *SmoM2*^{Atoh1}	Biological replication	Ordinary one-way ANOVA	Bonferroni's multiple comparisons test	0.6298	*n.s.*	N/A
Fig. S10k *SmoM2*^{Atoh1} *vs.* *Eftud2*^{Atoh1};*SmoM2*^{Atoh1}	Biological replication	Ordinary one-way ANOVA	Bonferroni's multiple comparisons test	0.0702	*n.s.*	N/A
Fig. S10p *SmoM2*$^{fl/+}$ *vs.* *SmoM2*^{Atoh1}	Biological replication	Ordinary one-way ANOVA	Bonferroni's multiple comparisons test	0.7147	*n.s.*	N/A
Fig. S10p *SmoM2*^{Atoh1} *vs.* *Eftud2*^{Atoh1};*SmoM2*^{Atoh1}	Biological replication	Ordinary one-way ANOVA	Bonferroni's multiple comparisons test	> 0.9999	*n.s.*	N/A
Fig. S10q *SmoM2*$^{fl/+}$ *vs.* *SmoM2*^{Atoh1}	Biological replication	Ordinary one-way ANOVA	Bonferroni's multiple comparisons test	< 0.0001	****	N/A
Fig. S10q *SmoM2*^{Atoh1} *vs.* *Eftud2*^{Atoh1};*SmoM2*^{Atoh1}	Biological replication	Ordinary one-way ANOVA	Bonferroni's multiple comparisons test	< 0.0001	****	N/A
Fig. S10r *SmoM2*$^{fl/+}$ *vs.* *SmoM2*^{Atoh1}	Biological replication	Ordinary one-way ANOVA	Bonferroni's multiple comparisons test	0.3896	*n.s.*	N/A
Fig. S10r *SmoM2*^{Atoh1} *vs.* *Eftud2*^{Atoh1};*SmoM2*^{Atoh1}	Biological replication	Ordinary one-way ANOVA	Bonferroni's multiple comparisons test	> 0.9999	*n.s.*	N/A
Fig. S11e	Technical replication	Unpaired t test	N/A	0.5043	*n.s.*	Two-tailed
Fig. S11f	Technical replication	Unpaired t test	N/A	< 0.0001	****	Two-tailed
Fig. S11g	Technical replication	Unpaired t test	N/A	0.4023	*n.s.*	Two-tailed
Fig. S12b	Technical replication	Unpaired t test	N/A	< 0.0001	****	Two-tailed
Fig. S12c	Technical replication	Unpaired t test	N/A	< 0.0001	****	Two-tailed

Fig. S13b pGC-FU *vs.* Scramble	Technical replication	Ordinary one-way ANOVA	Bonferroni's multiple comparisons test	> 0.9999	*n.s.*	N/A
Fig. S13b Scramble *vs.* pGC-FU+Scramble	Technical replication	Ordinary one-way ANOVA	Bonferroni's multiple comparisons test	0.9998	*n.s.*	N/A
Fig. S13b pGC-FU *vs.* pGC-FU+Scramble	Technical replication	Ordinary one-way ANOVA	Bonferroni's multiple comparisons test	0.9992	*n.s.*	N/A
Fig. S13b pGC-FU+Scramble *vs.* pGC-FU+siKIF3A	Technical replication	Ordinary one-way ANOVA	Bonferroni's multiple comparisons test	< 0.0001	****	N/A
Fig. S13b pGC-FU+siKIF3A *vs.* siKIF3A+ pGC-FU-Kif3a^{R}	Technical replication	Ordinary one-way ANOVA	Bonferroni's multiple comparisons test	< 0.0001	****	N/A
Fig. S13b siKIF3A+ pGC-FU-Kif3a^{R} *vs.* siKIF3A+ pGC-FU-Kif3a$^{\Delta E10\text{-}11}$	Technical replication	Ordinary one-way ANOVA	Bonferroni's multiple comparisons test	0.5081	*n.s.*	N/A
Fig. S13c pGC-FU *vs.* Scramble	Technical replication	Ordinary one-way ANOVA	Bonferroni's multiple comparisons test	> 0.9999	*n.s.*	N/A
Fig. S13c Scramble *vs.* pGC-FU+Scramble	Technical replication	Ordinary one-way ANOVA	Bonferroni's multiple comparisons test	> 0.9999	*n.s.*	N/A
Fig. S13c pGC-FU *vs.* pGC-FU+Scramble	Technical replication	Ordinary one-way ANOVA	Bonferroni's multiple comparisons test	0.9991	*n.s.*	N/A
Fig. S13c pGC-FU+Scramble *vs.* pGC-FU+siKIF3A	Technical replication	Ordinary one-way ANOVA	Bonferroni's multiple comparisons test	< 0.0001	****	N/A
Fig. S13c pGC-FU+siKIF3A *vs.* siKIF3A+ pGC-FU-Kif3a^{R}	Technical replication	Ordinary one-way ANOVA	Bonferroni's multiple comparisons test	< 0.0001	****	N/A
Fig. S13c siKIF3A+ pGC-FU-Kif3a^{R} *vs.* siKIF3A+ pGC-FU-Kif3a$^{\Delta E10\text{-}11}$	Technical replication	Ordinary one-way ANOVA	Bonferroni's multiple comparisons test	0.9998	*n.s.*	N/A
Fig. S13d pGC-FU *vs.* Scramble	Technical replication	Ordinary one-way ANOVA	Bonferroni's multiple comparisons test	0.9994	*n.s.*	N/A
Fig. S13d Scramble *vs.* pGC-FU+Scramble	Technical replication	Ordinary one-way ANOVA	Bonferroni's multiple comparisons test	0.9945	*n.s.*	N/A
Fig. S13d pGC-FU *vs.* pGC-FU+Scramble	Technical replication	Ordinary one-way ANOVA	Bonferroni's multiple comparisons test	> 0.9999	*n.s.*	N/A

Fig. S13d pGC-FU+Scramble *vs.* pGC-FU+siKIF3A	Technical replication	Ordinary one-way ANOVA	Bonferroni's multiple comparisons test	< 0.0001	****	N/A
Fig. S13d pGC-FU+siKIF3A *vs.* siKIF3A+ pGC-FU-Kif3a^{R}	Technical replication	Ordinary one-way ANOVA	Bonferroni's multiple comparisons test	< 0.0001	****	N/A
Fig. S13d siKIF3A+ pGC-FU-Kif3a^{R} *vs.* siKIF3A+ pGC-FU-Kif3a$^{\Delta E10\text{-}11}$	Technical replication	Ordinary one-way ANOVA	Bonferroni's multiple comparisons test	< 0.0001	****	N/A
Fig. S13f pGC-FU *vs.* Scramble	Technical replication	Ordinary one-way ANOVA	Bonferroni's multiple comparisons test	0.9855	*n.s.*	N/A
Fig. S13f Scramble *vs.* pGC-FU+Scramble	Technical replication	Ordinary one-way ANOVA	Bonferroni's multiple comparisons test	> 0.9999	*n.s.*	N/A
Fig. S13f pGC-FU *vs.* pGC-FU+Scramble	Technical replication	Ordinary one-way ANOVA	Bonferroni's multiple comparisons test	0.9791	*n.s.*	N/A
Fig. S13f pGC-FU+Scramble *vs.* pGC-FU+siKIF3A	Technical replication	Ordinary one-way ANOVA	Bonferroni's multiple comparisons test	< 0.0001	****	N/A
Fig. S13f pGC-FU+siKIF3A *vs.* siKIF3A+ pGC-FU-Kif3a^{R}	Technical replication	Ordinary one-way ANOVA	Bonferroni's multiple comparisons test	< 0.0001	****	N/A
Fig. S13f siKIF3A+ pGC-FU-Kif3a^{R} *vs.* siKIF3A+ pGC-FU-Kif3a$^{\Delta E10\text{-}11}$	Technical replication	Ordinary one-way ANOVA	Bonferroni's multiple comparisons test	0.8395	*n.s.*	N/A
Fig. S13g pGC-FU *vs.* Scramble	Technical replication	Ordinary one-way ANOVA	Bonferroni's multiple comparisons test	> 0.9999	*n.s.*	N/A
Fig. S13g Scramble *vs.* pGC-FU+Scramble	Technical replication	Ordinary one-way ANOVA	Bonferroni's multiple comparisons test	0.9884	*n.s.*	N/A
Fig. S13g pGC-FU *vs.* pGC-FU+Scramble	Technical replication	Ordinary one-way ANOVA	Bonferroni's multiple comparisons test	0.9921	*n.s.*	N/A
Fig. S13g pGC-FU+Scramble *vs.* pGC-FU+siKIF3A	Technical replication	Ordinary one-way ANOVA	Bonferroni's multiple comparisons test	< 0.0001	****	N/A
Fig. S13g pGC-FU+siKIF3A *vs.* siKIF3A+ pGC-FU-Kif3a^{R}	Technical replication	Ordinary one-way ANOVA	Bonferroni's multiple comparisons test	< 0.0001	****	N/A
Fig. S13g	Technical replication	Ordinary one-way ANOVA	Bonferroni's multiple comparisons test	0.3538	*n.s.*	N/A

siKIF3A+ pGC-FU-Kif3a^{R} *vs.* siKIF3A+ pGC-FU-Kif3a$^{\Delta E10-11}$						
Fig. S13h pGC-FU *vs.* Scramble	Technical replication	Ordinary one-way ANOVA	Bonferroni's multiple comparisons test	> 0.9999	*n.s.*	N/A
Fig. S13h Scramble *vs.* pGC-FU+Scramble	Technical replication	Ordinary one-way ANOVA	Bonferroni's multiple comparisons test	> 0.9999	*n.s.*	N/A
Fig. S13h pGC-FU *vs.* pGC-FU+Scramble	Technical replication	Ordinary one-way ANOVA	Bonferroni's multiple comparisons test	0.9998	*n.s.*	N/A
Fig. S13h pGC-FU+Scramble *vs.* pGC-FU+siKIF3A	Technical replication	Ordinary one-way ANOVA	Bonferroni's multiple comparisons test	< 0.0001	****	N/A
Fig. S13h pGC-FU+siKIF3A *vs.* siKIF3A+ pGC-FU-Kif3a^{R}	Technical replication	Ordinary one-way ANOVA	Bonferroni's multiple comparisons test	< 0.0001	****	N/A
Fig. S13h siKIF3A+ pGC-FU-Kif3a^{R} *vs.* siKIF3A+ pGC-FU-Kif3a$^{\Delta E10-11}$	Technical replication	Ordinary one-way ANOVA	Bonferroni's multiple comparisons test	< 0.0001	****	N/A
Fig. S13j pcDNA3.1 *vs.* pcDNA3.1-Kif3a^{R}	Technical replication	Ordinary one-way ANOVA	Bonferroni's multiple comparisons test	< 0.0001	****	N/A
Fig. S13j pcDNA3.1-Kif3a^{R} *vs.* pcDNA3.1- Kif3a$^{\Delta E10-11}$	Technical replication	Ordinary one-way ANOVA	Bonferroni's multiple comparisons test	0.0060	**	N/A
Fig. S13k pcDNA3.1 *vs.* pcDNA3.1-Kif3a^{R}	Technical replication	Ordinary one-way ANOVA	Bonferroni's multiple comparisons test	< 0.0001	****	N/A
Fig. S13k pcDNA3.1-Kif3a^{R} *vs.* pcDNA3.1- Kif3a$^{\Delta E10-11}$	Technical replication	Ordinary one-way ANOVA	Bonferroni's multiple comparisons test	< 0.0001	****	N/A
Fig. S13l pcDNA3.1 *vs.* pcDNA3.1-Kif3a^{R}	Technical replication	Ordinary one-way ANOVA	Bonferroni's multiple comparisons test	0.0001	***	N/A
Fig. S13l pcDNA3.1-Kif3a^{R} *vs.* pcDNA3.1- Kif3a$^{\Delta E10-11}$	Technical replication	Ordinary one-way ANOVA	Bonferroni's multiple comparisons test	0.0001	***	N/A
Fig. S13m pcDNA3.1 *vs.* pcDNA3.1-Kif3a^{R}	Technical replication	Ordinary one-way ANOVA	Bonferroni's multiple comparisons test	< 0.0001	****	N/A
Fig. S13m	Technical replication	Ordinary one-way ANOVA	Bonferroni's multiple comparisons test	< 0.0001	****	N/A

pcDNA3.1-Kif3a^{R} *vs.* pcDNA3.1- Kif3a$^{\Delta E10-11}$						
Fig. S13n pcDNA3.1 *vs.* pcDNA3.1-Kif3a^{R}	Technical replication	Ordinary one-way ANOVA	Bonferroni's multiple comparisons test	< 0.0001	****	N/A
Fig. S13n pcDNA3.1-Kif3a^{R} *vs.* pcDNA3.1- Kif3a$^{\Delta E10-11}$	Technical replication	Ordinary one-way ANOVA	Bonferroni's multiple comparisons test	0.0008	***	N/A
Fig. S13o pcDNA3.1 *vs.* pcDNA3.1-Kif3a^{R}	Technical replication	Ordinary one-way ANOVA	Bonferroni's multiple comparisons test	< 0.0001	****	N/A
Fig. S13o pcDNA3.1-Kif3a^{R} *vs.* pcDNA3.1- Kif3a$^{\Delta E10-11}$	Technical replication	Ordinary one-way ANOVA	Bonferroni's multiple comparisons test	0.0009	***	N/A
Fig. S13p pcDNA3.1 *vs.* pcDNA3.1-Kif3a^{R}	Technical replication	Ordinary one-way ANOVA	Bonferroni's multiple comparisons test	< 0.0001	****	N/A
Fig. S13p pcDNA3.1-Kif3a^{R} *vs.* pcDNA3.1- Kif3a$^{\Delta E10-11}$	Technical replication	Ordinary one-way ANOVA	Bonferroni's multiple comparisons test	< 0.0001	****	N/A
Fig. S13q pcDNA3.1 *vs.* pcDNA3.1-Kif3a^{R}	Technical replication	Ordinary one-way ANOVA	Bonferroni's multiple comparisons test	0.0053	**	N/A
Fig. S13q pcDNA3.1-Kif3a^{R} *vs.* pcDNA3.1- Kif3a$^{\Delta E10-11}$	Technical replication	Ordinary one-way ANOVA	Bonferroni's multiple comparisons test	0.0016	**	N/A
Fig. S14a pGC-FU *vs.* pGC-FU +siKIF3A	Technical replication	Two-way ANOVA	Šídák's multiple comparisons test	< 0.0001	****	N/A
Fig. S14a siKIF3A+ pGC-FU-Kif3a^{R} *vs.* siKIF3A+ pGC-FU-Kif3a$^{\Delta E10-11}$	Technical replication	Two-way ANOVA	Šídák's multiple comparisons test	< 0.0001	****	N/A
Fig. S14c pGC-FU *vs.* pGC-FU+siKIF3A	Technical replication	Ordinary one-way ANOVA	Bonferroni's multiple comparisons test	< 0.0001	****	N/A
Fig. S14c pGC-FU+siKIF3A *vs.* siKIF3A+ pGC-FU-Kif3a^{R}	Technical replication	Ordinary one-way ANOVA	Bonferroni's multiple comparisons test	< 0.0001	****	N/A
Fig. S14c siKIF3A+ pGC-FU-Kif3a^{R} *vs.* siKIF3A+ pGC-FU-Kif3a$^{\Delta E10-11}$	Technical replication	Ordinary one-way ANOVA	Bonferroni's multiple comparisons test	< 0.0001	****	N/A

Fig. S14f pGC-FU *vs.* pGC-FU+siKIF3A	Technical replication	Ordinary one-way ANOVA	Bonferroni's multiple comparisons test	< 0.0001	****	N/A
Fig. S14f pGC-FU+siKIF3A *vs.* siKIF3A+ pGC-FU-Kif3a^{R}	Technical replication	Ordinary one-way ANOVA	Bonferroni's multiple comparisons test	< 0.0001	****	N/A
Fig. S14f siKIF3A+ pGC-FU-Kif3a^{R} *vs.* siKIF3A+ pGC-FU-Kif3a$^{\Delta E10\text{-}11}$	Technical replication	Ordinary one-way ANOVA	Bonferroni's multiple comparisons test	0.0024	**	N/A
Fig. S14g pGC-FU *vs.* pGC-FU+siKIF3A	Technical replication	Ordinary one-way ANOVA	Bonferroni's multiple comparisons test	0.0285	*	N/A
Fig. S14g pGC-FU+siKIF3A *vs.* siKIF3A+ pGC-FU-Kif3a^{R}	Technical replication	Ordinary one-way ANOVA	Bonferroni's multiple comparisons test	0.0008	***	N/A
Fig. S14g siKIF3A+ pGC-FU-Kif3a^{R} *vs.* siKIF3A+ pGC-FU-Kif3a$^{\Delta E10\text{-}11}$	Technical replication	Ordinary one-way ANOVA	Bonferroni's multiple comparisons test	0.0003	***	N/A
Fig. S15a pGC-FU *vs.* pGC-FU +siKIF3A	Technical replication	Two-way ANOVA	Šídák's multiple comparisons test	< 0.0001	****	N/A
Fig. S15a siKIF3A+ pGC-FU-Kif3a^{R} *vs.* siKIF3A+ pGC-FU-Kif3a$^{\Delta E10\text{-}11}$	Technical replication	Two-way ANOVA	Šídák's multiple comparisons test	< 0.0001	****	N/A
Fig. S15c pGC-FU *vs.* pGC-FU+siKIF3A	Technical replication	Ordinary one-way ANOVA	Bonferroni's multiple comparisons test	< 0.0001	****	N/A
Fig. S15c pGC-FU+siKIF3A *vs.* siKIF3A+ pGC-FU-Kif3a^{R}	Technical replication	Ordinary one-way ANOVA	Bonferroni's multiple comparisons test	< 0.0001	****	N/A
Fig. S15c siKIF3A+ pGC-FU-Kif3a^{R} *vs.* siKIF3A+ pGC-FU-Kif3a$^{\Delta E10\text{-}11}$	Technical replication	Ordinary one-way ANOVA	Bonferroni's multiple comparisons test	< 0.0001	****	N/A
Fig. S15f pGC-FU *vs.* pGC-FU+siKIF3A	Technical replication	Ordinary one-way ANOVA	Bonferroni's multiple comparisons test	< 0.0001	****	N/A
Fig. S15f pGC-FU+siKIF3A *vs.* siKIF3A+ pGC-FU-Kif3a^{R}	Technical replication	Ordinary one-way ANOVA	Bonferroni's multiple comparisons test	< 0.0001	****	N/A

Fig. S15f siKIF3A+ pGC-FU-Kif3a^{R} *vs.* siKIF3A+ pGC-FU-Kif3a$^{\Delta E10-11}$	Technical replication	Ordinary one-way ANOVA	Bonferroni's multiple comparisons test	0.0010	***	N/A
Fig. S15g pGC-FU *vs.* pGC-FU+siKIF3A	Technical replication	Ordinary one-way ANOVA	Bonferroni's multiple comparisons test	0.0332	*	N/A
Fig. S15g pGC-FU+siKIF3A *vs.* siKIF3A+ pGC-FU-Kif3a^{R}	Technical replication	Ordinary one-way ANOVA	Bonferroni's multiple comparisons test	0.0021	**	N/A
Fig. S15g siKIF3A+ pGC-FU-Kif3a^{R} *vs.* siKIF3A+ pGC-FU-Kif3a$^{\Delta E10-11}$	Technical replication	Ordinary one-way ANOVA	Bonferroni's multiple comparisons test	< 0.0001	****	N/A
Fig. S16b	Technical replication	Unpaired t test	N/A	< 0.0001	****	Two-tailed
Fig. S16d	Technical replication	Unpaired t test	N/A	0.0002	***	Two-tailed
Fig. S16f	Technical replication	Unpaired t test	N/A	0.0011	**	Two-tailed
Fig. S16j-Scramble+siKIF3A+Kif3a^{R} *vs.* Scramble+siKIF3A +Kif3a$^{\Delta E10-11}$	Technical replication	Ordinary one-way ANOVA	Bonferroni's multiple comparisons test	< 0.0001	****	N/A
Fig. S16j-Scramble+siKIF3A+Kif3a^{R} *vs.* siGli2+siKIF3A+Kif3a^{R}	Technical replication	Ordinary one-way ANOVA	Bonferroni's multiple comparisons test	0.0005	***	N/A
Fig. S16j-siGli2+siKIF3A+Kif3a^{R} *vs.* siGli2+siKIF3A+Kif3a$^{\Delta E10-11}$	Technical replication	Ordinary one-way ANOVA	Bonferroni's multiple comparisons test	> 0.9999	*n.s.*	N/A
Fig. S16k-Scramble+siKIF3A+Kif3a^{R} *vs.* Scramble+siKIF3A +Kif3a$^{\Delta E10-11}$	Technical replication	Ordinary one-way ANOVA	Bonferroni's multiple comparisons test	< 0.0001	****	N/A
Fig. S16k-Scramble+siKIF3A+Kif3a^{R} *vs.* siGli2+siKIF3A+Kif3a^{R}	Technical replication	Ordinary one-way ANOVA	Bonferroni's multiple comparisons test	0.0017	**	N/A
Fig. S16k-siGli2+siKIF3A+Kif3a^{R} *vs.* siGli2+siKIF3A+Kif3a$^{\Delta E10-11}$	Technical replication	Ordinary one-way ANOVA	Bonferroni's multiple comparisons test	> 0.9999	*n.s.*	N/A
Fig. S16k-Scramble+siKIF3A+Kif3a^{R} *vs.* Scramble+siKIF3A +Kif3a$^{\Delta E10-11}$	Technical replication	Ordinary one-way ANOVA	Bonferroni's multiple comparisons test	0.0002	***	N/A

Fig. S16k-Scramble+siKIF3A+Kif3a^{R} *vs.* siGli2+siKIF3A+Kif3a^{R}	Technical replication	Ordinary one-way ANOVA	Bonferroni's multiple comparisons test	< 0.0001	****	N/A
Fig. S16k-siGli2+siKIF3A+Kif3a^{R} *vs.* siGli2+siKIF3A+Kif3a$^{\Delta E10\text{-}11}$	Technical replication	Ordinary one-way ANOVA	Bonferroni's multiple comparisons test	> 0.9999	*n.s.*	N/A
