# Supplementary material for: Alternative spliceosomal protein Eftud2 mediated Kif3a exon skipping promotes SHH-subgroup medulloblastoma progression
Source: Cell Death Differ. 2025 Apr 24;32(10):1930–45. doi: 10.1038/s41418-025-01512-9 (PMC12501224; doi:10.1038/s41418-025-01512-9)

Figure 2d

Eftud2


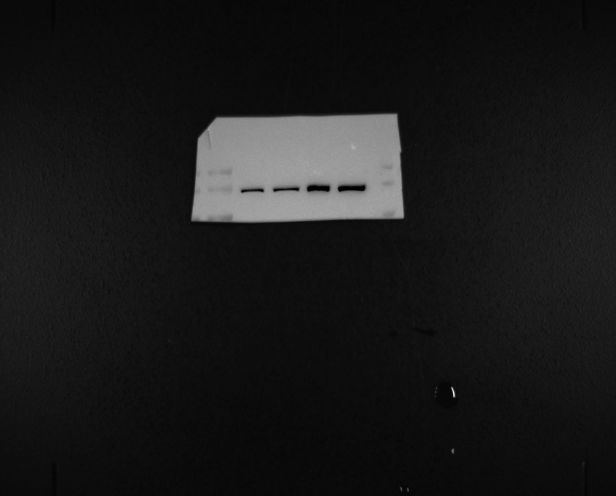


Hnrnpa1


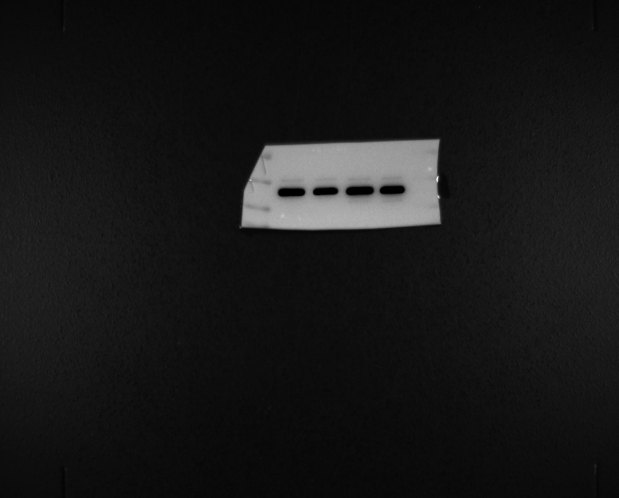


Snrpb


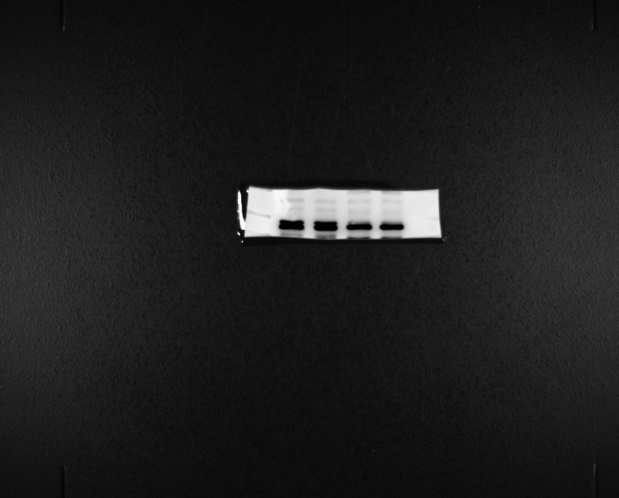


Ppil1

**
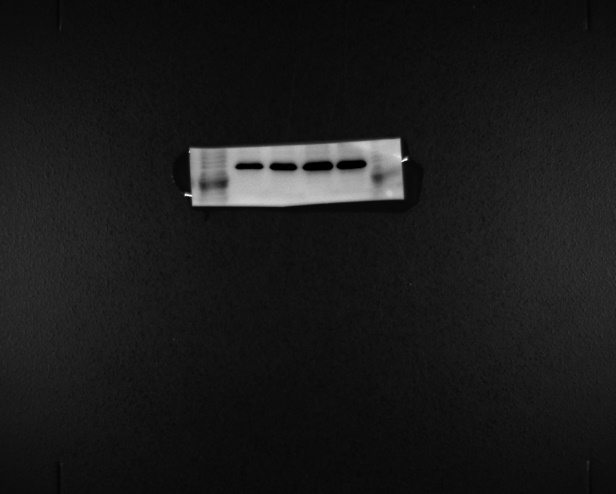
**

Magoh


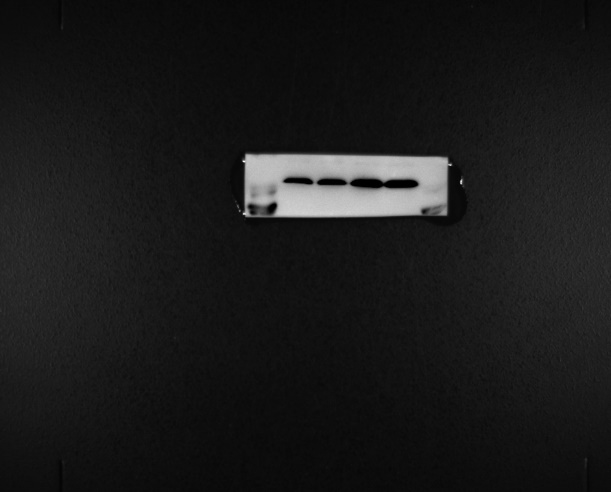


Smo


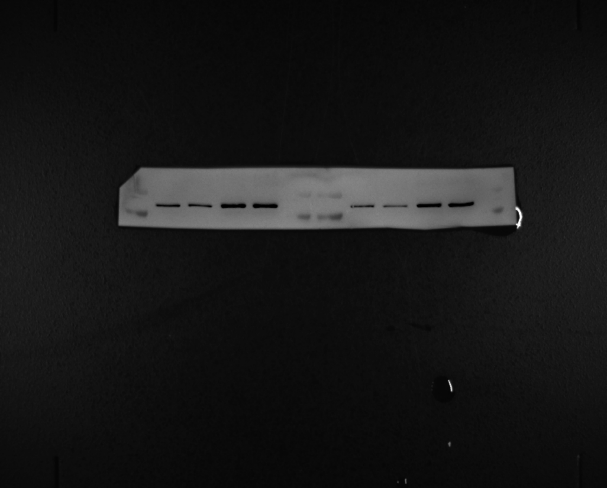


Ptch1


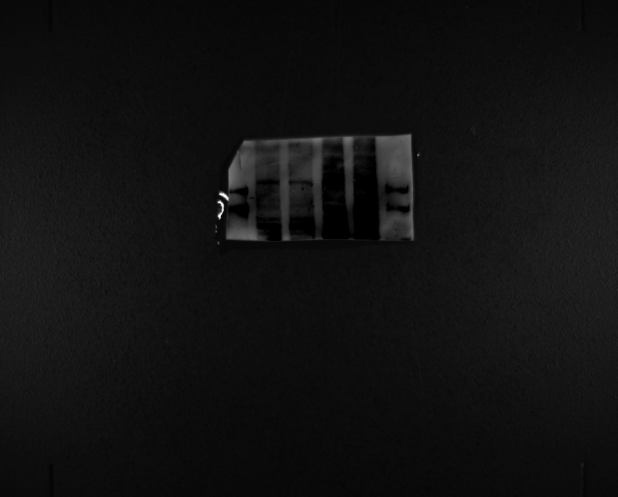


Sufu


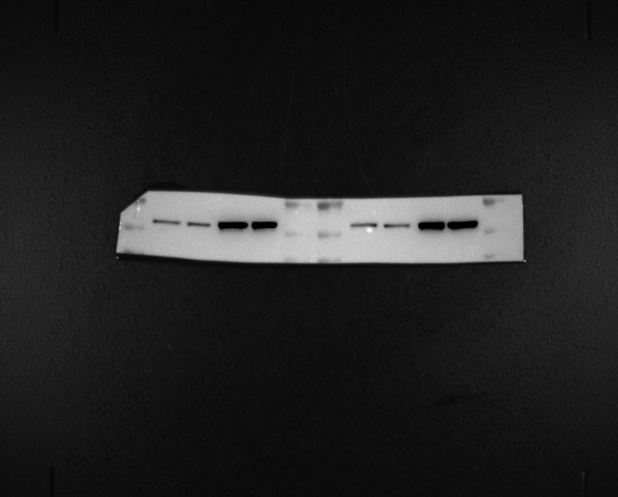


Gli1


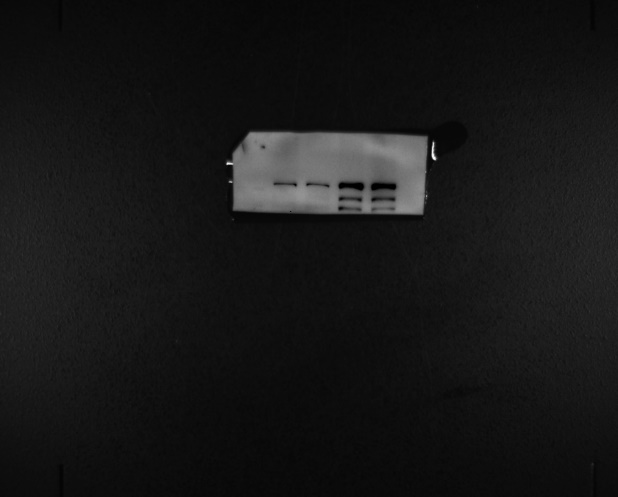


Gli2


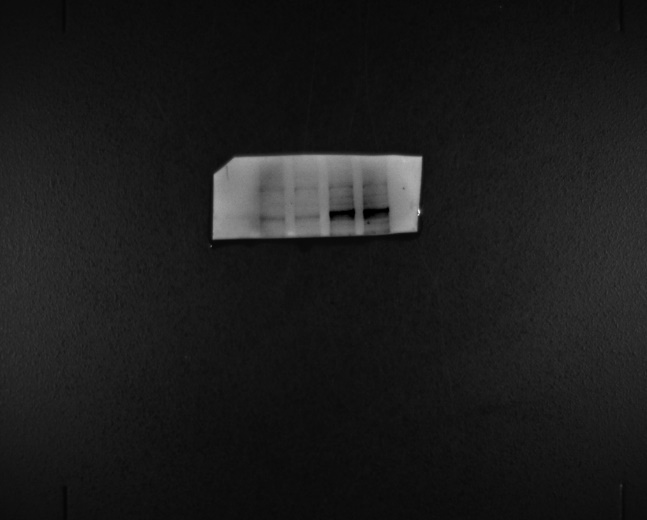


β-actin


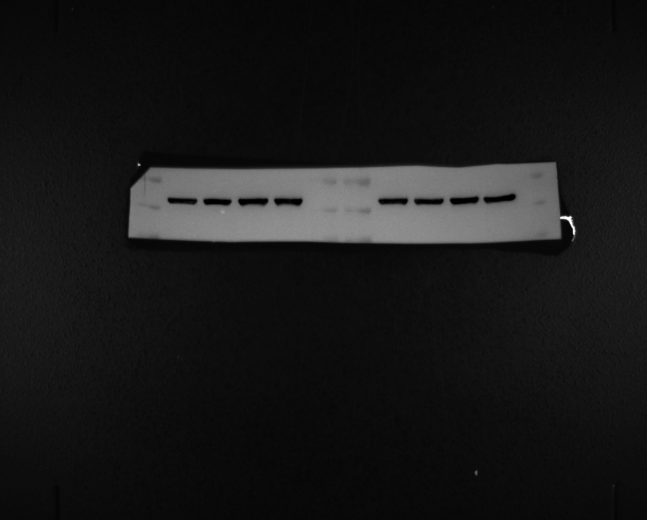


Figure 4d

EFTUD2


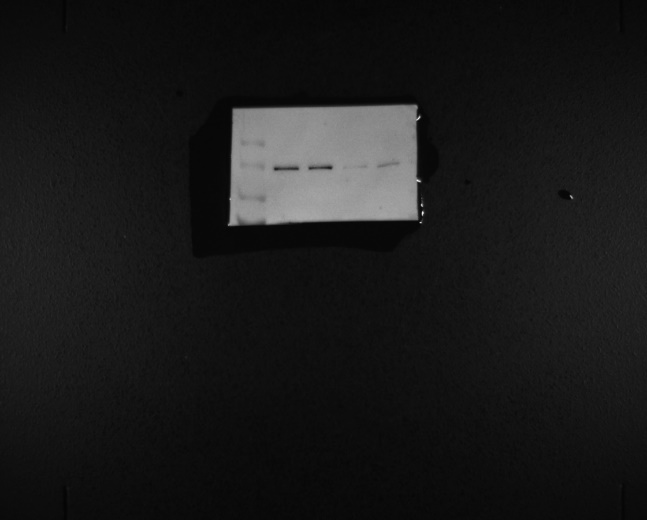


SMO


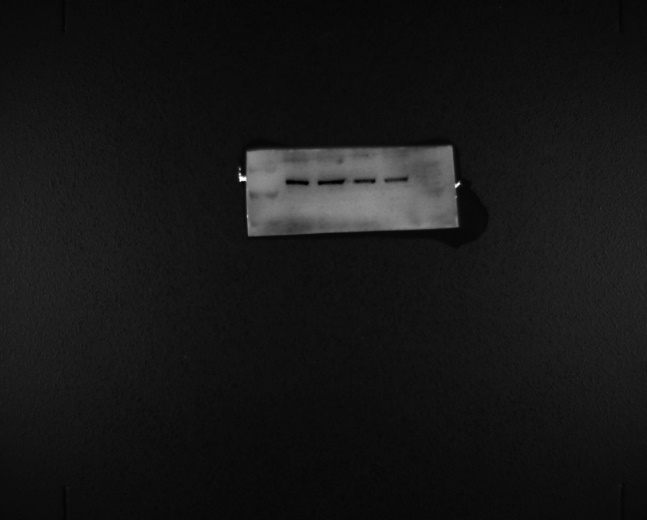


PTCH1


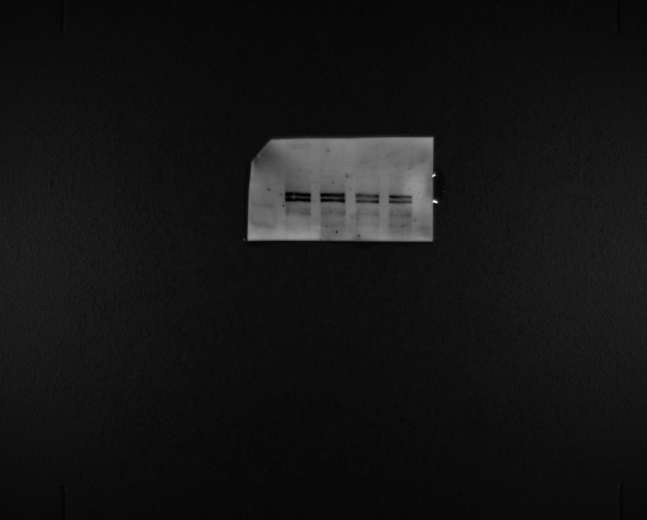


SUFU


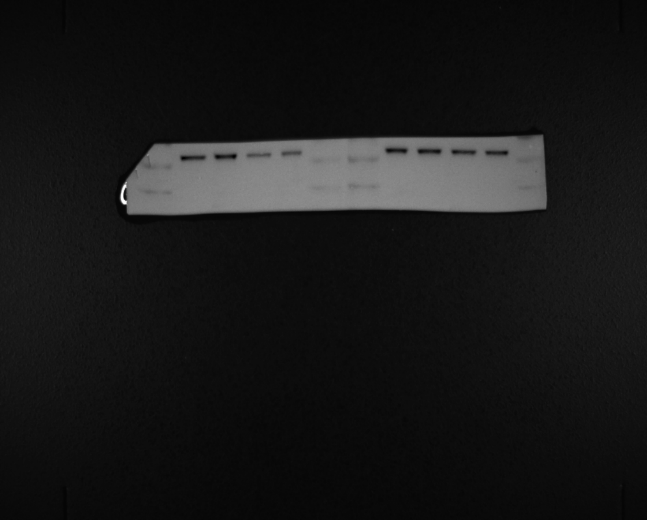


GLI2


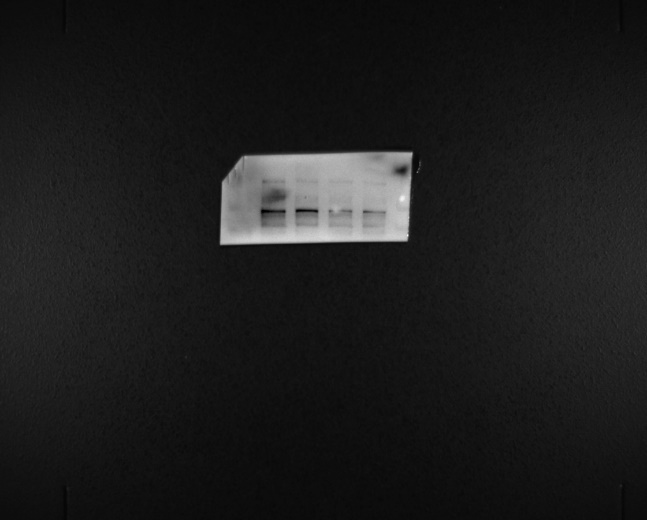


β-actin


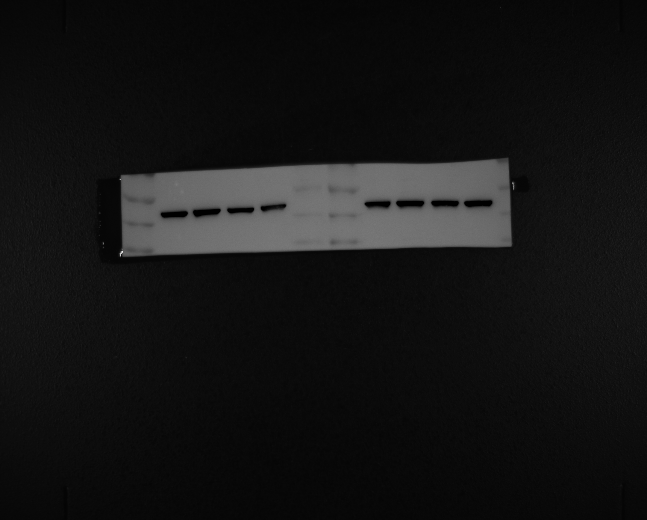


Figure 4f

Eftud2


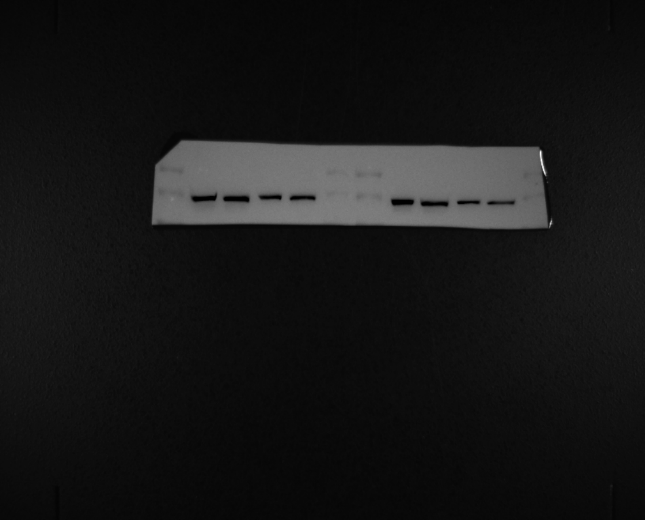


Smo


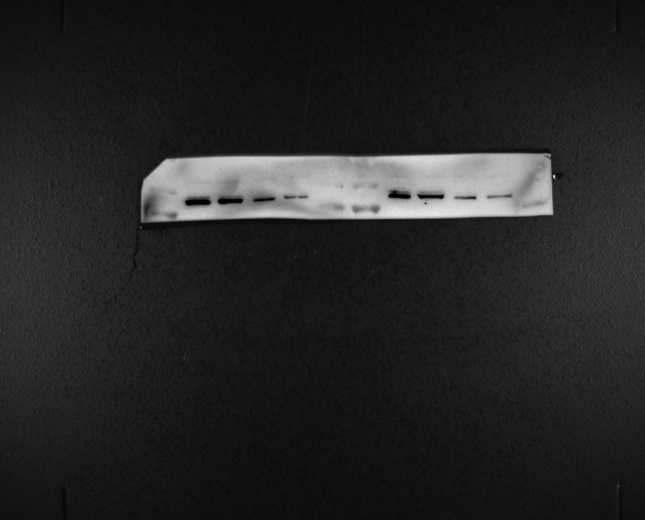


Ptch1


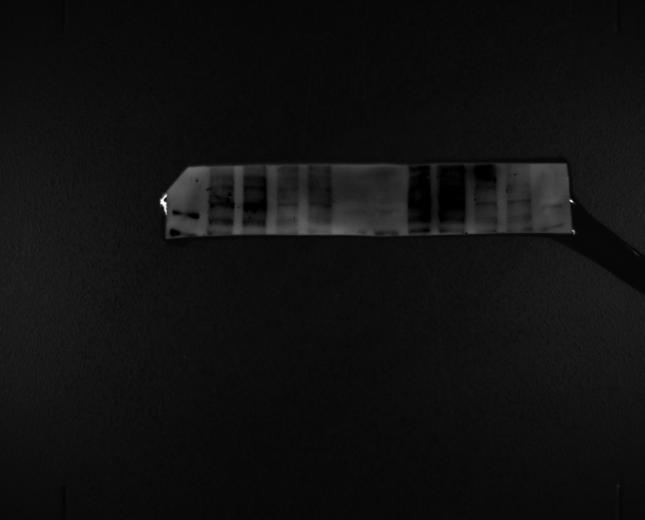


Sufu


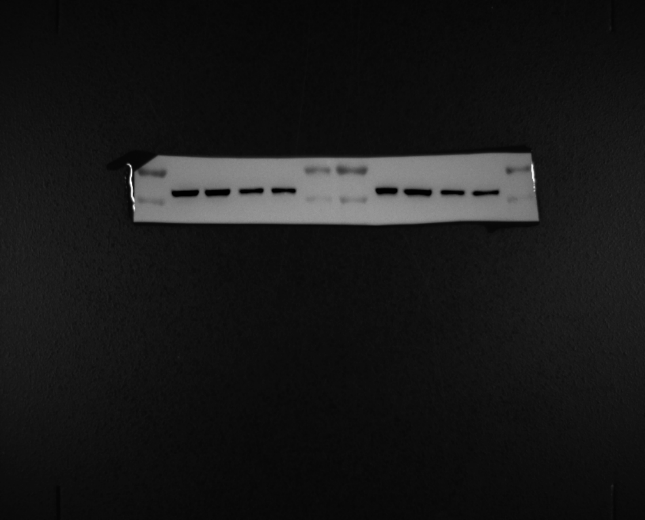


Gli2


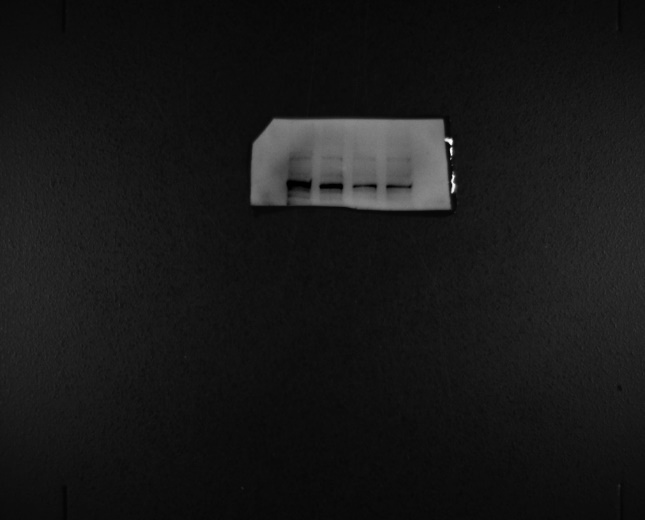


β-actin


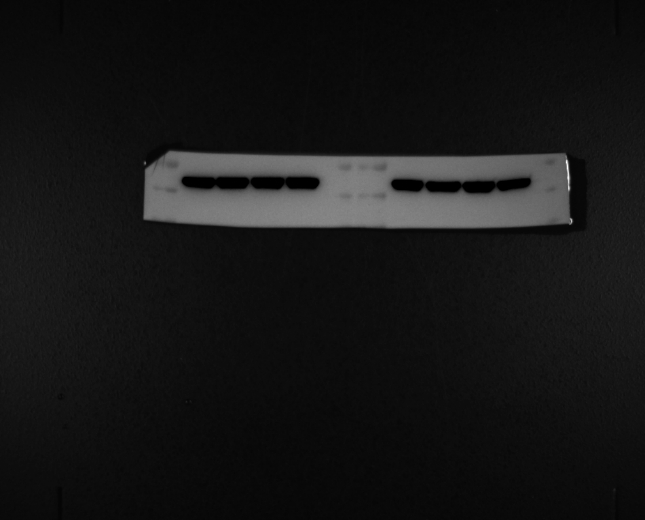


Figure 5e

Eftud2


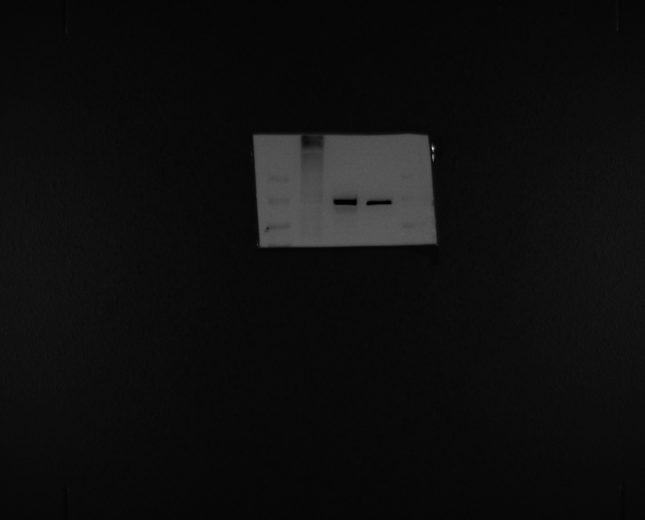


Figure 5f


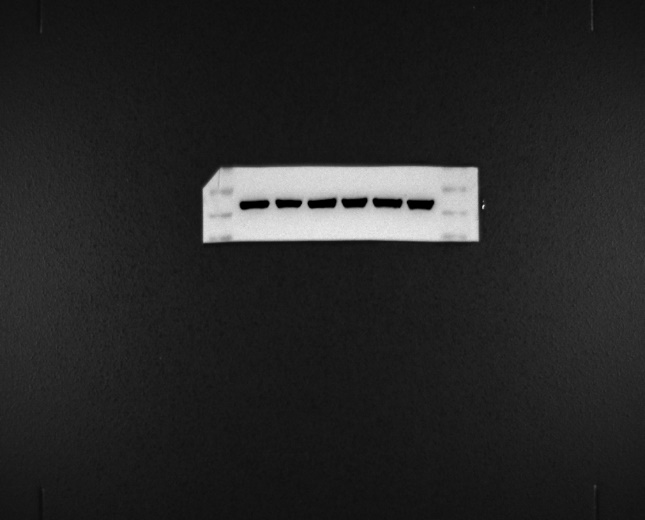

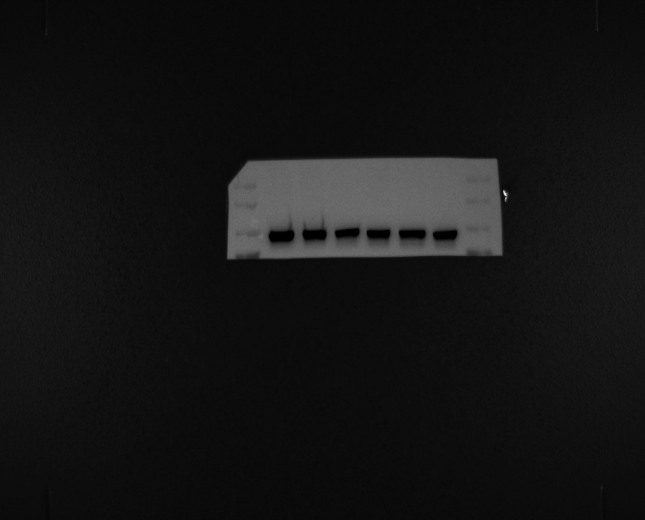
Kif3a β-actin

Figure 6a


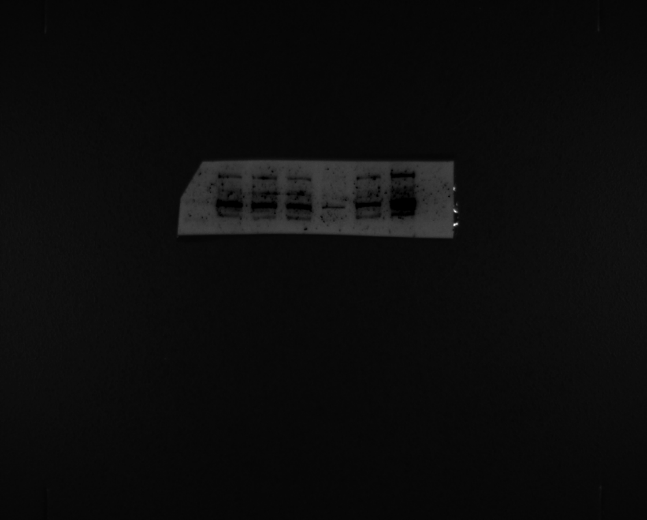
GLI1 GLI2


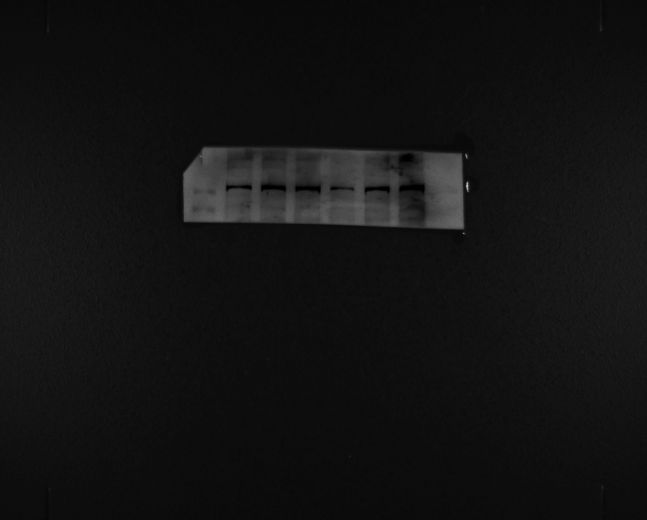


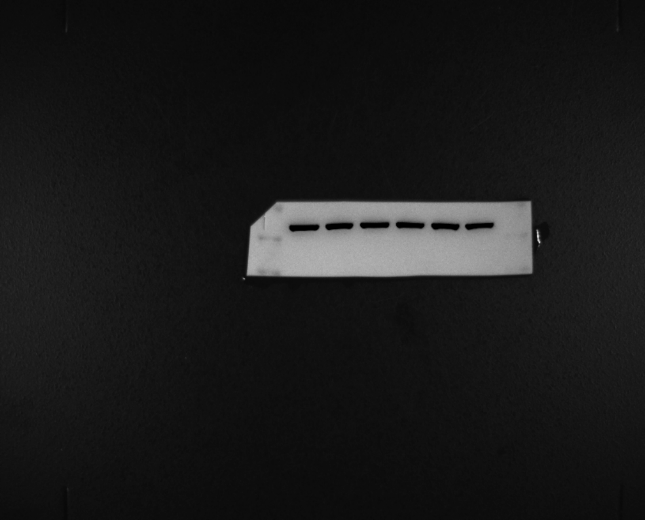
KIF3A β-actin


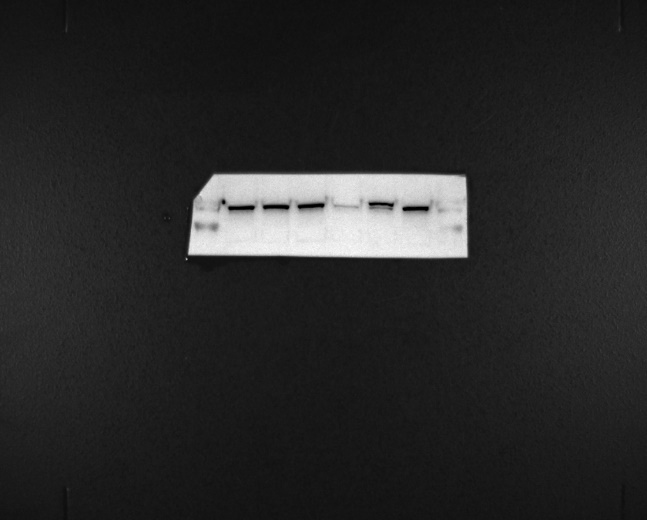


Supplementary Figure 3a

EFTUD2 β-actin


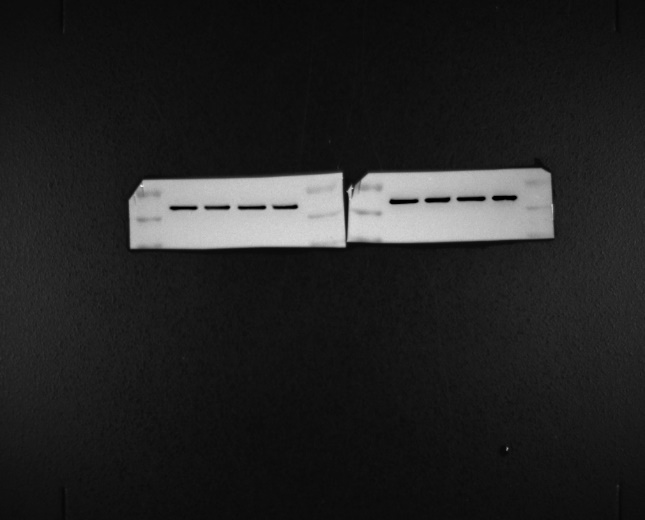

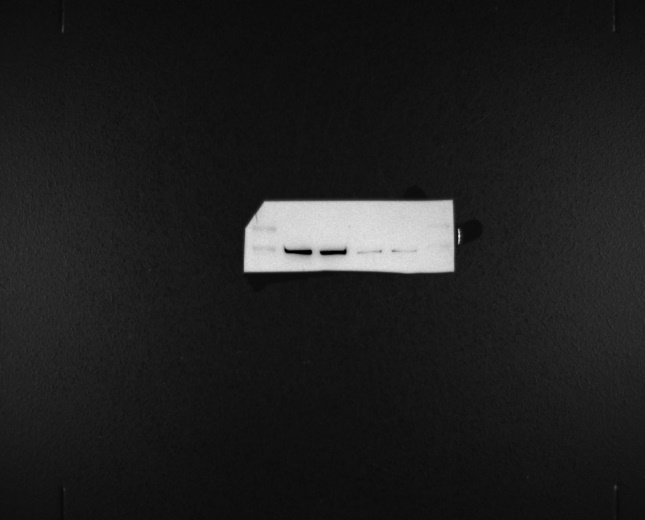


Supplementary Figure 4a

EFTUD2 β-actin


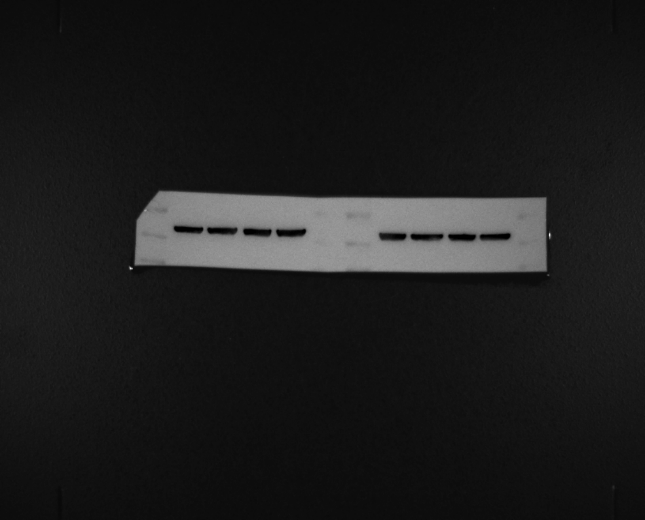

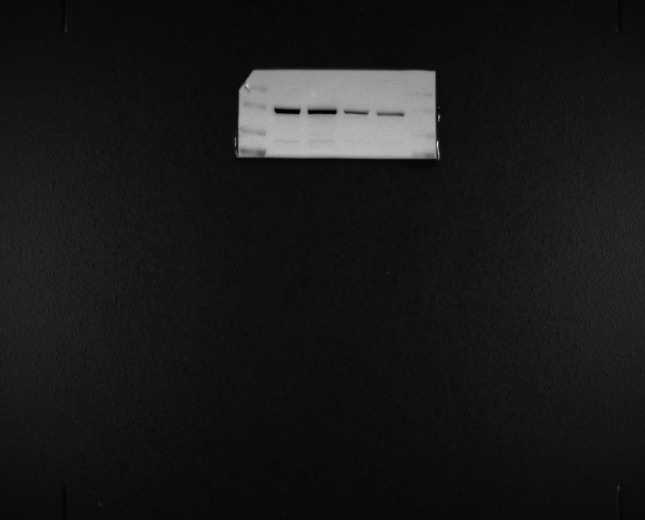


Supplementary Figure 4j

EFTUD2 β-actin


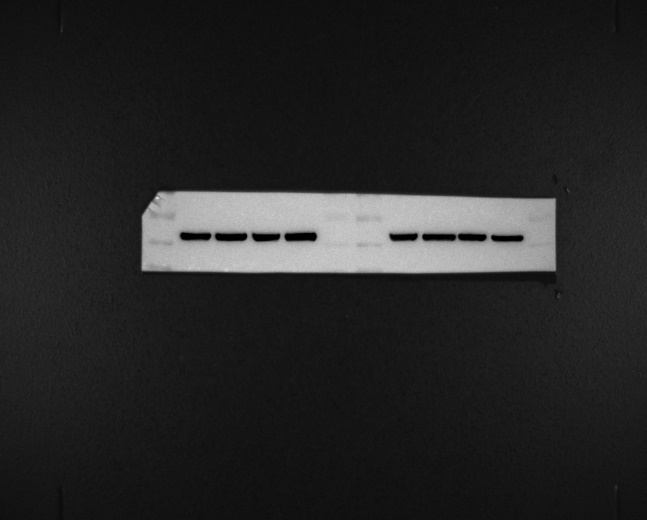

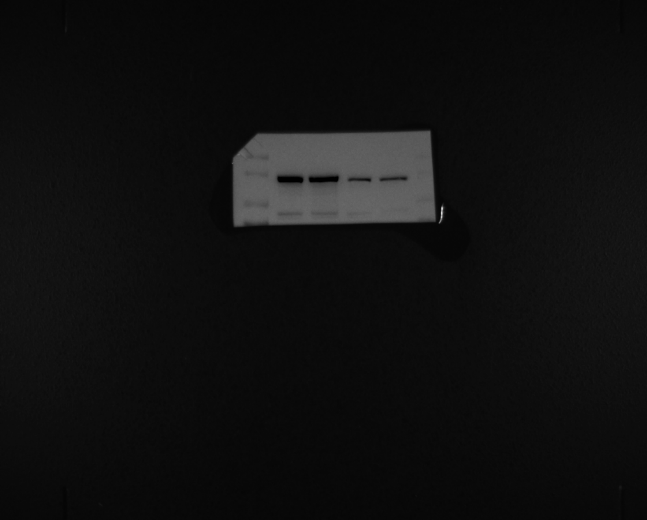


Supplementary Figure 7a

EFTUD2


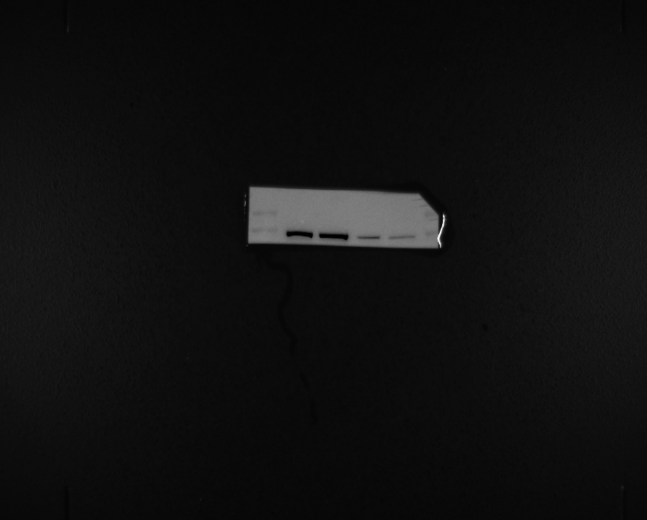


SMO


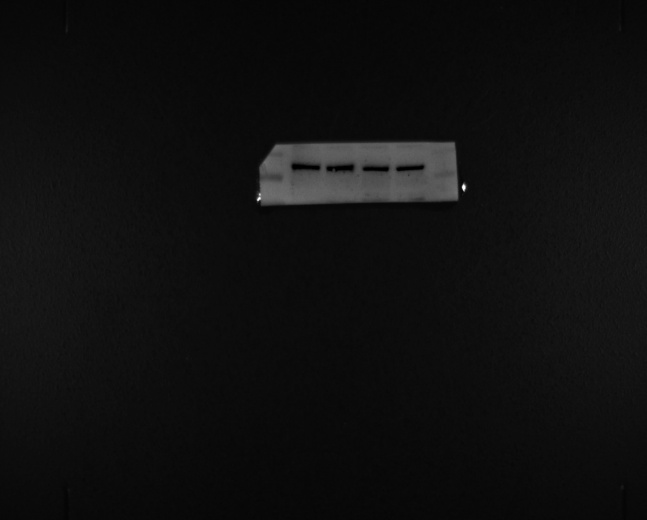


PTCH1


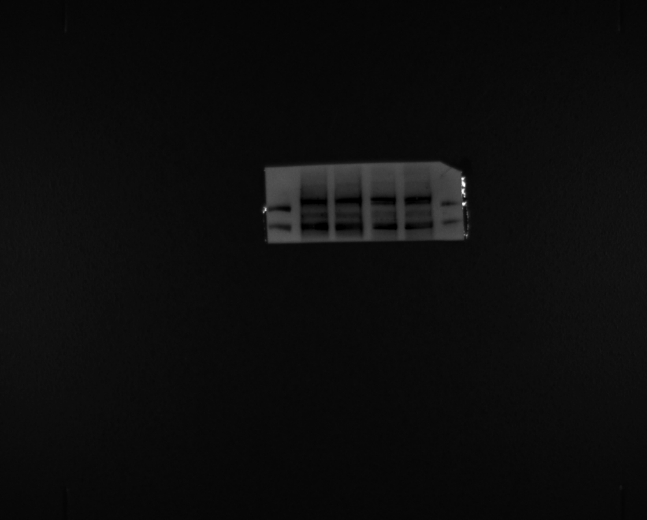


SUFU


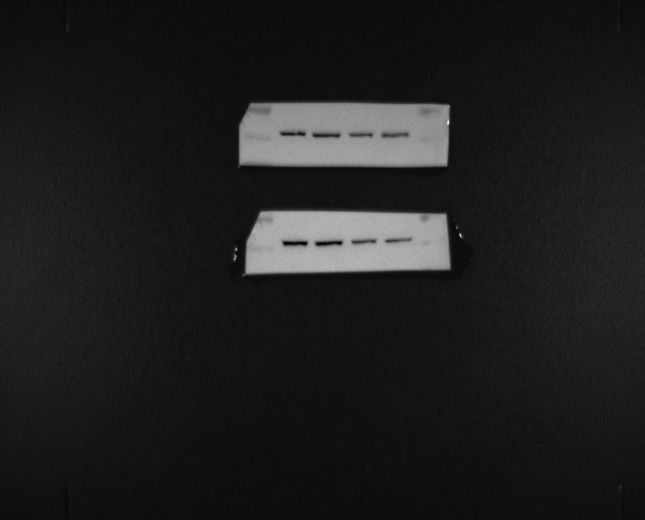


GLI2


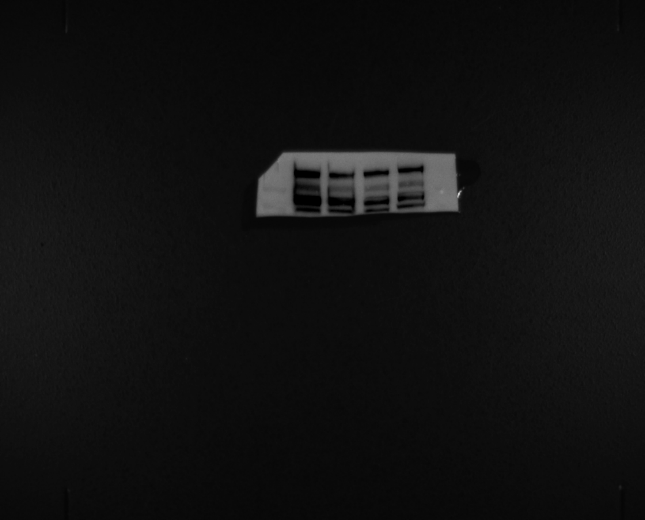


β-actin


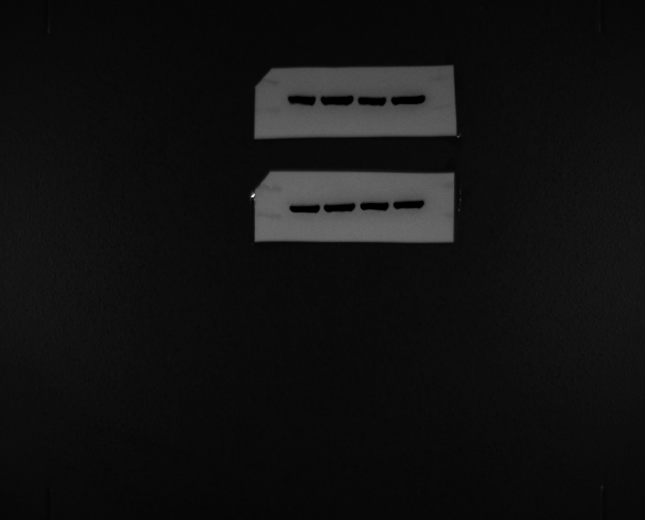


Supplementary Figure 7c

EFTUD2


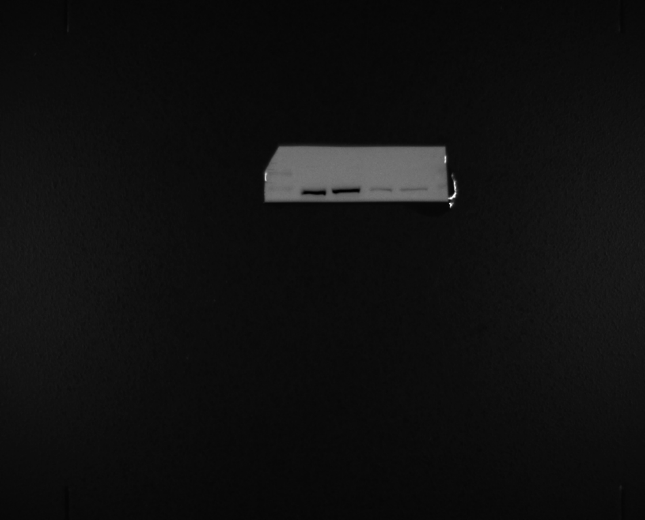


SMO


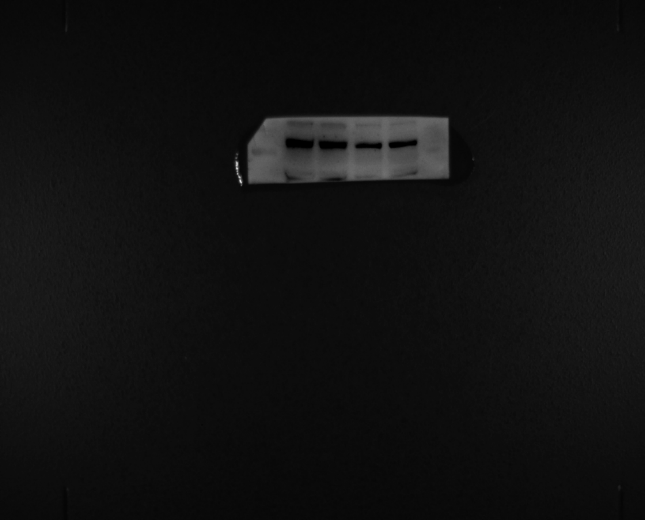


PTCH1


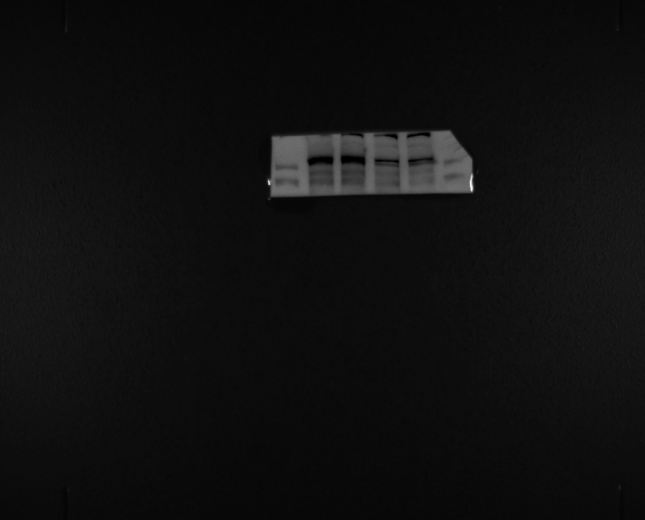


SUFU


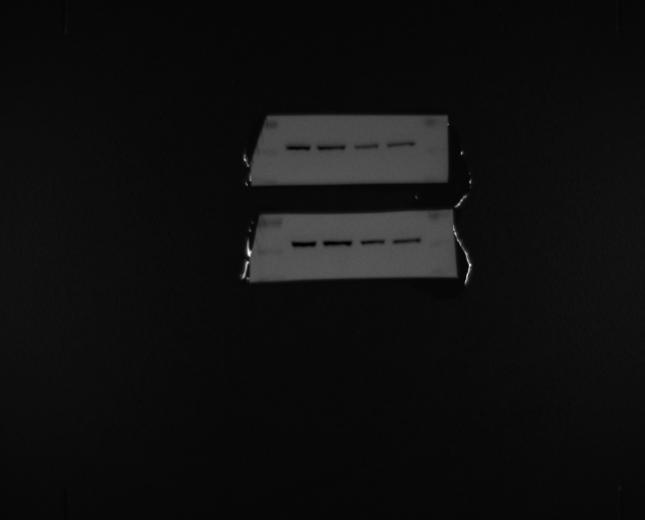


GLI2


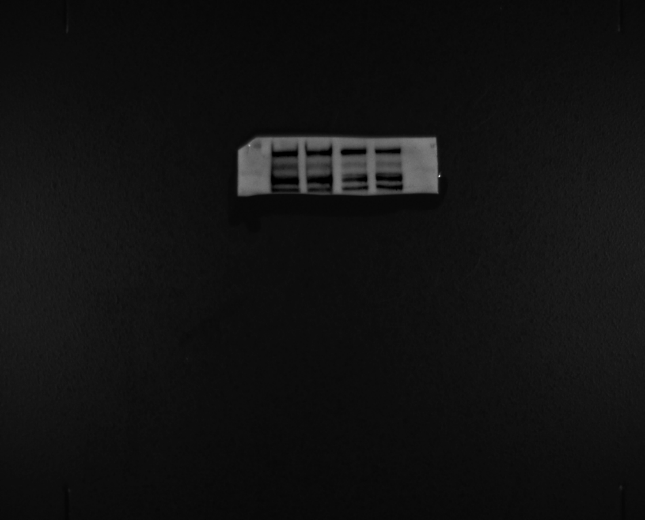


β-actin


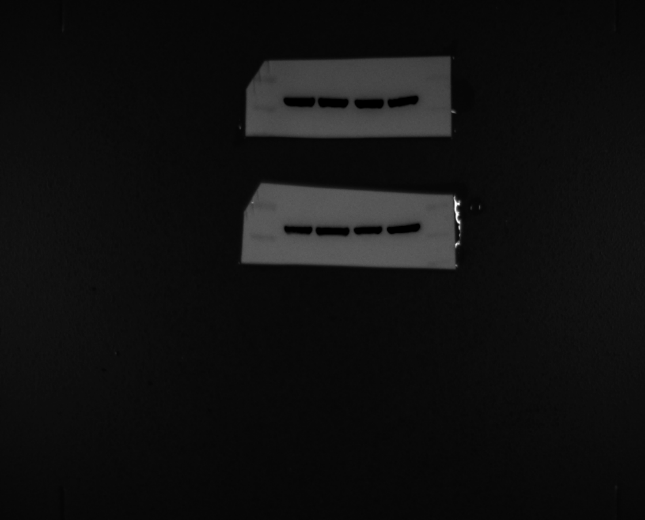


Supplementary Figure 9a

EFTUD2


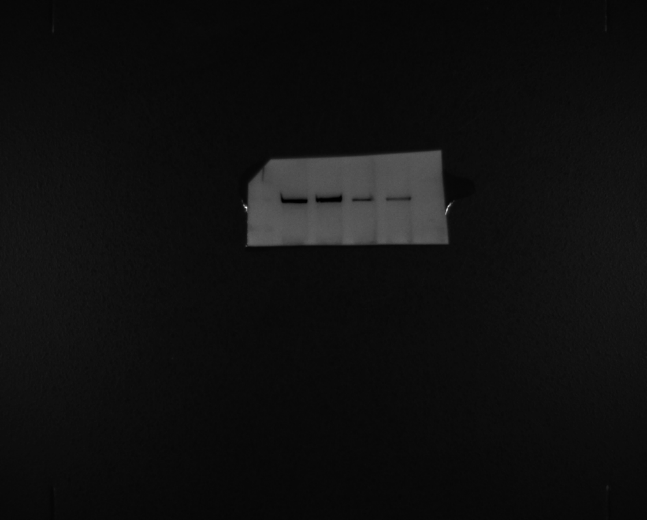


KIF3A


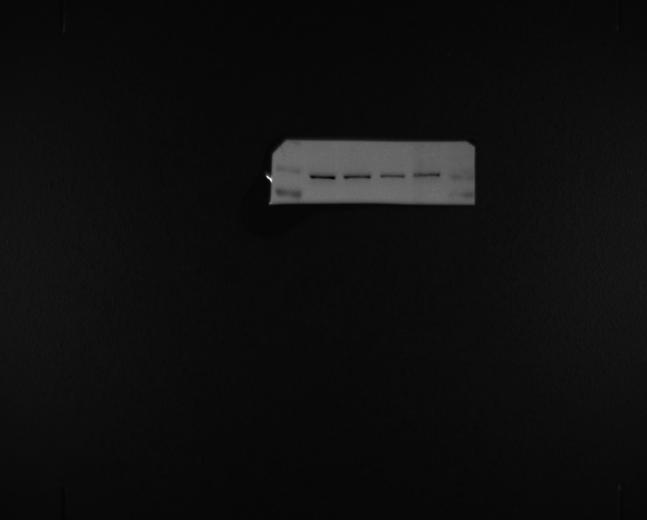


β-actin


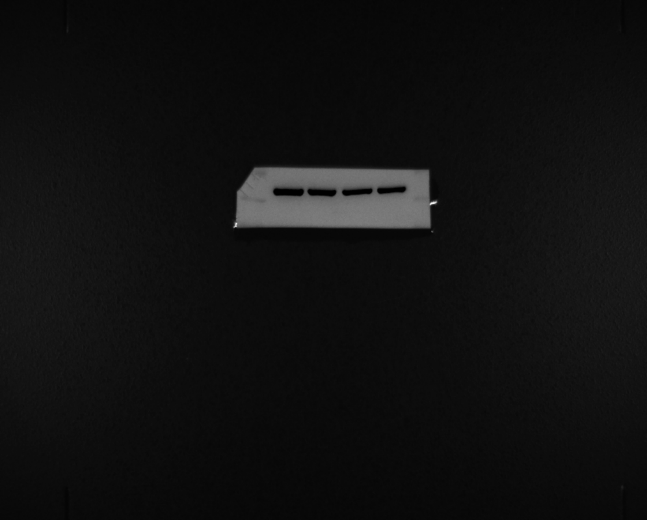


Supplementary Figure 9e

EFTUD2


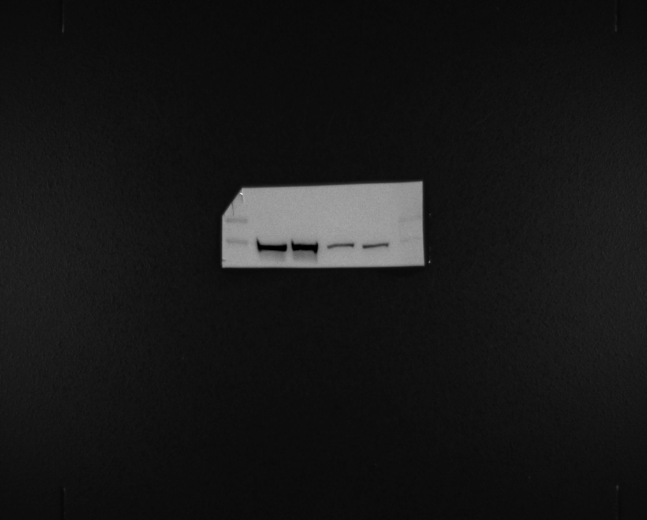


KIF3A


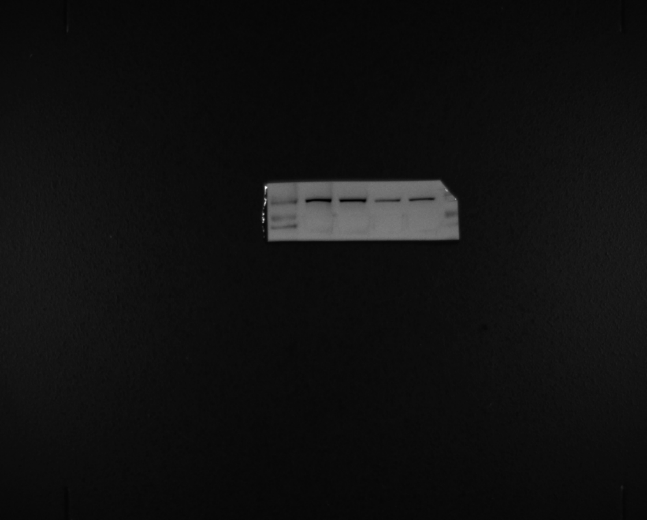


β-actin


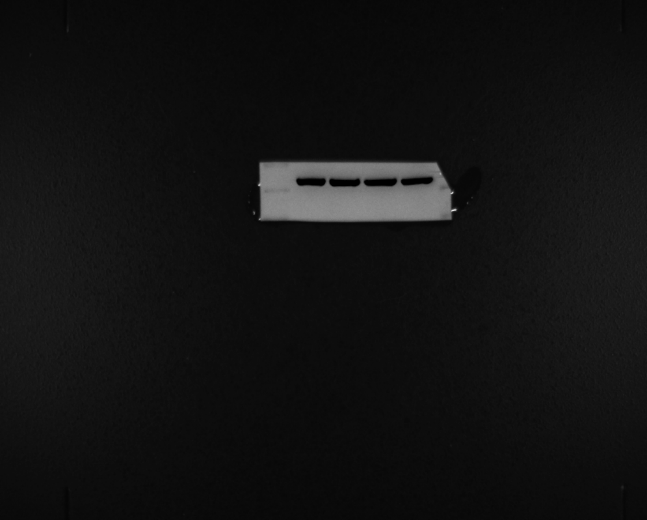


Supplementary Figure 9i

EFTUD2


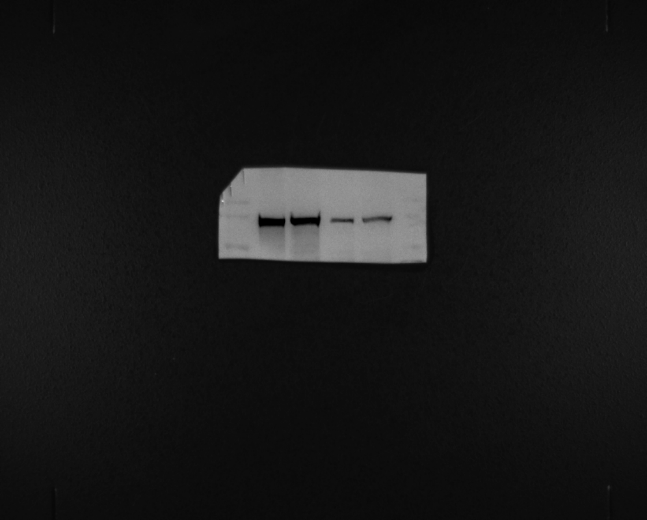


KIF3A


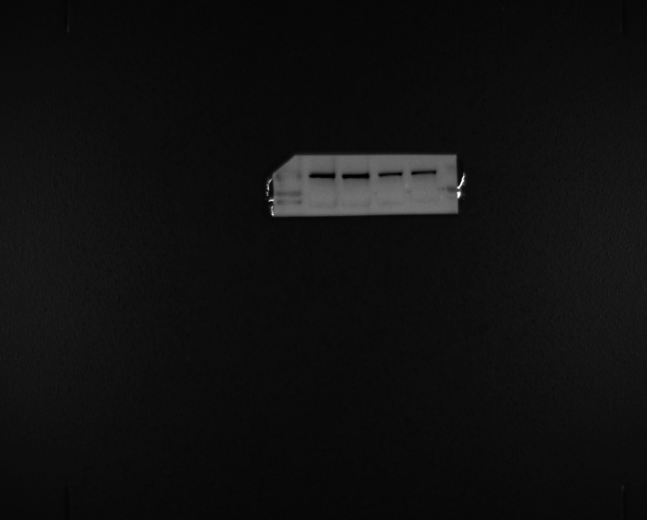


β-actin


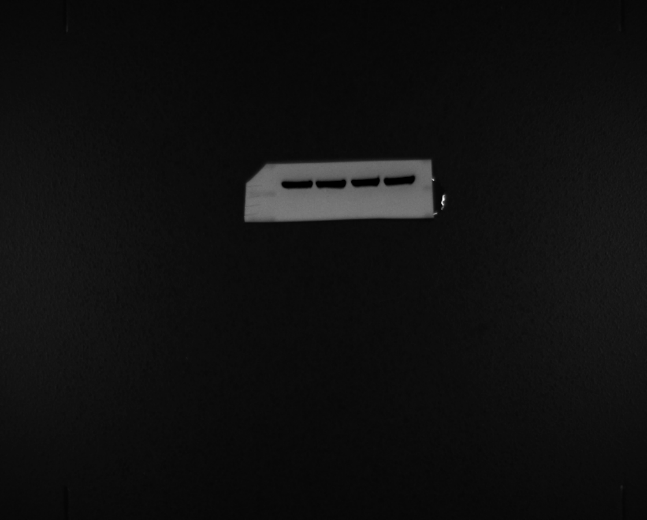


Supplementary Figure 12a


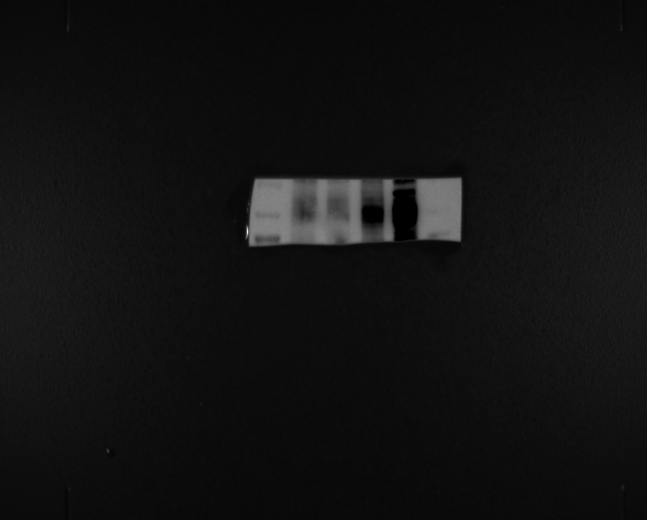

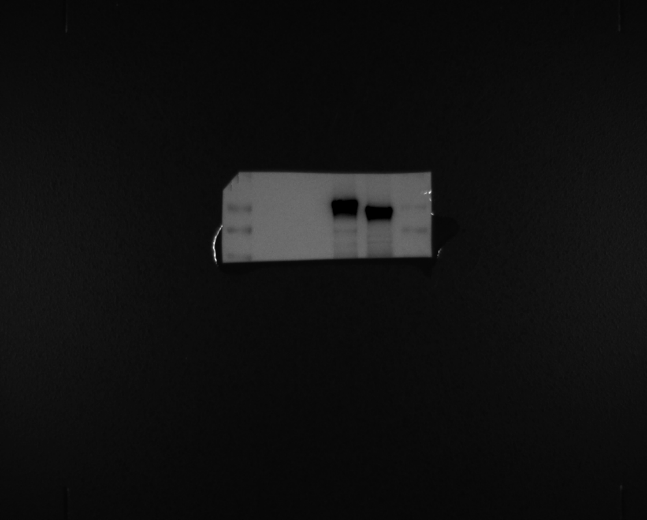
IP-KIF3A IP-p-KIF3A


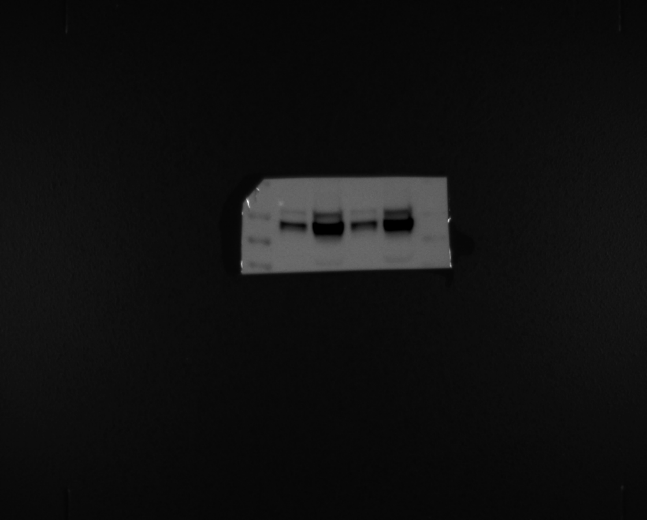
Input-KIF3A Input-p-KIF3A


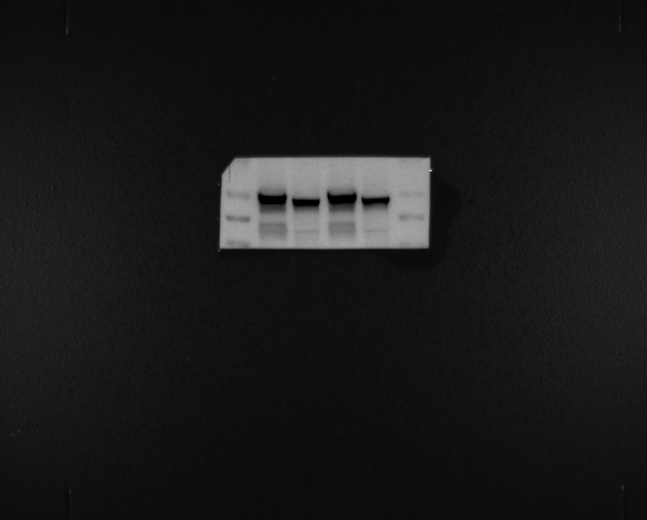


Supplementary Figure 13a


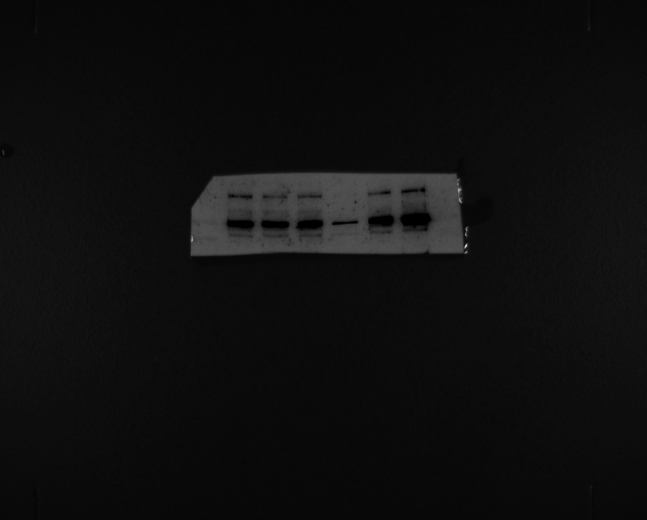
GLI1 GLI2


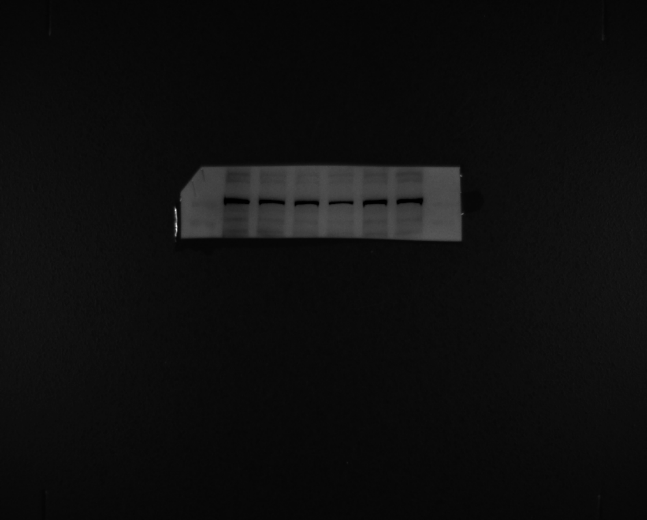


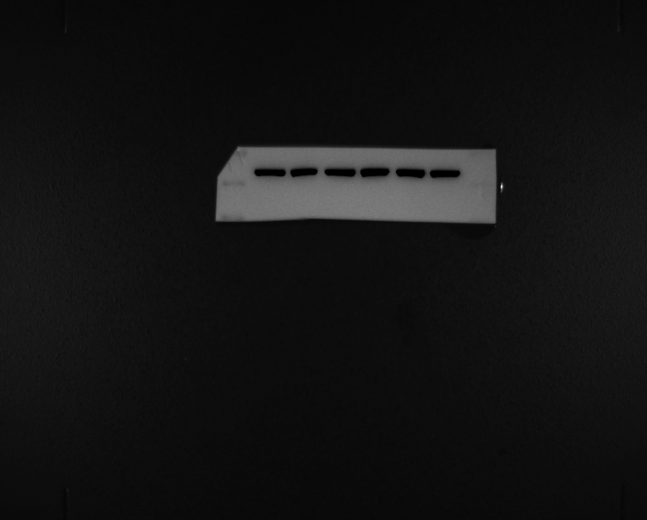
KIF3A β-actin


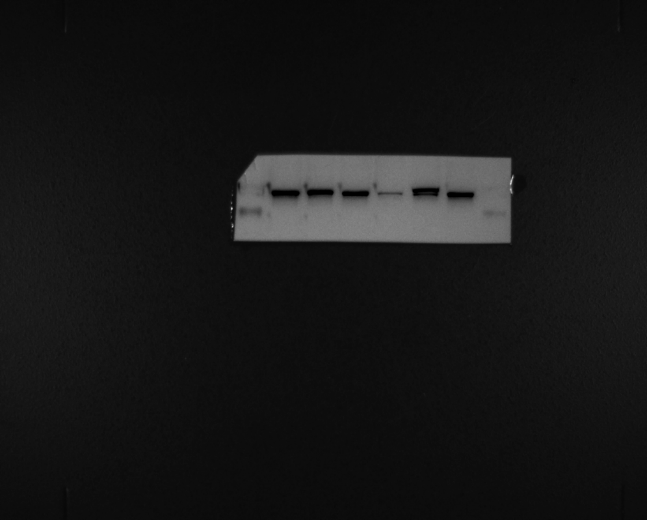


Supplementary Figure 13e


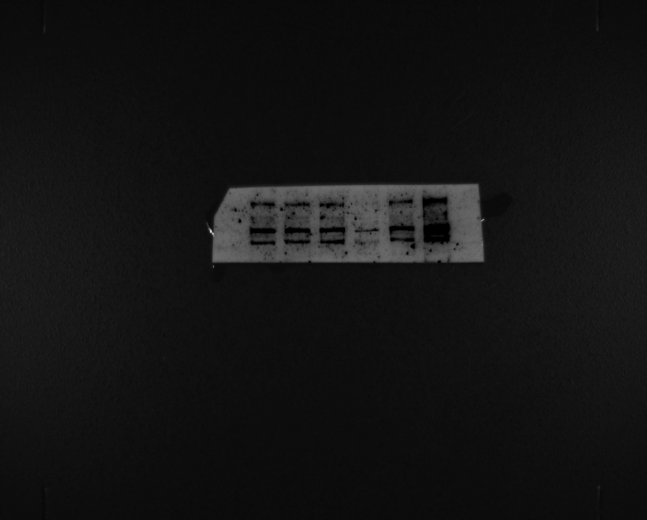
GLI1 GLI2


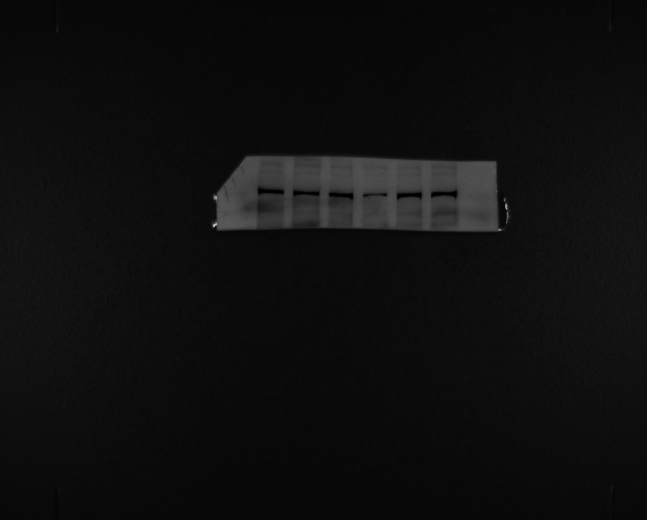


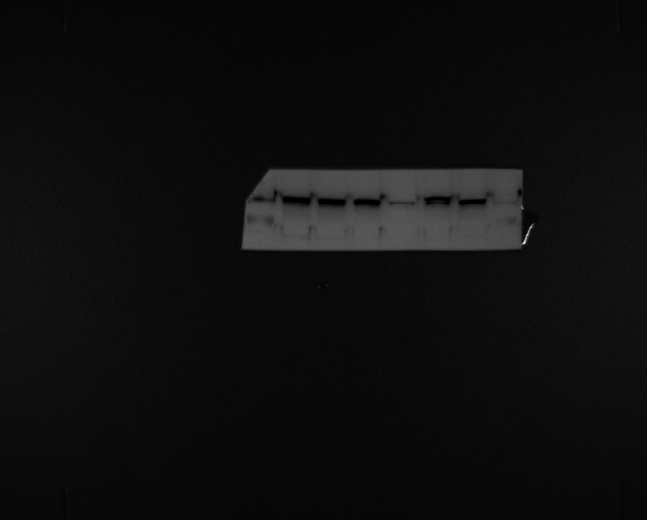

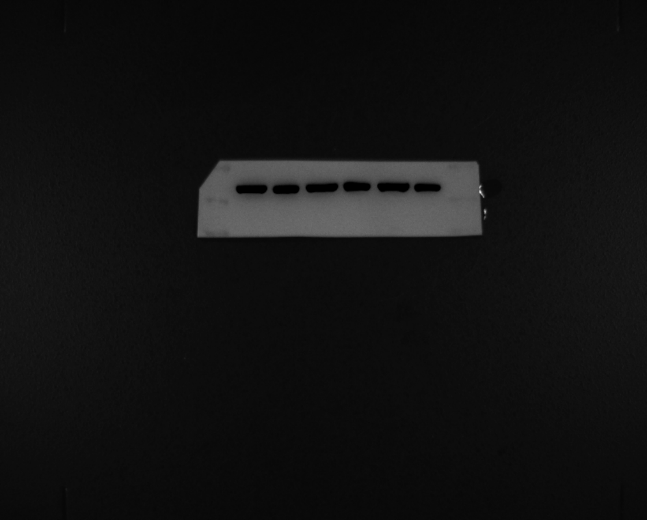
KIF3A β-actin

Supplementary Figure 16a


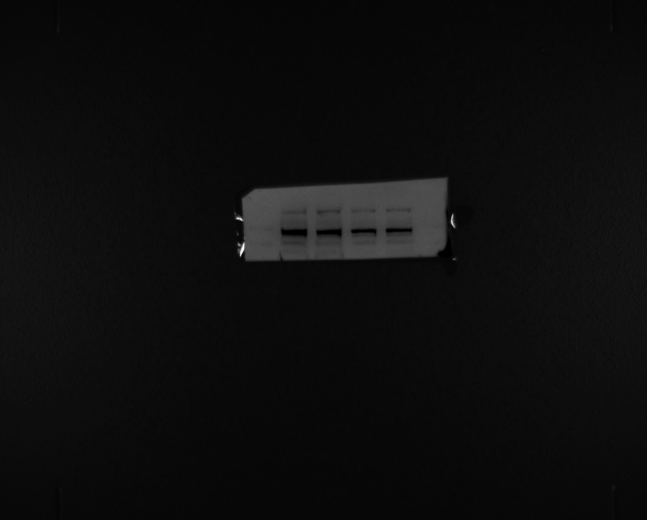

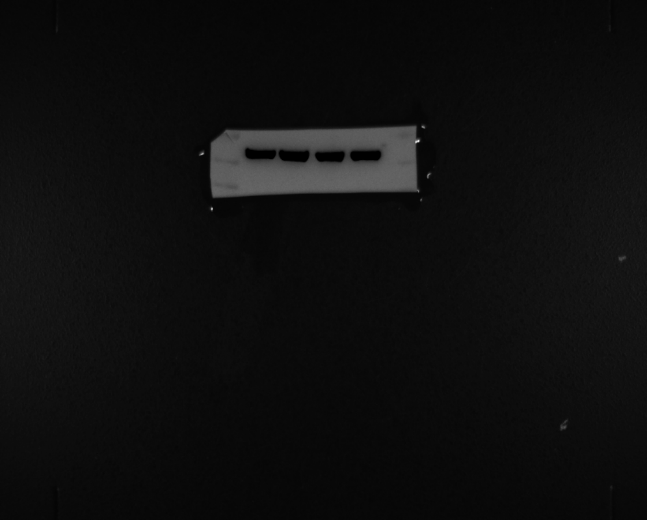
GLI2 β-actin

Supplementary Figure 16c


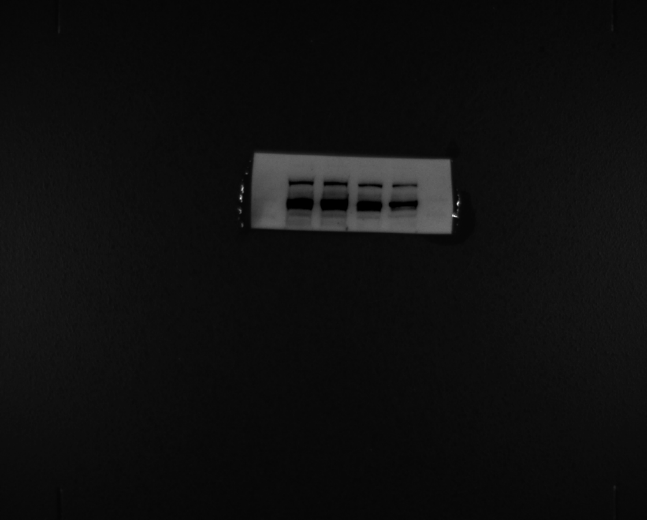

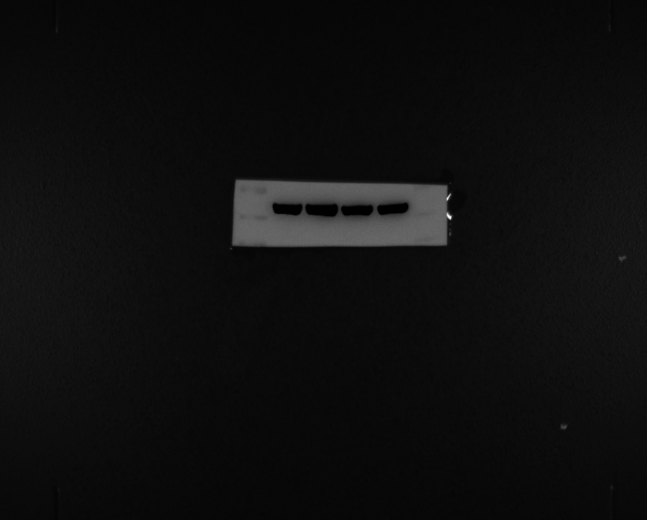
GLI2 β-actin

Supplementary Figure 16e


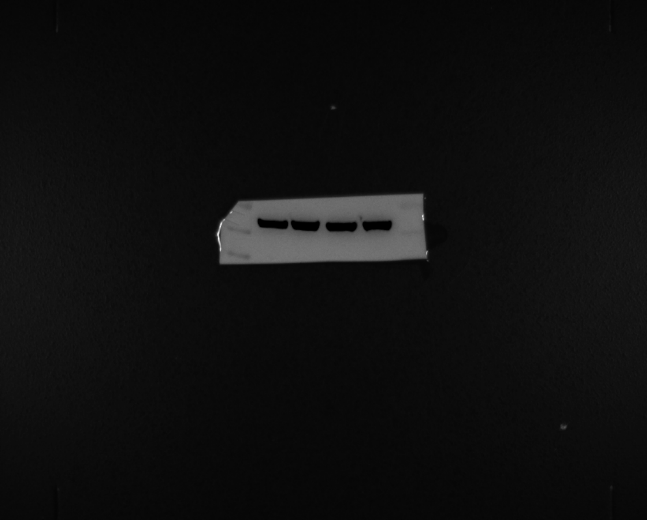
GLI2 β-actin


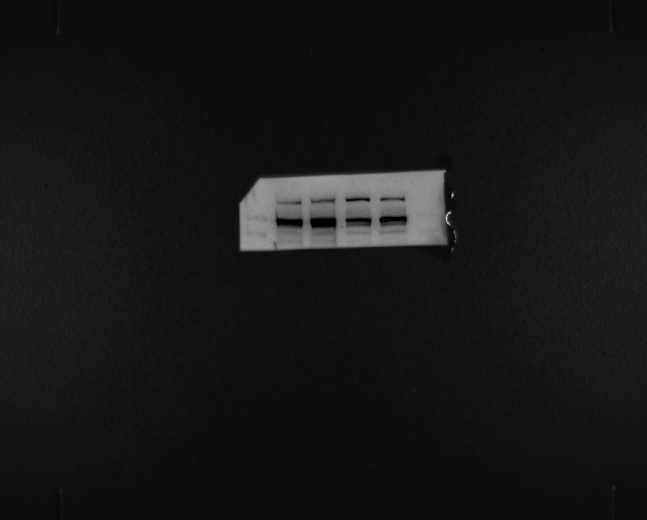

Supplement: Supplementary file 2 — Original Western blot images-20250125 [file 41418_2025_1512_MOESM2_ESM.docx]
