# Supplementary material for: Alternative spliceosomal protein Eftud2 mediated Kif3a exon skipping promotes SHH-subgroup medulloblastoma progression
Source: Cell Death Differ. 2025 Apr 24;32(10):1930–45. doi: 10.1038/s41418-025-01512-9 (PMC12501224; doi:10.1038/s41418-025-01512-9)
Supplement: Supplementary file 3 — Cell lines identification and detection-Merged [file 41418_2025_1512_MOESM3_ESM.pdf]

Sample File	Sample Name	Panel	Marker
Daoy-1_H3_A08.fsa	57	H3	01-D5S818

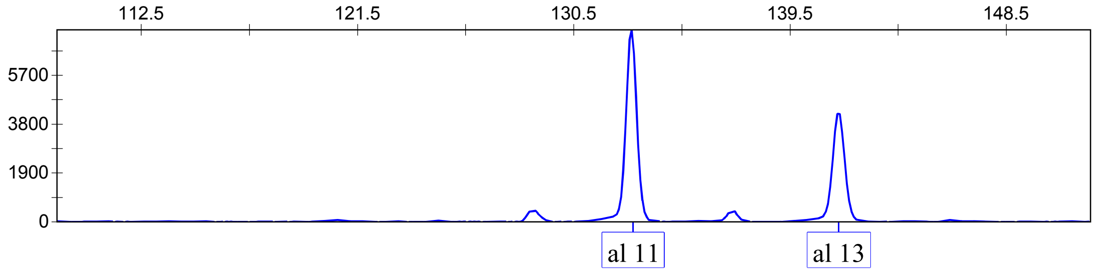

Daoy-1_H4_E08.fsa	61	H4	02-D13S317

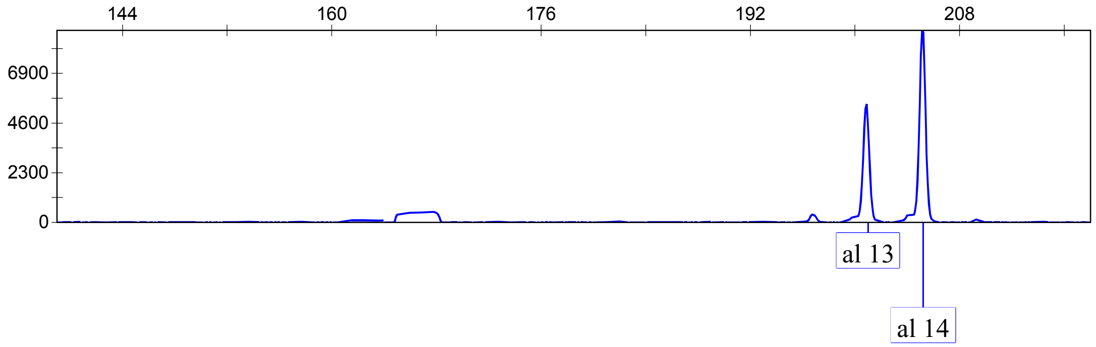

Daoy-1_H1_A07.fsa	49	H1	03-D7S820

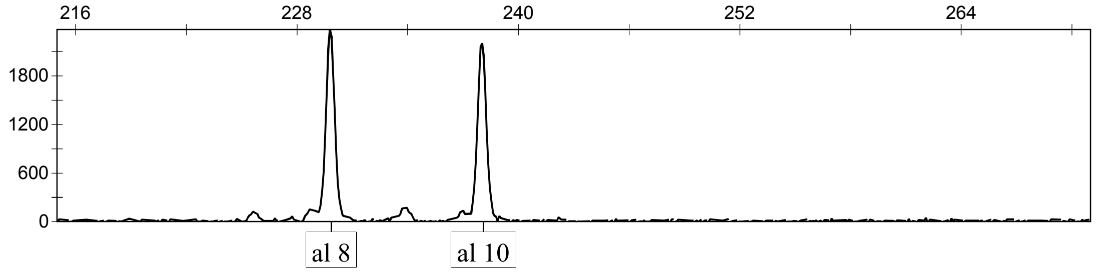

Sample File	Sample Name	Panel	Marker
Daoy-1 H3 A08.fsa	57	H3	04-D16S539

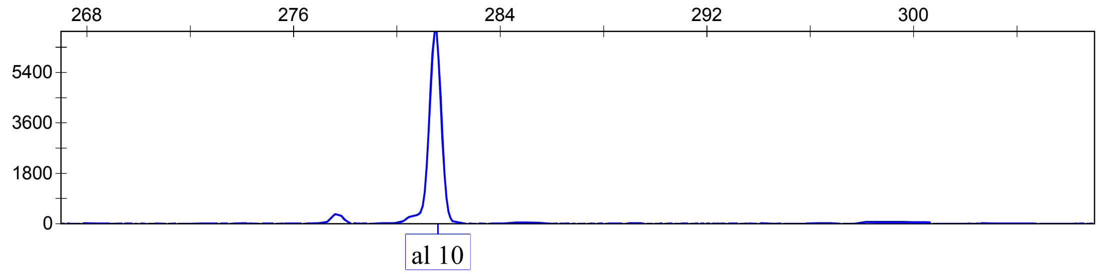

Sample File	Sample Name	Panel	Marker
Daoy-1 H3 A08.fsa	57	H3	05-VWA

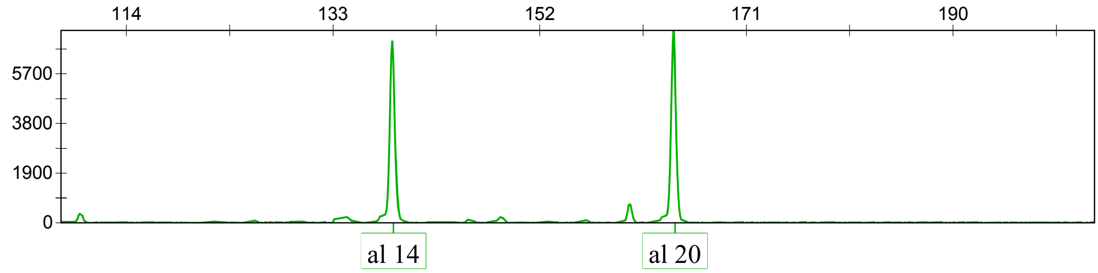

Sample File	Sample Name	Panel	Marker
Daoy-1 H1 A07.fsa	49	H1	06-TH01

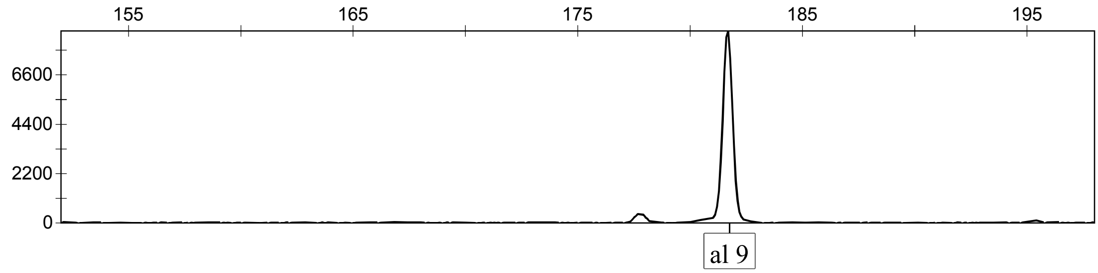

Sample File	Sample Name	Panel	Marker
Daoy-1_H1_A07.fsa	49	H1	07-Amelogenin

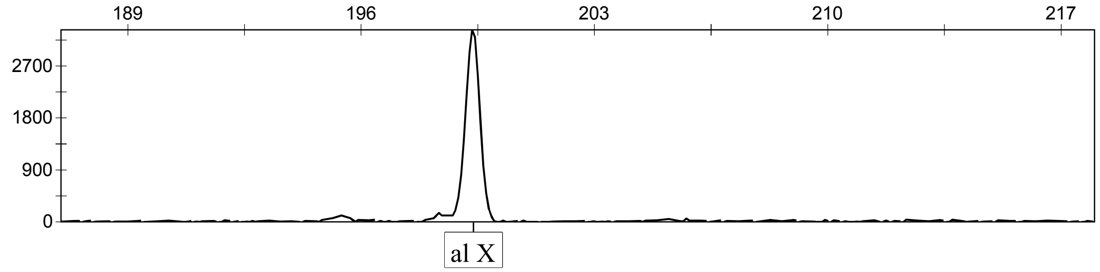

Daoy-1_H2_E07.fsa	53	H2	08-TOPX

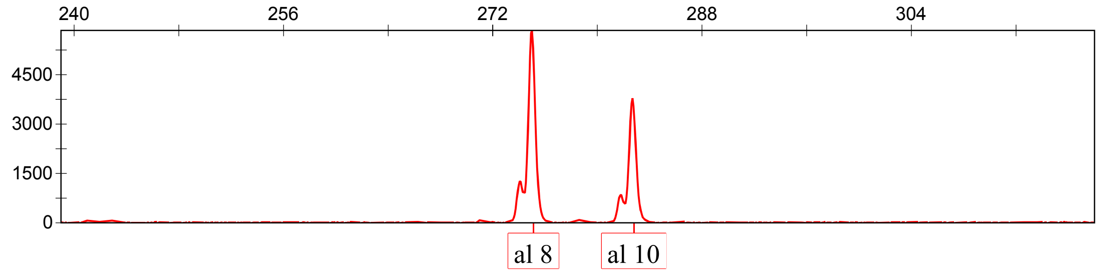

Daoy-1_H2_E07.fsa	53	H2	09-CSF1PO

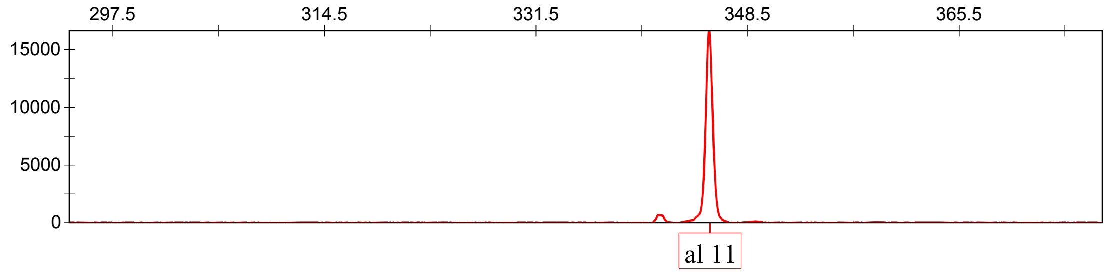

Sample File	Sample Name	Panel	Marker
Daoy-1_H3_A08.fsa	57	H3	10-D12S391

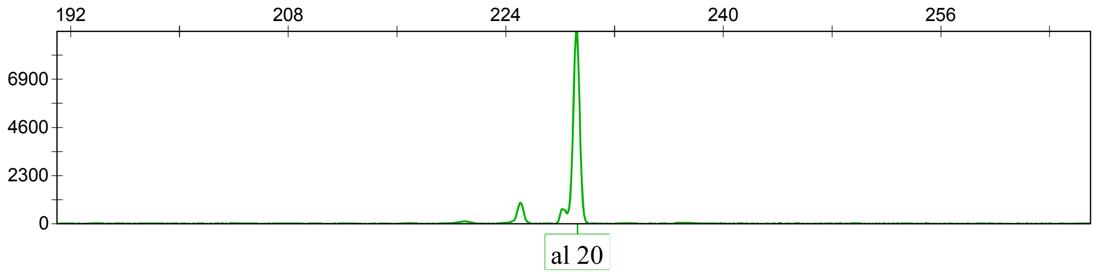

Sample File	Sample Name	Panel	Marker
Daoy-1_H1_A07.fsa	49	H1	11-FGA

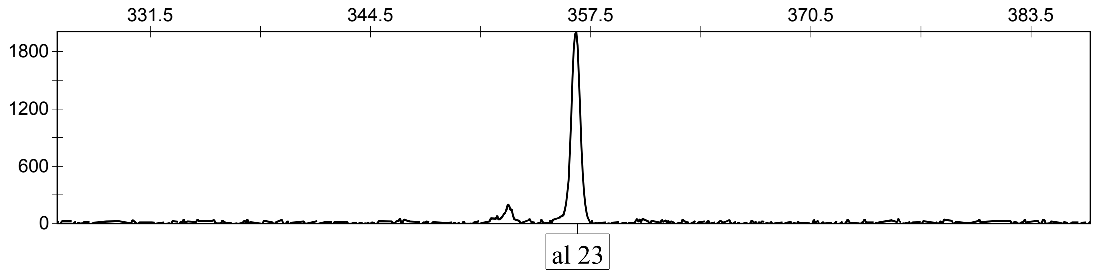

Sample File	Sample Name	Panel	Marker
Daoy-1_H4_E08.fsa	61	H4	12-D2S1338

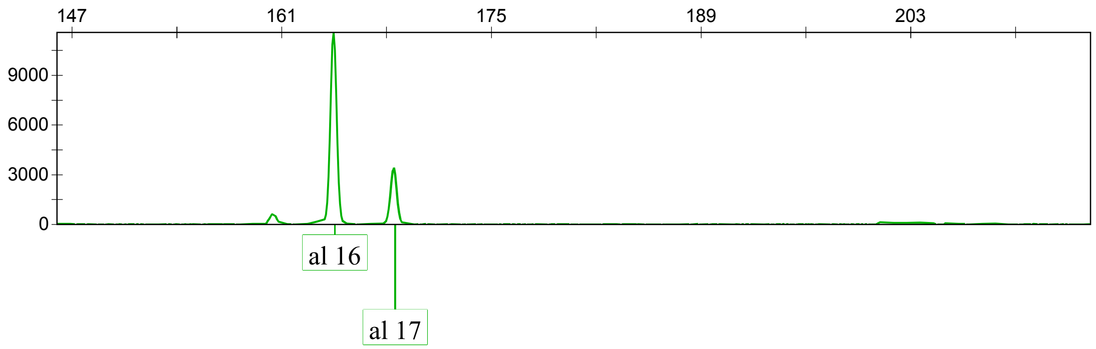

Applied Biosystems

GeneMapper Software 5

0816

Sample File	Sample Name	Panel	Marker
Daoy-1_H4_E08.fsa	61	H4	13-D21S11

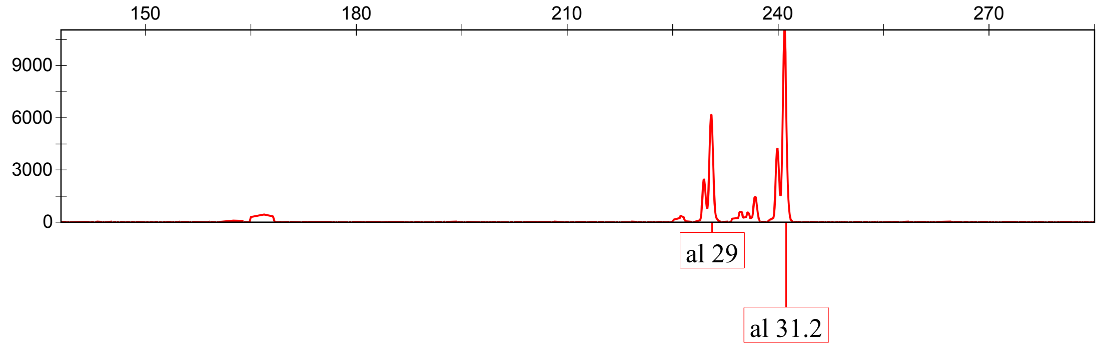

Sample File	Sample Name	Panel	Marker
Daoy-1_H3_A08.fsa	57	H3	14-D18S51

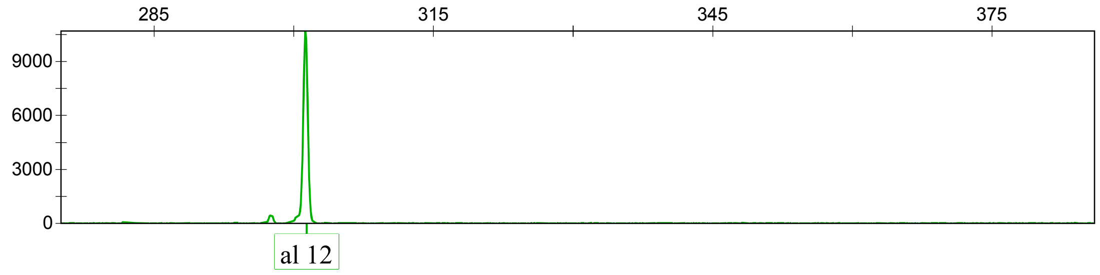

Sample File	Sample Name	Panel	Marker
Daoy-1_H3_A08.fsa	57	H3	15-D8S1179

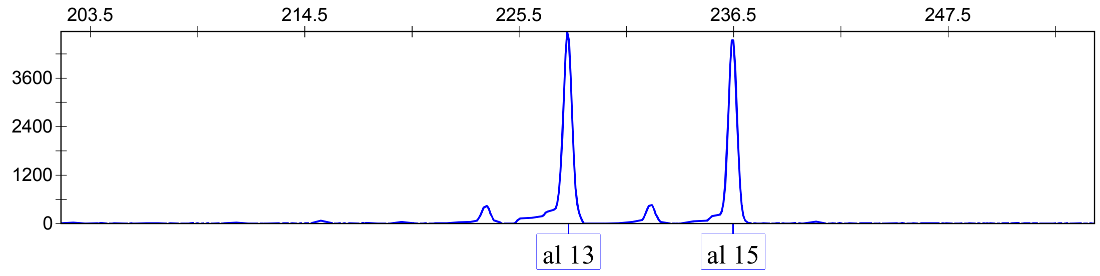

Sample File	Sample Name	Panel	Marker
Daoy-1_H2_E07.fsa	53	H2	16-D3S1358

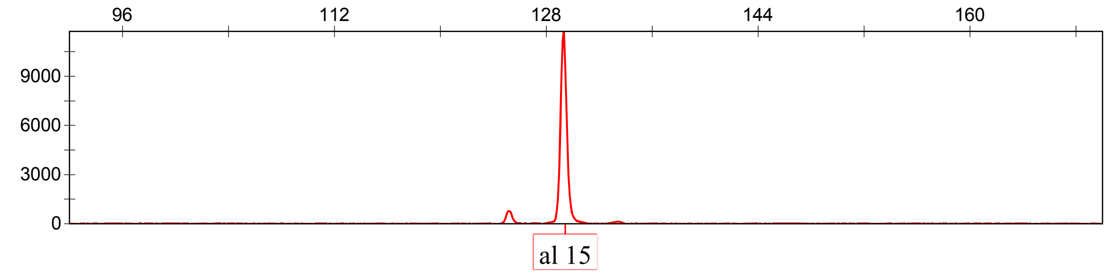

Sample File	Sample Name	Panel	Marker
Daoy-1_H1_A07.fsa	49	H1	17-D6S1043

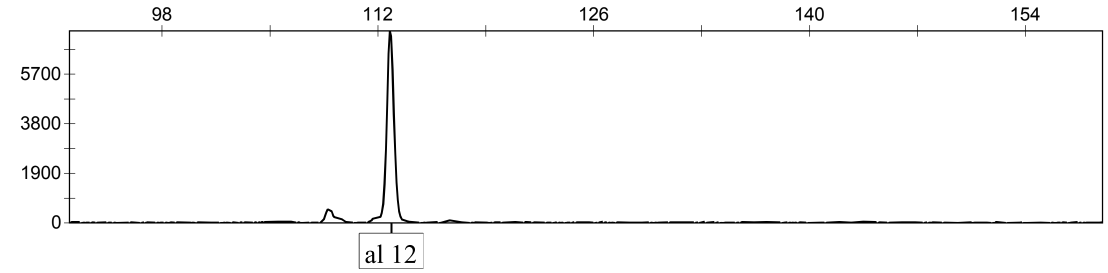

Sample File	Sample Name	Panel	Marker
Daoy-1_H4_E08.fsa	61	H4	18-PENTAE

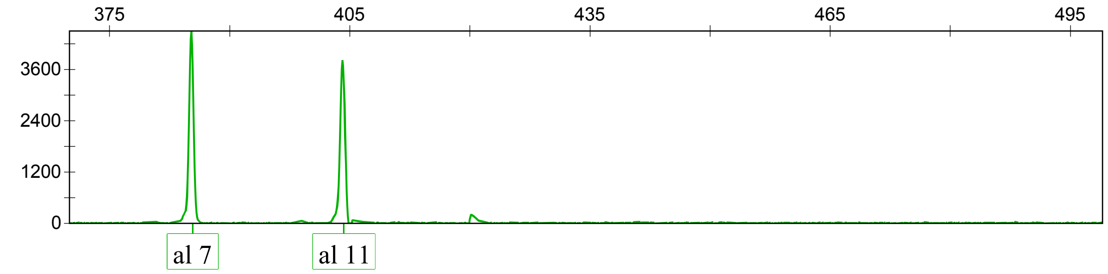

Sample File	Sample Name	Panel	Marker
Daoy-1_H2_E07.fsa	53	H2	19-D19S433

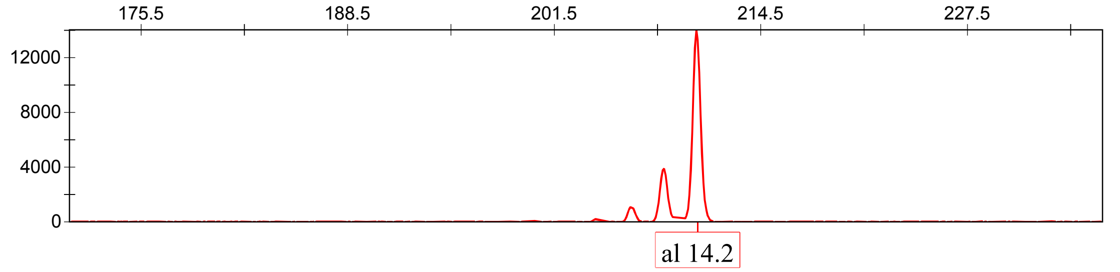

Sample File	Sample Name	Panel	Marker
Daoy-1_H4_E08.fsa	61	H4	20-PENTAD

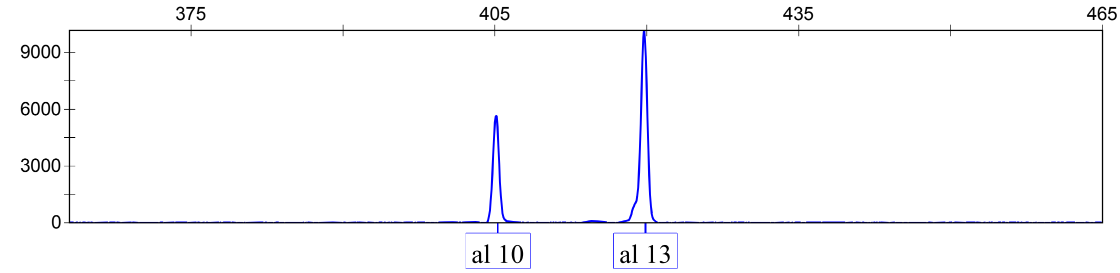

Certificate of Analysis

Cell Name	UW228	Species	Human	Biosafety level	1
Medium	The base medium for this cell line is Dulbecco's Modified Eagle's Medium,. To make the complete growth medium, add the following components to the base medium: fetal bovine serum（Gibco）to a final concentration of 10%				

Immunology	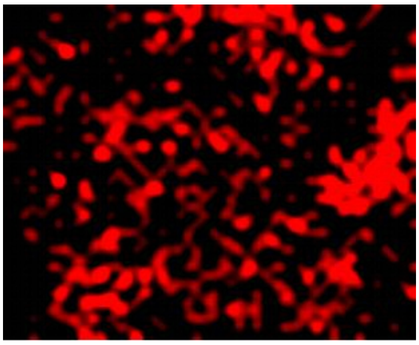 Ki67 Related Antigen :97%	Species	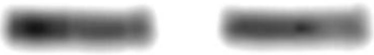 Sample Human positive control
Viability(%)	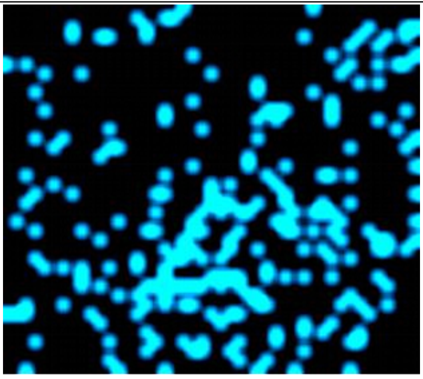 99%	MYCOPLASMA	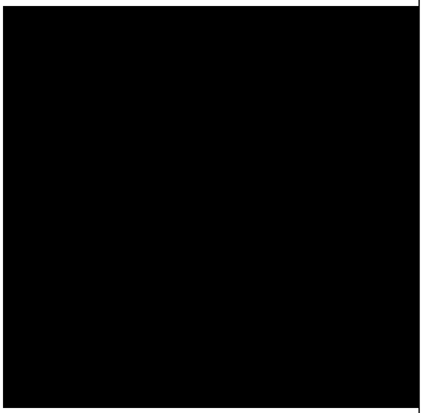 (-)

Senhao Luo PhD
Manager, Quality and Regulatory Affairs
Shanghai,China

BFB
BLUEFBIO生命科学，我们的细胞培养工厂

细 胞 STR 分型检验报告

Report of Cell Line Identification

样本编号/ Sample No.： ONS-76

待检测细胞系名称/Name of cell line: ONS-76

样本数量及规格/ Sample Spec.： 细胞沉淀 1 个/ Cell precipitation

日期/ Date： 20240708

Experimental result

Genotype Test Results

**Sample NO.**	**Multiple Alleles**	**Non - human Source Pollution**	**Matched Cell Line**
01	(−)	(−)	ONS-76

STR Typing Result

Marker	STR Profile(**sample 01**)			
	Allele1	Allele2	Allele3	Allele4
D5S818	9,10			
	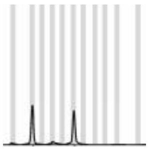			
D13S317	8,13			
	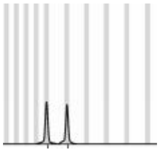			
D7S820	11,12			
	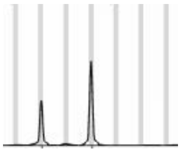			
D16S539	9,10			
	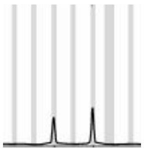			
vWA	14,16			

	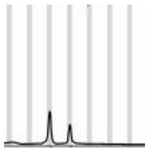
TH01	9
	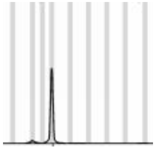
AMEL	X
	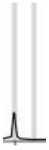
TPOX	8
	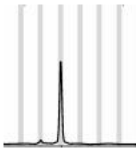
CSF1PO	10,12
	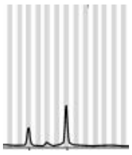

3 Programme

	Programme1	Programme2	Programme3	Programme4	Programme5
1	TH01	AMEL	TPOX	D3S1358	PENTAE
2	D12S319	D5S818	vWA	D13S317	
3	D7S820	D2S1338	D8S1179	D6S1043	
4	CSF1P0	D21S11		D16S539	
5	FGA	D18S51			

Laboratory Technician: Xin Wang

Review Member: Muyi Yang

Identification Report

Project Completion Date: August 16, 2023

Experimenter	Reporter	Reviewer
Zhang Ruiyu	Huang Wenxian	Huang Wenxian

Test Report Content

1. Objective

To conduct DNA extraction from the samples, amplify and sequence using universal bacterial 16S primers, and perform sequence alignment in the NCBI database for preliminary identification of the samples.

2. Sample Information

Sample Name	Identification Result
Daoy-1	Mycoplasma not detected
Daoy-2	Mycoplasma not detected

3. Experimental Procedure

3.1. Extraction of Genomic DNA from Cells

The genomic DNA of cells was extracted using Tsingke's Plant Genomic DNA Extraction Kit (Product Code: TSP201-200).

3.2. Amplification Using Universal 16S Primers

Amplification was performed using Tsingke's Gold Mix (Green) (Product Code: TSE101), followed by sequencing.

4. Identification Results

Both Daoy-1 and Daoy-2 samples did not yield any bands when amplified with universal 16S primers. The gel electrophoresis images are provided below.

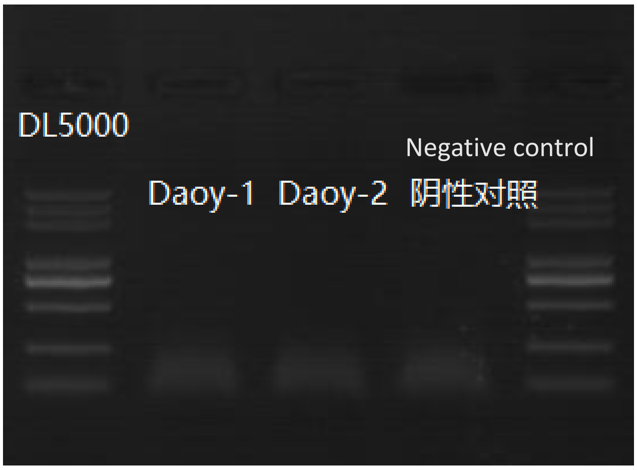

Mycoplasma Detection

1. Instruments and Equipment

Laminar flow hood, PCR machine, electrophoresis apparatus, gel imaging analysis system, benchtop centrifuge, vortex mixer, etc.

2. Experimental Reagents

Reagents include the Mycoplasma Detection Kit from Stratagene (containing primers, positive control, internal control, StrataClean resin, and buffer), dNTP, Taq DNA polymerase, buffer, agarose, and mineral oil.

3. Experimental Procedures

PCR reactions must be performed in a sterile environment.

(1) Sample Collection

Cells to be tested are cultured in antibiotic-free medium for 7 days. Collect 500 µL of the supernatant into a sterile container and store it at 4°C until testing.

(2) Template Preparation

Under sterile conditions, transfer 100 µL of the cell culture supernatant into a sterile 0.5 mL plastic centrifuge tube.

Close the cap and heat in a 95°C water bath for 5 minutes.

Open the cap and add 10 µL of StrataClean resin.

Close the cap and mix with a vortex mixer. Centrifuge for 5–10 seconds.

Transfer the supernatant to a new sterile centrifuge tube. The template preparation is complete and can be stored at 4°C.

(3) PCR Reaction

Optimal conditions for the reaction system are as follows:

Buffer composition: 10 mmol/L Tris-HCl (pH 8.3), 50 mmol/L KCl, 1.5–2.5 mmol/L $MgCl_2$, 200 µmol/L dNTPs, and 2 U Taq DNA polymerase.

Total reaction volume: 50 µL.

Use UV-irradiated (12,000 $\mu W/cm^2$) deionized water.

Steps:

Add 35.2 µL of deionized water and 5 µL of 10× Taq reaction buffer into a 0.5 mL centrifuge tube.

Sequentially add the following components:

0.4 µL dNTPs (25 mmol/L)

0.4 µL Taq DNA polymerase (5 U/µL)

2 µL primers

Add 2 µL of deionized water, bringing the total volume to 45 µL.

Add 2 µL of the prepared template to the reaction mixture.

Add 5 μL each of the positive and internal controls to their respective reaction mixtures.
Prepare a negative control tube by adding 5 μL of deionized water to the reaction system.
Add 100 μL of mineral oil to each reaction tube.

(4) Agarose Gel Electrophoresis

After the PCR reaction is completed, perform agarose gel electrophoresis with a gel concentration of 2%. Upon completion of electrophoresis, analyze the results using a gel imaging system.

(5) Results

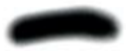

Positive control UW228 (no band, negative).

Date: 2024.08.12

BFB
BLUEFBIO生命科学，我们的细胞培养工厂

Mycoplasma Detection

1. Instruments and Equipment

Laminar flow hood, PCR machine, electrophoresis apparatus, gel imaging analysis system, benchtop centrifuge, vortex mixer, etc.

2. Experimental Reagents

Reagents include the Mycoplasma Detection Kit from Stratagene (containing primers, positive control, internal control, StrataClean resin, and buffer), dNTP, Taq DNA polymerase, buffer, agarose, and mineral oil.

3. Experimental Procedures

PCR reactions must be performed in a sterile environment.

(1) Sample Collection

Cells to be tested are cultured in antibiotic-free medium for 7 days. Collect 500 μL of the supernatant into a sterile container and store it at 4°C until testing.

(2) Template Preparation

Under sterile conditions, transfer 100 μL of the cell culture supernatant into a sterile 0.5 mL plastic centrifuge tube.

Close the cap and heat in a 95°C water bath for 5 minutes.

Open the cap and add 10 μL of StrataClean resin.

Close the cap and mix with a vortex mixer. Centrifuge for 5–10 seconds.

Transfer the supernatant to a new sterile centrifuge tube. The template preparation is complete and can be stored at 4°C.

(3) PCR Reaction

Optimal conditions for the reaction system are as follows:

Buffer composition: 10 mmol/L Tris-HCl (pH 8.3), 50 mmol/L KCl, 1.5–2.5 mmol/L MgCl2, 200 μmol/L dNTPs, and 2 U Taq DNA polymerase.

Total reaction volume: 50 μL.

Use UV-irradiated (12,000 $\mu W/cm^2$) deionized water.

Steps:

Add 35.2 μL of deionized water and 5 μL of 10× Taq reaction buffer into a 0.5 mL centrifuge tube.

Sequentially add the following components:

0.4 μL dNTPs (25 mmol/L)

0.4 μL Taq DNA polymerase (5 U/μL)

2 μL primers

Add 2 μL of deionized water, bringing the total volume to 45 μL.

Add 2 μL of the prepared template to the reaction mixture.

Add 5 μL each of the positive and internal controls to their respective reaction mixtures.
Prepare a negative control tube by adding 5 μL of deionized water to the reaction system.
Add 100 μL of mineral oil to each reaction tube.

(4) Agarose Gel Electrophoresis
After the PCR reaction is completed, perform agarose gel electrophoresis with a gel concentration of 2%. Upon completion of electrophoresis, analyze the results using a gel imaging system.

(5) Results

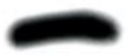

Positive control ONS-76 (no band, negative).

Date: 2024.08.12
